# Identification of a Novel Lipase with AHSMG Pentapeptide in Hypocreales and Glomerellales Filamentous Fungi

**DOI:** 10.3390/ijms23169367

**Published:** 2022-08-19

**Authors:** Denise Esther Gutiérrez-Domínguez, Bartolomé Chí-Manzanero, María Mercedes Rodríguez-Argüello, Jewel Nicole Anna Todd, Ignacio Islas-Flores, Miguel Ángel Canseco-Pérez, Blondy Canto-Canché

**Affiliations:** 1 Unidad de Biotecnología, Centro de Investigación Científica de Yucatán, A.C., Calle 43 No. 130 x 32 y 34, Colonia Chuburná de Hidalgo, Mérida C.P. 97205, Yucatán, Mexico; 2 Unidad de Bioquímica y Biología Molecular de Plantas, Centro de Investigación Científica de Yucatán, A.C., Calle 43 No. 130 x 32 y 34, Colonia Chuburná de Hidalgo, Mérida C.P. 97205, Yucatán, Mexico; 3 Dirección de Investigación, Evaluación y Posgrado, Universidad Tecnológica de Tlaxcala, Carretera a el Carmen Xalplatlahuaya s/n. El Carmen Xalplatlahuaya, Huamantla C.P. 90500, Tlaxcala, Mexico

**Keywords:** extreme enzymes, thermotolerant and solvent resistant lipases, AHSMG pentapeptide, pathogenicity factors, non-canonical effectors, filamentous fungi

## Abstract

Lipases are enzymes that hydrolyze triglycerides to fatty acids and glycerol. A typical element in lipases is a conserved motif of five amino acids (the pentapeptide), most commonly G-X-S-X-G. Lipases with the pentapeptide A-X-S-X-G are present in species of *Bacillus*, *Paucimonas lemoignei,* and the yeast *Trichosporon asahii*; they are usually thermotolerant and solvent resistant. Recently, while searching for true lipases in the *Trichoderma harzianum* genome, one lipase containing the pentapeptide AHSMG was identified. In this study, we cloned from *T. harzianum* strain B13-1 the lipase ID135964, renamed here as ThaL, which is 97.65% identical with the reference. We found that ThaL is a lid-containing true lipase of cluster III that belongs to a large family comprising highly conserved proteins in filamentous fungi in the orders Hypocreales and Glomerellales, in which predominantly pathogenic fungi are found. ThaL was expressed in conidia, as well as in *T. harzianum* mycelium, where it was cultured in liquid minimal medium. These results—together with the amino acid composition, absence of a signal peptide, mitochondrial sorting prediction, disordered regions in the protein, and lineage-specific phylogenetic distribution of its homologs—suggest that ThaL is a non-canonical effector. In summary, AHSMG-lipase is a novel lipase family in filamentous fungi, and is probably involved in pathogenicity.

## 1. Introduction

Lipases are enzymes identified with E.C. 3.1.1.3 that hydrolyze the ester bond of triglycerides to yield fatty acids and glycerol. True lipases have activity on long-chain fatty acids (triacylglycerols, TAGs), and their catalysis requires interfacial activation [1]. This phenomenon occurs when the lid that covers the catalytic site changes its conformation in the presence of a lipid–water interface; this structural switch allows the substrate to access the catalytic domain, and catalysis starts [2]. Lipases are also involved in biosynthetic reactions like esterification, interesterification and transesterification. These enzymes have characteristic domains such as the α/β hydrolase fold, a catalytic triad usually composed of serine (S), histidine (H) and aspartate (D) amino acid residues, and the oxyanion hole–[HG sequence, for histidine (H) and glycine (G)] [3].

Another typical element shared among lipases is a conserved motif of five amino acids (the pentapeptide) which contains the catalytic residue Ser (S). The most common pentapeptide is G-X-S-X-G, where X is whatever amino acid [4]. The majority of the lipases share this pentapeptide, reported in the PROSITE database as the PS00120 domain [5]. Arpigny and Jaegger (1999) [6] proposed a lipid classification based on the similarity of primary protein sequences and physiological properties, identifying eight families at that time. Currently, lipases comprise 35 families and eleven true lipase subfamilies [7,8,9].

Another classifier is the Lipase Engineering Database (LED); the LED classifies lipases into three classes according to the oxyanion hole (GX, GGGX and Y), and into 15 superfamilies based on the pentapeptide [10].

The pentapeptide is essential for lipase activity; however, little is known about its role in catalytic performance [11]. In order to explore its function, Hosseini et al. (2013) [12] replaced the pentapeptide AHSMG in the lipase BTL2 of *Bacillus thermocatenulatus* with the pentapeptide GGSAG, found in CRL lipase of *Candida rugosa*. It was observed that the native AHSMG-lipase retained 50% of its activity after 60 min at 65 °C, 80% at pH 9, and about 75% at pH 10. Additionally, it was active in the presence of detergents. Comparatively, the performance of the mutant was lower; it retained 40% of its activity after 60 min at 65 °C, and 65% and 55% of its activity at pH 9 and pH 10, respectively.

The families 1.4 and 1.5 in the Arpigny and Jaegger classification contain lipases with the pentapeptide A-X-S-X-G, where the amino acid alanine replaces the first glycine. The AHSMG pentapeptide is present in various species of *Bacillus* [13,14], in *Paucimonas* (formerly *Pseudomonas*) *lemoignei* [15], and in *Paenibacillus amylolyticusis* [16]. Interestingly, PlaA, an AHSMG-lipase from *P. amylolyticusis*, is able to degrade high-molecular-weight polylactic acid, a bioplastic that only a few bacterial enzymes can degrade [16]. Chakravorty et al. (2011) [17] compared thermostable and mesostable lipases in silico, and found that thermostable lipases contained the AHSMG pentapeptide.

Gupta et al. (2015) [3] reviewed the functional diversity of lipases in yeast and fungi; among them, they only identified one AHSMG-lipase, TaLipA, in the yeast *Trichosporon asahii*. Sequence analysis showed that it contains an unconventional oxyanion hole (GL); meanwhile, phylogenetic analysis showed that TaLipA is more related to bacterial and actinobacterial lipases than to fungal lipases [18]. TaLipA was cloned and heterologously expressed in *Pichia pastoris,* and was characterized. This lipase was thermostable, with an optimum temperature and pH of 60 °C and 8.0, respectively [18], as is consistent with the deduction of Chakravorty et al. (2011) [17] regarding the potential relationship between the AHSMG pentapeptide and thermostability.

Recently, Canseco-Pérez et al. (2018) [19] carried out an in silico genome-wide search of true lipases in the *Trichoderma harzianum* genome; among them, they found the lipase ID135964, which possesses the pentapeptide AHSMG. To the best of our knowledge, that was the first time that the AHSMG pentapeptide was identified in a lipase from a filamentous fungus, although these authors did not describe the enzyme in detail.

Here, we have cloned from the *T. harzianum* strain B13-1, the lipase that corresponds to protein ID135964 in the *T. harzianum* genome portal (https://mycocosm.jgi.doe.gov/Triha1/Triha1.home.html, accessed on 24 March 2022), called “ThaL” in this report. This protein is a true lipase with a lid and a catalytic triad composed of S, D, H, sharing a common ancestor with the AHSMG-lipases TaLipA and RN2, which are thermotolerant and resistant to alcohols and other solvents. When fungal genomes were Blasted, largely conserved orthologs were found in Hypocreales and Glomerellales orders, mostly in the former, making us suspect that this lipase may be involved in pathogenicity. In order to test this hypothesis, *T. harzianum* was cultured in liquid minimal medium, as this condition induces pathogenicity genes [20,21] and triggers antagonism [22,23]. As expected, the expression of ThaL was observed in starvation conditions, supporting a possible role of ThaL in *T. harzianum* microbial antagonism. The predicted structural analysis and mitochondrial localization, disordered regions in the protein, and lineage-specific phylogenetic distribution suggest that ThaL is a non-canonical effector [24,25]. 

In summary, this is the first report of the AHSMG-lipase family in filamentous fungi, a large and conserved novel family to which ThaL belongs. Interestingly, these lipases may have a role in fungal pathogenesis and microbial antagonism. 

## 2. Results

### 2.1. Expression of Lipase 135964 in T. harzianum

**Figure 1:**
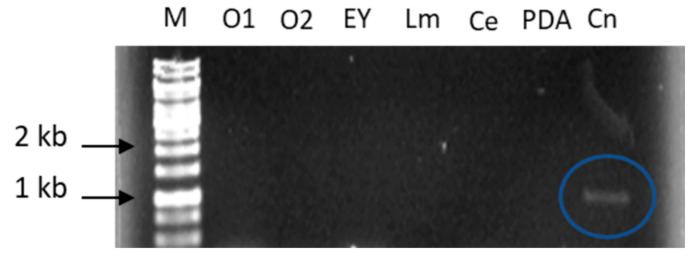
Analysis in 1% agarose gel of the expression of *T. harzianum* 135964 lipase using mycelial cDNA from different culture media and conidial cDNA as templates. M: 1 kb DNA Ladder. O1: Medium with 1% olive oil. O2: Medium with 2% olive oil. Ey: Medium with 2.5% egg yolk. Lm: Medium with 12 g/L leaf macerate *(Zephyranthes citrina*). Ce: Medium with 20 g/L cockroach exoskeleton (*Periplaneta americana*). PDA: 39 g/l medium. Cn: Conidia cDNA. The blue circle encloses the transcript of lipase135964.

In order to investigate in which conditions the 135964 lipase is expressed in *T. harzianum*, RT-PCR was conducted on cDNA prepared from mycelia cultured in different culture media (minimal medium-agar added with 1% or 2% olive oil, or with egg yolk, or leaf macerate or cockroach exoskeleton; and PDA medium), or cDNA from conidia. The expression of lipase 135964 was only observed in conidia (Figure 1). The expected size for the ORF of lipase 135964 is 1020 bp; the size of the PCR product is in the expected range (Figure 1).

### 2.2. Sequencing

The full ORF of the putative lipase 135964 was cloned in pGEM-T Easy vector; the sequencing confirmed 5′-3′ orientation, and it comprises 1023 bp, with three additional nucleotides in comparison with the 1020 bp in the sequence from *T. harzianum* CBS 226.95 v1.0 (https://mycocosm.jgi.doe.gov/pages/search-for-genes.jsf?organism=Triha1, accessed on 24 March 2022). The deduced amino acid sequence confirmed the isolation in *T. harzianum* strain B13-1 of the lipase homologous to lipase 135964, henceforth called ‘ThaL’ in this report. ThaL has one extra amino acid that corresponds to the insertion of one threonine at position 144, such that its total length is 340 amino acids; meanwhile, lipase 135964 has 339 amino acids. Curiously, at GenBank, the sequence KKO99473.1 (hypothetical protein THAR02_08412 [*Trichoderma harzianum*]) is also 340 amino acids in length, with one additional threonine, and it shares 98.23% identity with lipase 135964 from the genome portal. ThaL lipase shares 97.65% identity with lipase 135964 and 99.71% with KKO99473.1 (Table 1). ThaL differs from KKO99473.1 at the amino acid residue in position 24, where ThaL has a serine (S) while the hypothetical protein KKO99473.1 has a leucine (L).

The amino acid sequence analysis also shows several conservative changes among *T. harzianum* strains CBS 226.95 and B13-1 lipases in positions 23, 30, 140, 145,174 and 242, where ThaL has T, A, D, T, R and A, respectively, while lipase 135964 has A, T, E, S, Q and G in those positions (Figure S1).

Secondary structures and the principal motifs were identified on this multiple sequence alignment (Figure S1). ThaL lipase has six β-sheets and eleven α-helices. After β3, before the following α-helix 4, the pentapeptide AHSMG is found, which contains the catalytic serine (S). The catalytic triad is composed of S135, D276 and H298. The oxyanion hole comprises the amino acids G 69, L70, F71, G72, S155, M156, and G157, with the last three harbored in the pentapeptide

### 2.3. In Silico Characterization

As previously mentioned, the ThaL lipase is predicted to have 340 amino acids; the most abundant amino acids are leucine (37 residues, 10.9%), alanine (35 residues, 10.3%) and arginine (32, 9.4%). The minor composition corresponds to cysteine, with only one residue, corresponding to 0.3% of the total amino acid composition. ProtParam and CLC bioinformatics tools calculated a molecular mass of 37.67 kDa; the predicted isoelectric point was 9.78. The instability index (II) was computed to be 40.13, which classifies this protein as unstable. IUPred3 predicts a disordered region at amino acid positions 115–194. One potential N-glycosylation site was predicted by NetNGlyc-1.0 on the ninth residue. The aliphatic index was of 90.44, and the estimated half-life was 30 h in vitro and 20 h in yeast, according to ProtParam predictions.

SignalP server and DeepTMHMM server both predict no signal peptide for ThaL lipase; meanwhile, WolfPsort predicts mitochondrial localization.

ThaL shares 20.0% identity with TaLipA, 19.58% with RN2, and 29.28% with 2Z5G. The identity between TaLipA and RN2 is 25%. The pentapeptide AHSMG is observed in the alignment of ThaL, TaLipA and RN2, but in 2Z5G, the sequence is AHSQG (Figure S2). These lipases share conservation in part of the oxyanion hole (shown in green lines), but their catalytic triads differ because the amino acids D and H in ThaL (shown in purple *) are not observed in those positions in the other sequences (Figure S2). Although these lipases share the same or similar pentapeptides, they appear to be largely divergent in sequence from one another.

### 2.4. Orthologs and the Phylogenetic Tree

Largely conserved sequences were retrieved by ThaL from GenBank at NCBI. The statistical parameters for the first 100 hits were: a minimum coverage of 77%, an identity percentage ranging from 59.02 to 99.7%, an E value from 3.00 × 10^−130^ to 0, and a total score from 382 to 658 (Table S1).

Figure S3A corresponds to the multiple sequence alignment from ClustalW-ESPrit 3.0. Sequence divergence in these lipases was observed at the N-end, and large conservation, with similar or identical amino acids, was observed throughout the sequences of these AHSMG-lipases from filamentous fungi. Additionally, a subclass of AHSMG-lipase containing the motif QTTAASLPSAQ was observed (highlighted in yellow). When lipases TaLipA, RN2, and Z25G are included in this multi-alignment, conservation is only observed at the oxyanion hole and pentapeptide (Figure S3B), as is congruent with the previous result in Figure S2.

In order to better explore the relationship of TaLipA, RN2, and Z25G with ThaL at the primary sequence level, independent multiple alignments were performed for each one of these lipases, and also for ThaL and its top ten homologs. Each of these lipases (TaLipA, RN2, and Z25G) showed conservation with ThaL and its homologs in the pentapeptide and part of the oxyanion hole, but also in other regions (Figure S4A). However, the pattern of regions highlighted with red shadows (which correspond to the conserved regions), are different in the alignments that include TaLipA, or RN2, or 2Z5G (Figure S4A). These differences explain the lack of conservation in the alignments of AHSMG-lipases from filamentous fungi when they include at the same time these three proteins (Figure S4B), warning us to be careful with the interpretation of these results.

Immediately next to the AHSMG pentapeptide, in ThaL and its top ten homologs, is the tripeptide GLD; meanwhile, in TalipA, it is TLV; in RN2 it is GAN; and in 2Z5G, GQT is found (Figure S4B). All of the filamentous fungi retrieved by ThaL contain the tripeptide GLD after the pentapeptide AHSMG (Figure S3A).

In order to investigate the phylogenetic distribution of the ThaL homologs, the NCBI taxonomy tool was used. It was found that the fungi belong to six families (Hypocreacea, Ophiocordycipitaceae, Nectriaceae, Stachybotryaceae, Bionectriaceae, and Clavicipitaceae) in the Hypocreales order, and the Glomerellaceae family in Glomerellales order. Table 2 shows the genera and species that these fungal families comprised. The most frequent are the genera *Fusarium* (49 sequences) and *Trichoderma* (22 sequences). We observed multiple accessions in some fungal species, which were sequences with similar lengths and statistical parameters. Multiple sequence alignments were conducted in ClustalW for sequences arising from the same fungus; high conservation was observed in all of the alignments, but a few conservative changes and in/dels were observed as well (not shown), supporting the supposition that they are not redundant sequences. The alignment allowed us to identify sequences that are extremely similar, each time from the same organisms. In order to avoid bias in the following results, a single sequence from each group of almost identical sequences was arbitrarily selected to continue with the subsequent analyses.

**Figure 2:**
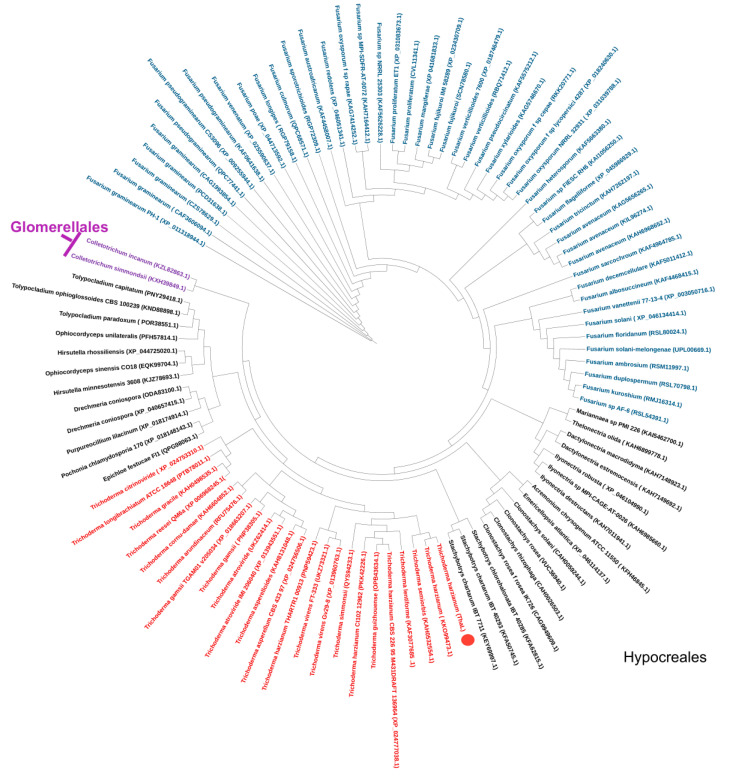
Phylogenetic tree of the ThaL lipase of *T. harzianum* and its homologs. ThaL lipase is indicated with a red circle.

The phylogenetic tree (Figure 2) shows that ThaL, as expected, groups with KKO99473.1, and it is located in the cluster of *Trichoderma. Fusarium* species constitute the largest cluster in the Hypocreales order, followed by *Trichoderma.* The sequences from Glomerellales are located at one end of the tree, next to the *Tolypocladium* clade, as represented by two *Colletotrichum* species. It is also observed that the species of each genus are grouped together, which suggests that the enzyme is transferred between nearby species. Possibly the oldest genus to have acquired it is *Fusarium*, followed by *Trichoderma*, both of which are Hypocreales. The most recent genus to acquire it is possibly *Colletotrichum* (Glomerellales), as only two species possess it.

In order to investigate the classification of the ThaL lipase, the phylogenetic tree published by Canseco-Pérez et al. (2018) [19] was reconstructed. In addition to ThaL, the top ten closest homologs and the lipases RN2, TaLipA, and 2Z5G were included. Members of the AHSMG family cluster together in cluster III, as reported by Canseco-Pérez et al. (2018) for protein ID 135964. The AHSMG-lipases group together in two sister clades, one of them comprising TaLipA (*T. asahii*) and RN2 (*Bacillus licheniformis*), and the other split into two subgroups, one for 2Z5G (*Geobacillus* sp.) and the largest one, comprising the filamentous fungal AHSMG lipases, suggesting that these lipases arise from a common ancestor. This supports the supposition that ThaL really shares a phylogenetic relationship with TaLipA, RN2 and 2Z5G, although high divergence in their sequences is observed (Figures S2, S3B and S4B).

**Figure 3:**
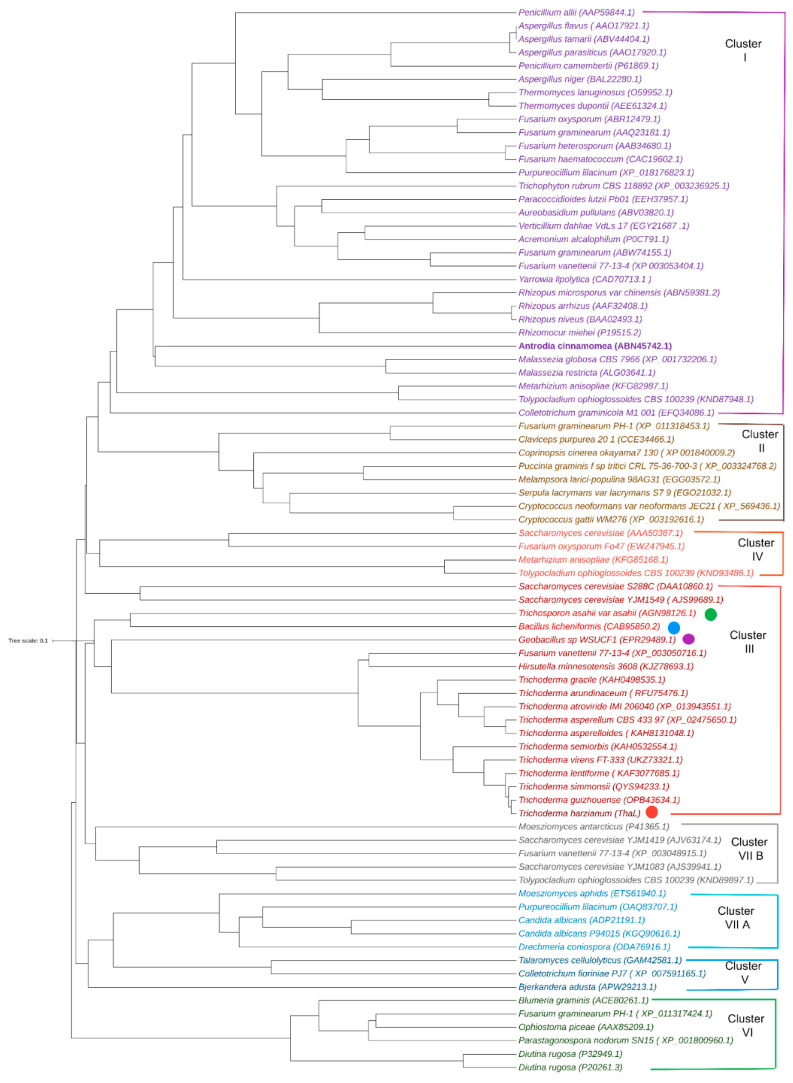
Phylogenetic tree of lipase families. The phylogenetic tree from Canseco-Pérez et al. (2018) [19] was reconstructed, and at this time the sequences of ThaL (red circle) and its top ten hits, along with TaLipA (green circle), RN2 (blue circle), and 2Z5G (purple circle), were included. The accession numbers in parenthesis in all of the sequences correspond to GenBank IDs. The tree was generated with the MAFFT program v7.0, using the UPGMA average linkage algorithm [26]. The tree was edited in iTOL v6 [27].

In order to improve on the existing knowledge of this lipase family and visualize similarities and differences with other lipases, a multiple sequence alignment was conducted with members of the AHSMG-lipase family and some members of the other clusters observed in Figure 3. Sequence divergence in the oxyanion hole and catalytic triad among the AHSMG-family and the other lipases was observed. Close to the pentapeptide, a stretch of hydrophobic amino acids (valine, isoleucine, alanine) is present in all of the lipases (Figure S5). A few motifs are unique to members of this family, such as ASLVTIATPH and ENDGLV. The tripeptide GLD, mentioned above, next to the AHSMG pentapeptide, is absent in all other families. Therefore, it is a putative marker for these filamentous fungal lipases, making it interesting for further investigations.

### 2.5. Three-Dimensional Model of the ThaL Lipase

**Figure 4:**
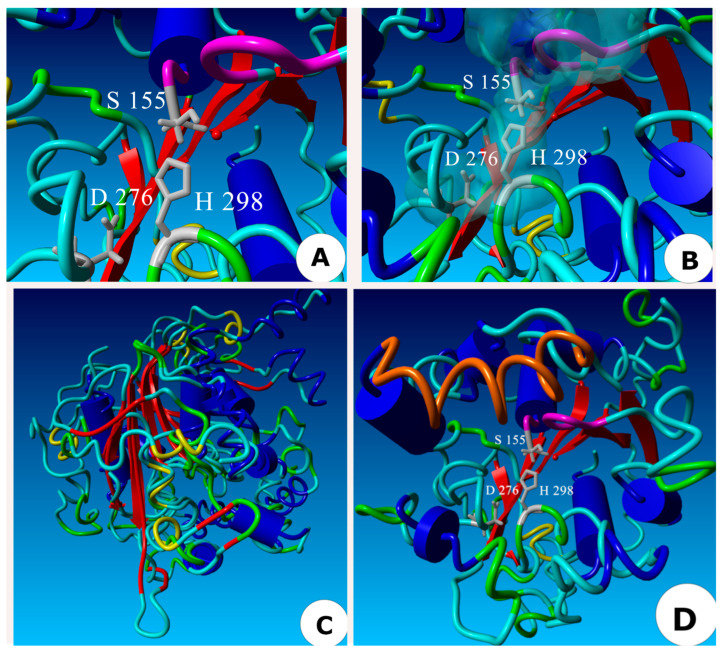
Three-dimensional model of the ThaL lipase of *T. harzianum*. (**A**) 3D model based on the protein 2Z5G from *Geobacillus zalihae* as a template; the β-sheets are in red, and α-helices are in blue. The catalytic triad, in gray. (**B**) Close up of catalytic triad and oxyanion hole; Van der Waals forces are shown as cyan clouds. (**C**) Superposition of ThaL and 2Z5G. (**D**) Lid identification in ThaL (orange) based on PDB 1EX9.

The protein in the PDB database with the closest sequence to the AHSMG pentapeptide was the 2Z5G lipase from *Geobacillus zalihae* (GenBank EPR29489.1), which has the pentapeptide AHSQG. This PDB model of 2Z5G lipase was downloaded and used as a template to generate the 3D model of ThaL (Figure 4A–C). In Figure 4A, β-sheets are in red and α-helices are in blue. The spatial distribution of the catalytic triad (S135, D276 and H298) is shown. Figure 4B is a close up showing the oxyanion hole (in purple), and the triad (in grey); the cyan clouds represent the Van der Waals forces established by interactions between amino acids from the catalytic triad and the oxyanion hole. Figure 4C shows the superposition of ThaL and 2Z5G; the β-sheets are superposed, but only a few α-helices overlap. The grey spheres correspond to the catalytic triad of ThaL. In Figure 4D, the orange region represents the lid in ThaL (A199-A214), but in this case, using PDB 1EX9 as a template. The lid is comprised of the α-helices 4, 5, 6 and 8 according to Nardini et al. (2000) [28] in their analysis of the 1EX9 protein.

### 2.6. Pathogenicity Induction by Starvation

Curiously, homologs of ThaL lipase were found to be distributed in pathogenic fungi. In order to explore whether this lipase is involved in *T. harzianum* antagonism, the fungus was grown in a liquid minimal medium, as starvation conditions mimic the environment in the host and are used to induce genes involved in pathogenesis and antagonism [29,30,31]. The expression of ThaL was evaluated by performing RT-PCR on cDNA prepared from mycelia collected on days 0, 1, 3, 5 and 7, in order to determine whether ThaL is transcribed under these conditions.

**Figure 5:**
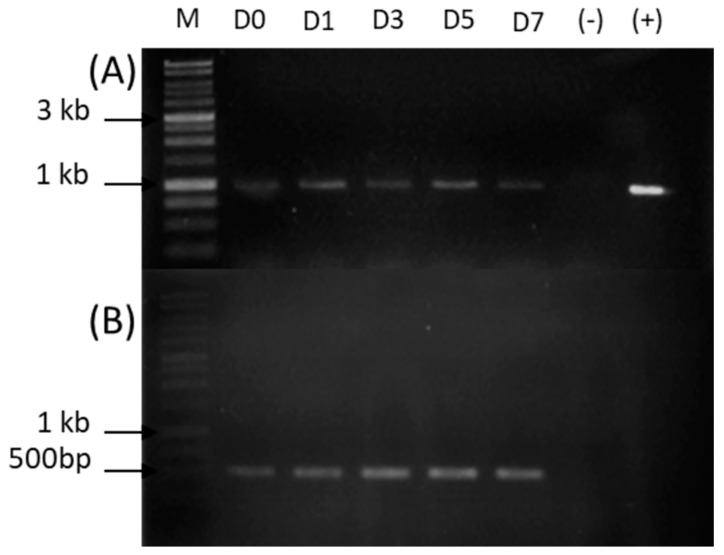
Expression of ThaL lipase *T. harzianum* cultured in liquid minimal medium. (**A**) Expression of ThaL in *T. harzianum*. (**B**) Elongation factor amplification. The temporal course corresponds to the days 0, 1, 3, 5 and 7 (labeled on the lanes as D0–D7). M: molecular marker, 1Kb DNA Ladder. (−) negative PCR control, without template. (+) positive PCR control, with gDNA as a template.

As expected from the hypothesis, ThaL expression was observed in cDNAs from *T. harzianum* mycelia, when the fungus was starved in a nutrient-poor medium (Figure 5A); the expression of ThaL was observed on day zero, that is, after the fungus was inoculated in the minimal medium and it sensed its environment. The expression of ThaL continued over the 7 days evaluated. As a reference gene, an elongation factor that is expressed constitutively was amplified from *T. harzianum* mycelia (Figure 5B).

## 3. Discussion

Lipases are versatile enzymes with a wide range of catalytic properties that make them suitable for industrial processes related to food (dairy, fat and oil, bakery, cheese flavoring, wine, meat and fish), pharmaceuticals and medicine, cosmetics, textiles, and detergents [32]. Additionally, lipases have applications in agriculture (for example, CalB lipase is used to produce the herbicide dimethenamide-P catalyzing enantioselective transamination), environmental cleaning (bioremediation, the paper industry, leather degreasing, and plastic biodegradation), bioenergy and biodiesel production, and as enzyme biosensors [32,33,34]. Nowadays, the main sources of lipases are microorganisms: bacteria and fungi. However, fungi are preferred because of their ability to degrade materials with a high content of carbon and nitrogen [35]. Thus, the relevance of these biocatalysts in industry, biotechnology and academic areas makes the search for lipase-producing fungi the focus of dynamic research.

Lipases, at the sequence level, are a very diverse class of enzymes; there are currently 35 registered families, in comparison with the eight which were initially identified [6,7]. The pentapeptide is the motif that distinguishes lipase subfamilies: family III (GXSXG) and family V (GDSAG), for example [6]. Characteristic fungal lipases harbor the pentapeptide form GXSXG.

AHSMG-lipases were reported earlier in the *Bacillus* genus, and then in the yeast *T. asahii*, although the biological function of this pentapeptide has not been determined so far. Eggert et al. (2002) [36] characterized a *Bacillus subtilis* LipB, changing alanine 76 to glycine in the AHSMG pentapeptide sequence. This mutation affected the stability of the lipase at different pHs. Moreover, Bai (2022) [11] changed the X site in the GXSXG domain of *Penicillium expansum* lipase (PEL), and observed a decrease in the activity of the mutant lipase. Liu et al. (2021) [37] found similar results in a hormone-sensitive lipase of *Pseudomonas* sp. E2-15. Together, those findings support the supposition that the substitution of any amino acid residue in the pentapeptide sequence impacts the catalytic performance of the lipase.

AHSMG lipases from *Bacillus spp*. (RN2) [13] and *T. asahii* (TaLipA) are biotechnologically attractive because they are thermotolerant (50–60 °C) and solvent resistant (to different alcohols, DMSO, ethyl acetate, toluene and hexane) [18], which makes ThaL, also with the pentapeptide AHSMG, attractive for further heterologous expression and characterization. The amino acid composition of ThaL shows that its most abundant residue is leucine, followed by alanine and arginine; these amino acids contribute to the aliphatic index of 90.44 of ThaL lipase. The aliphatic index of ThaL is higher than those found in mesophilic proteins, which suggests that ThaL is a thermophilic protein. Furthermore, the alanine and arginine residues contribute to protein stability, with alanine increasing the rigidity [20] and arginine promoting the establishment of electrostatic interactions [21]. Statistically, thermophilic proteins contain a greater amount of these two amino acids [22]. As is consistent with this hypothesis, the ThaL sequence only has one cysteine residue. This amino acid is found in disulfide bridges involved in protein folding; however, it is thermolabile, and becomes oxidized at high temperatures [23]. Usually, its presence in thermophilic proteins is very low [21].

Previously, an AHSMG-lipase was reported in the yeast *T. asahii*, but this family is novel in filamentous fungi. Curiously, the *Bacillus* genus and *T. asahii* are used in agriculture as biocontrol agents [38,39,40,41]. Whether these lipases are implicated in microbial antagonism remains to be investigated.

Canseco-Pérez et al. (2018) [19] performed a genome-wide search of true lipases, and found a lipase with the pentapeptide AHSMG, lipase 135964. In this study, we found that this lipase is distributed in eight fungal families in the order Hypocreales, and three species of the *Colletotrichum* genus (Glomerellales order). Multiple alignment and statistical data (identity 59.02–99.71%, coverage 77–100%, E-value 3.00E-128-0, and score 382–658) show that AHSMG-lipases comprise a novel, largely conserved protein family in Hypocreales and Glomerellales fungi (Figure S3A, and Table S1). It was found that lipases from filamentous fungi have distinctive motifs, such as the tripeptide GLD next to pentapeptide AHSMG, while the GLD tripeptide is not present in AHSMG-lipases of non-filamentous fungi (Figures S3B and S4B) or in other lipase families (Figure S5). Curiously, most of the fungal species with homologs of ThaL are pathogens of plants or other microorganisms. Lipases have been reported as virulence factors in both human [42] and plant fungal pathogens [33,43,44], and they have even been described as effectors in fungi [45]; most of these lipases are overexpressed during the infection of the hosts [43,44]. According to Schuster-Schmoll (2010) [46], many fungi contain enzymatic elements that allow them to establish a successful interaction with the host, with these being mainly lytic enzymes such as lipases.

Fungi can produce lipases in three ways: (1) only in the presence of an inducer (lipid); (2) without the need of an inducer, but the expression is incremented by it; and (3) constitutively [35]. In the beginning, we were unable to induce the expression of *T. harzianum* AHSMG-lipase ThaL even in the presence of classic lipase inducers (olive oil and egg yolk, among others). This lipase was only observed when RT-PCR was conducted on cDNA from conidia. The lipid contents of the spores of many fungi range from 5 to 17% dry weight, and can increase to 35% dry weight in some fungi such as rusts [47], such that the importance of lipases during conidial germination is evident. However, although the involvement of ThaL in conidial metabolism cannot be discarded, it is probably not its primary role, as it is not widely distributed in all fungal kingdoms, such as genes involved in conidial processes, e.g., autophagy. According to Keyhani (2017) [48], the recycling or assimilation of host lipids is essential for fungal invasion, and the capability of conidia to metabolize and assimilate lipids from the host might increase virulence. Conidial-specific lipases have been previously identified in *Alternaria brassicicola*, a pathogen that infects cauliflower leaves [49], and in *Nectria haematococca* (anamorph *Fusarium solani*), a pathogen that infects tomato leaves [50]. In both cases, lipases are localized on the surface of conidia. Interestingly, anti-lipase antibodies suppressed the virulence of the conidia of these fungi, and they became unable to infect host leaves; in the case of *A. brassicicola,* anti-lipase antibodies reduced 90% of the lesions on intact cauliflower leaves. The expression of ThaL only in *T. harzianum* conidia supports a potential role of this lipase during the interaction of *T. harzianum* with its hosts.

In order to explore whether ThaL is involved in pathogenicity, *T. harzianum* was cultured in a liquid minimal medium, as this mimics the environment found in the host apoplast, and induces the expression of the proteins involved in fungal pathogenesis and antagonism [29,30,31]. The transcription of ThaL was observed when *T. harzianum* was subjected to starvation. Curiously, this result is contrary to the usual behavior of lipase regulation, as nitrogen is commonly necessary in a culture medium for optimal enzyme production [51]. Here, minimal medium—which means nitrogen starvation—induced the expression of ThaL. Likewise, ThaL was not induced in the presence of different lipid substrates (Figure 1), which usually induce lipases, suggesting that the role of ThaL is not in nutrition, but it may be involved in other processes.

It was interesting that the transcript of ThaL lipase was observed when *T. harzianum* was cultured in a liquid minimal medium, but not when the fungus was cultured on solid minimal medium. It is known that the fermentation conditions influence the yield of fungal lipase production; although growth in a solid state is more similar to natural conditions, liquid culture allows the better control and homogenization of physicochemical parameters [35]. Similarly, Akao et al. (2002) [52] reported the differential expression of enzymes in *Aspergillus oryzae* in solid and liquid cultures, and these authors were able to identify the expression of specific genes related to solid culture (AOS) and others for liquid culture (AOL).

All of the fungal lipases with roles as virulence factors have been described as extracellular, and their protein sequences contain signal peptides for secretion. On the contrary, the signal peptide was not identified in *T. harzianum* ThaL lipase, even when various signal peptide predictors, including SignalP v 2.0 and v3.0 [53], were employed. Instead, ThaL was predicted to be sorted to the mitochondria.

Although early fungal effectors (microbial proteins used by the microbe to manipulate the host metabolism and their fundamental processes) were described as small secreted, cysteine-rich, extracellular proteins [54], knowledge about these pathogenicity factors has changed recently. Currently, it is known that many effectors target nuclei or mitochondrial proteins in the hosts, instead of functioning in the extracellular space [55,56]. In addition, some effectors/pathogenicity factors which have no signal peptide are secreted through multivesicular bodies (which is called an “unconventional secretion system”), which become extracellular vesicles called “virulence bags” [57,58]. The effector predictors EffHunter [54] and EffectorP [59] do not recognize ThaL as an effector (data not shown). However, some validated true effectors such as PIIN 08944 and AvrSr355, which do not meet the classical properties of effectors, are elusive to current effector predictors [54]. Therefore, the predicted mitochondrial localization and the absence of a signal peptide in ThaL do not disqualify it as a potential pathogenicity factor, and based on these findings, ThaL probably belongs to the “non-canonical effector” classification.

Other points supporting the hypothesis of an effector role for ThaL are its sequence variability in different strains (Table 1), as well as the presence of disordered regions in the protein, which was recently reported in effectors [24], and the lineage-specific phylogenetic distribution (Table 2) common to effectors [24,25].

None of the known fungal lipase virulence factors are retrieved using Blastp with ThaL as the query. One of the fungal genera in which lipases related to pathogenicity have been studied is *Fusarium*. *F. solani* surface-bound-conidial lipase (GenBank AY292529), contains the pentapeptide GHSLG [50]; meanwhile, FGL1 from *F. graminearum* (GenBank AAQ23181.1) is secreted, and contains the pentapeptide GHSLG. Therefore, functional orthology between these lipases and ThaL is not supported, suggesting that ThaL lipase belongs to a novel family involved in fungal pathogenesis.

Based on structural characteristics, that is, the oxyanion hole, catalytic triad, and the lid (Figure 4), ThaL is a true lipase, belongs to cluster III of lipases (Figure 3), and shares a common ancestor with TaLipA, RN2 and 2Z5G lipases (Figure 3). Whether ThaL is thermotolerant and solvent resistant—like the AHSMG-lipases characterized so far—needs to be determined.

In summary, the *T. harzianum* lipase, ThaL, belongs to a novel and conserved AHSMG-lipase family in (filamentous) Hypocreales and Glomerelalles fungal phytopathogens, and is probably a novel virulence factor. Further studies are necessary to confirm whether this protein is actually an effector.

## 4. Materials and Methods

### 4.1. Biological Material

*T. harzianum* strain B13-1 was used for this study. The mycelia were cultured at 25 °C in Petri dishes on potato dextrose agar (PDA), as well as minimal medium (yeast nitrogen base without amino acids and ammonium sulfate, Sigma-Aldrich) added with 1.5% agar and supplemented with 1% or 2% olive oil (*v*/*v*), 2.5% (*w*/*v*) egg yolk, 12 g/L leaf macerate (*Zephyranthes citrina*) or 20 g/L cockroach exoskeleton (*Periplaneta americana*), in order to investigate conditions where the AHSMG-lipase 135964 is expressed. In each case, the mycelia were harvested on days 1, 3 and 5, and were conserved at −80 °C until use.

For conidia production, mycelia were cultured in PDB liquid medium, harvested, filtered, and homogenized in PDB in a food processor. One milliliter of fungal fragments was placed on PDA solid medium, in the center of the Petri dish. The cultures were sealed and incubated at 23 °C and exposed to light; after 72 h, the seal was removed to allow aeration. After five days, the conidia were harvested in 2% gelatin solution by gently sweeping them with a natural hairbrush.

### 4.2. Pathogenicity Induction by Starvation

*T. harzianum* strain B13-1 was cultured in 50 mL PDB liquid medium for 5 days at 25 °C and 100 rpm. The mycelia were centrifuged at 1700× *g* for 15 min, washed three times with sterile distilled water, and centrifuged each time as above. The mycelia were inoculated in liquid minimal medium (1.7 g yeast nitrogen base without amino acids and ammonium sulfate, Sigma-Aldrich, 5 g dextrose, 100 mL distilled water). Three grams of mycelia were placed in a 250 mL flask with 50 mL minimal medium. The mycelia were harvested on days 1, 3, 5 and 7, and the samples were centrifuged at 1700× *g* for 10 min at 4 °C, weighted, and stored in liquid nitrogen.

All of the experiments were conducted with three replicates.

### 4.3. RNA Extraction and cDNA Synthesis

The total RNA was extracted from conidia or mycelia with Trizol^®^ (Invitrogen). Briefly, the samples were macerated with liquid nitrogen until a fine powder was obtained, which was then homogenized with 2 mL Trizol. The mixtures were transferred to Eppendorf tubes and incubated at room temperature for 5 min; 0.2 mL of chloroform was added, incubated for 3 min, and centrifuged at 12,000× *g* at 4 °C for 15 min. The supernatants were transferred to new tubes, and to each one 0.5 mL of isopropanol was added. They were then incubated for 10 min and centrifuged at 12,000× *g* for 10 min at 4 °C. The supernatants were discarded, and the RNAs were washed with 1 mL 75% ethanol. The pellets were air-dried for 5–10 min, and then each was resuspended in 30 µL RNAse-free distilled water. The RNA samples were frozen at −80 °C until use.

cDNAs were synthesized from 1 µg RNA; the reaction mixtures were composed of 1 µL 50 µM oligo dT, 1 µL 10 mM dNTP Mix, 4 µL 5X First-Strand Buffer, 1 µL 100 mM DTT, 1 µL RNaseOUT™ Recombinant RNase Inhibitor, and 1 µL (200 units/µL) SuperScript™ III RT, in a final volume of 13 µL. The reactions were heated at 65 °C for 5 min, and then immediately cooled in ice for 1 min; cDNA synthesis was carried out at 50 °C for 1 h. The cDNAs were aliquoted and stored at −80 °C until use.

### 4.4. Cloning and Sequencing

The full coding region of the AHSMG-lipase 135964 (ThaL) was amplified with the primers Forward 5′-ATGAAGCAATGGCCCCATTGG-3′ and Reverse 5′-TCACAATCCTTCCTTGGCCAACATG-3′ (expected size 1020 bp) in a thermal cycler BioRad T100. The reaction mixture included 7.5 µL Dream Taq Hot Start Green PCR Master Mix 2X (Thermo Fisher Scientific, Waltham, MA, USA); Forward primer and Reverse primer, 0.4 µM each; 1 µg cDNA as a template; and RNase-free ultrapure water, to a final volume of 15 µL. The PCR conditions were: 3 min at 95 °C for initial denaturation; 30 cycles of 95 °C × 30 s, 65 °C × 30 s and 72 °C × 1 min; and a final extension time of 5 min at 72 °C.

For the cloning and sequencing, the coding sequence ThaL was PCR-amplified on cDNA from conidia using the high-fidelity DNA polymerase Phusion High Fidelity (Thermo Fisher Scientific).

The PCR product was purified from gel with a QIAquick Gel Extraction kit (QIAGEN, Germantown, MD, USA), quantified in Nanodrop 2000c (ThermoScientific), and cloned into the pGEM-T Easy plasmid (PROMEGA, Madison, WI, USA) according to the supplier manual. *Escherichia coli* Top 10 chemically competent cells were transformed and then cultured in LB medium with ampicillin, X-Gal and IPTG. The positive clones were confirmed by colony PCR with M13 forward and reverse primers (Thermo Fisher).

DNA from the positive clones was extracted and purified with QIAprep Spin Miniprep kit (QIAGEN). The DNA samples were sent to Laboratorio Nacional de Biotecnología Agrícola, Médica y Ambiental (LANBAMA, IPICyT, México) for sequencing; universal T7 and SP6 primers were used for the sequencing of both DNA strands.

### 4.5. Expression Analysis

The analysis of the expression of ThaL was conducted by RT-PCR, using each of the cDNAs produced in this study and the primers described above. As a positive and loading control, the elongation factor was amplified as an endogenous reference [60].

### 4.6. Bioinformatics

#### 4.6.1. In Silico Characterization

The physicochemical properties of ThaL lipase (amino acid composition, molecular mass, charge, aliphatic index, and estimated half-life) were determined with the ProtParam tool from Expasy portal [61]. The presence of a signal peptide sequence was determined using SignalP v.6.0 [53] and DeepTMHMM servers [62]. The subcellular location of the protein was predicted using WolfPsort [63], and the disordered regions were predicted by IUPred3 [64]. The N-glycosylation was predicted by NetNGlyc-1.0 [65].

For the comparison with known AHSMG-lipases, ThaL lipase was aligned with the RN2 of *Bacillus* sp. (GenBank ABQ81810.1 [13]) and the TaLipA of *T. asahii* (GenBank AGN98126.1 [18]). Similarly, the protein 2Z5G from *Geobacillus zalihae* (GenBank EPR29489.1) was included in the analyses, as this thermoalkalophilic lipase is the crystallized protein that is retrieved by ThaL (first hit with the closest pentapeptide, AHSQG) from the PDB database.

Multiple sequence alignments were conducted by ClustalW [66], and information on the identity shared among these lipases was downloaded from the Percent Identity Matrix by Clustal2.1. Likewise, the ClustalW alignment outputs were submitted to EsPript 3.0 in order to highlight the consensus sequences [67]. For the alpha helix and beta strand prediction, the multi-alignment was analyzed using the EsPript 3.0 secondary structure depiction tool [67].

#### 4.6.2. Ortholog Identification and Phylogenetic Analysis

The Blastp analysis was performed using the default parameters and the amino acid sequence of ThaL lipase as the query against the non-redundant taxid 4751 (fungi) protein data base at GenBank (NCBI). The amino acid sequences of the first 100 hits were downloaded and used to perform an alignment in the ClustalW server using the default parameters; the output was submitted to the EsPript 3.0 program for alignment consensus visualization [67]. The statistical results for the percentage identity, coverage, E value and Total score were downloaded as a description table in the CSV format.

Lipase-conserved domains (pentapeptide and catalytic triad) were identified by manual comparison with results from Canseco-Pérez et al. (2018) [19] and Matsumura et al. (2008) [68]. The alpha helices and beta sheets were identified with EsPript 3.0 program, and are indicated in the alignments.

A phylogenetic tree was constructed using the MEGA 11.0 software for ThaL lipase, along with its homologous sequences retrieved from GenBank; the maximum-likelihood method was employed with the default settings, and 1000 bootstrap was used.

In order to classify ThaL lipase and its close homologs, the phylogenetic tree reported by Canseco-Pérez et al. (2018) [19] was reconstructed with lipase amino acid sequences reported by Gupta et al. (2015) [3] and Yadav et al. (2012) [69], along with the RN2 of *Bacillus* sp., the TaLipA of *T. asahii*, the 2Z5G of *Geobacillus zalihae*, and ThaL lipase and its top ten closest homologs retrieved in Blastp.

In order to identify all of the regions defining this fungal lipase family and the differences with other lipase families, a multiple-sequence alignment was conducted with ThaL, its top ten closest homologs, *Geobacillus zalihaev* 2Z5G lipase, and selected lipases from each lipase cluster, according to Canseco-Pérez et al. (2018) [19]. The selected lipases were as follows: cluster I, *Fusarium oxysporum* (ABR12479.1) and *Verticillium_dahliae* (EGY21687.1); cluster II, *Melampsora larici-populina* (EGG03572.1) and *Cryptococcus gattii* (XP_003192616.1); cluster III, ThaL and its homologs; cluster V, *Talaromyces cellulolyticus* (GAM42581.1); cluster VI, *Parastagonospora nodorum* (XP_001800960.1); cluster VIIA, *Candida albicans* (ADP21191.1); and cluster VIIB, *Fusarium vanettenii* (XP_003048915.1). Cluster IV was not included because it comprises very large lipases, which makes alignment difficult and prevents the observation of conserved motifs among clusters.

##### 4.6.3. 3D Modeling

The protein in the PDB database with the highest score and the closest pentapeptide to ThaL was selected as the best model; this was the protein 2Z5G fom *Geobacillus zalihaev*. Three-dimensional modeling was performed using the protein 2Z5G as a template. Alignment was conducted with the MUSTANG program [70], previously uploaded in YASARA software [71]. The identification of the catalytic triad and oxyanion hole was conducted in the alignment with the PDB model for 2Z5G. The lid was identified in ThaL by superposition with PDB 1EX9 as a template, and was based on Nardini et al. (2000) [28].

## Figures and Tables

**Table 1:** Comparison of the deduced protein sequence from the cDNA obtained in this study, with *T. harzianum* lipase 135964 at JGI and KKO99473.1 from GenBank.

Name	Source	*T. harzianum* Strain	Length (Amino Acids)	% Identity with 135964	% Identity with KKO99473.1	% Identity with ThaL
135964	CBS 226.95 v1.0, JGI	CBS 226.95	339	--------	98.23	97.65
KKO99473.1	GenBank	T6776	340	98.23	-------------	99.71
ThaL	From this study	B13-1	340	97.65	99.71	----------

**Table 2:** Taxonomic classification of organisms with orthologs of ThaL lipase of *T. harzianum*.

Order	Family	Genus	Species
Hypocreales			
	Hypocreacea		
		*Trichoderma*	*harzianum*
			*lentiforme*
			*simmonsii*
			*guizhouense*
			*virens*
			*asperellum*
			*arundinaceum*
			*asperelloides*
			*semiorbis*
			*atroviride*
			*gracile*
			*reesei*
			*longibrachiatum*
			*gamsii*
			*citrinoviride*
			*cornu-damae*
	Ophiocordycipitaceae		
		*Hirsutella*	*minnesotensis*
			*rhossiliensis*
		*Drechmeria*	*coniospora*
		*Tolypocladium*	*capitatum*
			*ophioglossoides CBS 100239*
			*paradoxum*
		*Ophiocordyceps*	*sinensis CO18*
			*unilateralis*
		*Purpureocillium*	*lilacinum*
	Nectriaceae	*Fusarium*	*AF-6(solani species complex)*
			*solani*
			*kuroshium*
			*AF-8*
			*vanettenii*
			*floridanum*
			*euwallaceae*
			*ambrosium*
			*albosuccineum*
			*decemcellulare*
			*sarcochroum*
			*flagelliforme*
			*FIESC-RH6*
			*sporotrichioides*
			*avenaceum*
			*heterosporum*
			*poae*
			*venenatum*
			*pseudograminearum*
			*graminearum*
			*MPI-SDFR-AT-0072*
			*oxyporum f. sp. rapae*
			*graminearum PH-1*
			*austroamericanum*
			*culmorum*
			*redolens*
			*longipes*
			*tricinctum*
			*austroafricanum*
			*pseudograminearum CS3096*
			*mangiferae*
			*xylarioides*
			*proliferatum*
			*proliferatum ET1*
			*oxysporum NRRL 32931*
			*fujikuroi*
			*fujikuroi IMI58289*
			*pseudocircinatum*
			*verticillioides*
			*oxysporum f. sp. lycopersici 4287*
			*oxysporum f. sp. melonis 26406*
			*oxysporum f. sp. cepae*
			*oxysporum*
			*pseudoanthophilum*
		*Ilyonectria*	*destructans*
			*MPI-CAGE-AT-0026*
			*robusta*
		*Thelonectria*	*olida*
		*Dactylonectria*	*estremocensis*
			*macrididyma*
	Stachybotryaceae	*Stachybotrys*	*chartarum*
			*chlorohalonata*
	Bionectriaceae	*Clonostachys*	*solani*
			*rosea*
			*rhizophaga*
	Clavicipitaceae	*Pochonia*	*chlamydosporia 170*
	Hypocreales incertae sedis	*Emericellopsis*	*atlantica*
		*Acremonium*	*chrysogenum*
	Clavicipitaceae	*Epichloe*	*festucae*
Glomerellales			
	Glomerellaceae		
		*Colletotrichum*	*spaethianum*
			*simmondsii*
			*sojae*

## Data Availability

Not applicable.

## Supplementary Materials

The following supporting information can be downloaded at: https://www.mdpi.com/article/10.3390/ijms23169367/s1.
