# Supplementary material for: Identification of a Novel Lipase with AHSMG Pentapeptide in Hypocreales and Glomerellales Filamentous Fungi
Source: Int J Mol Sci. 2022 Aug 19;23(16):9367. doi: 10.3390/ijms23169367 (PMC9408867; doi:10.3390/ijms23169367)
Supplement: Supplementary file 1 [file ijms-23-09367-s001.zip › Figure S1.ThaL lipase and closest hits in Trichoderma harzianum.pdf]

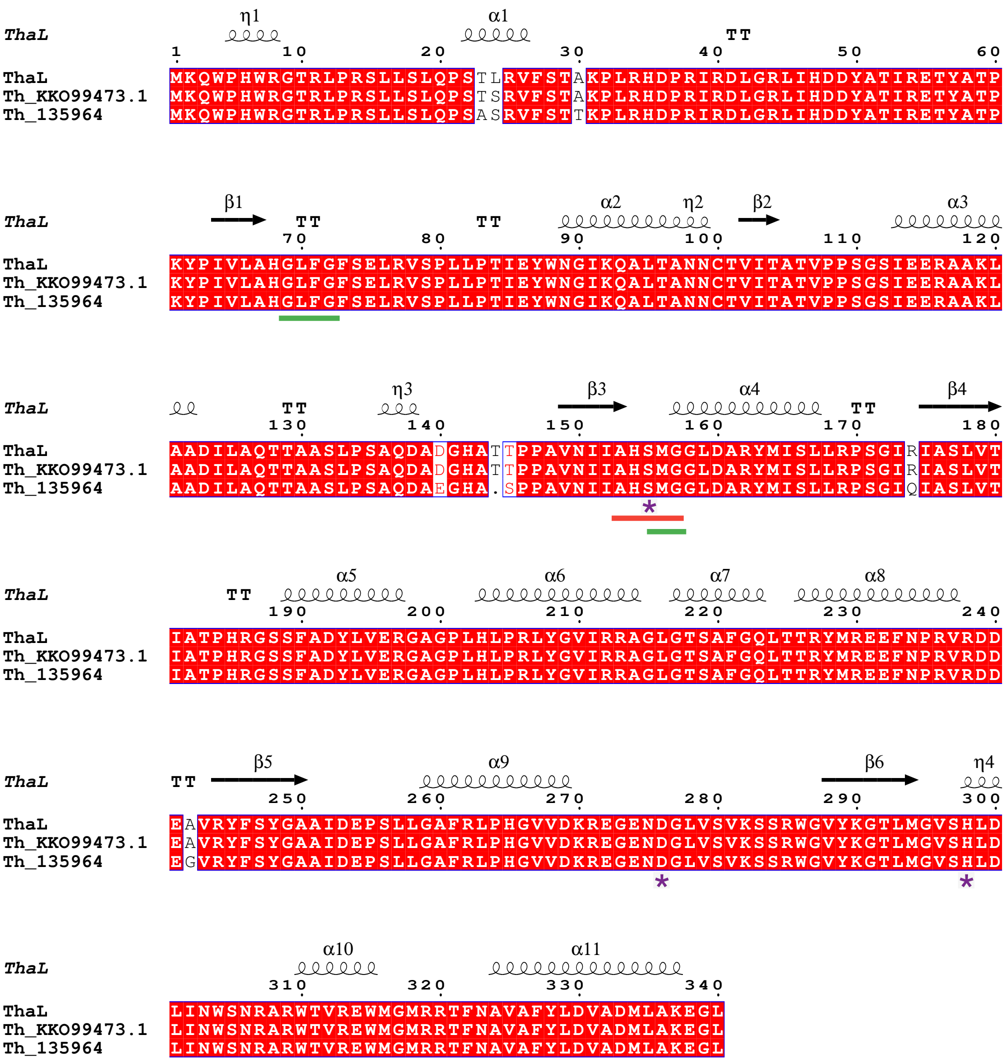

ThaL η1 α1 TT
1 10 20 30 40 50 60
ThaL MKQWPHWRGTRLPRSLLSLQPSTLRVFSTAKPLRHDPRIRDLGRLIHDDYATIRETYATP
Th_KKO99473.1 MKQWPHWRGTRLPRSLLSLQPSTSRVFSTAKPLRHDPRIRDLGRLIHDDYATIRETYATP
Th_135964 MKQWPHWRGTRLPRSLLSLQPSASRVFSTTKPLRHDPRIRDLGRLIHDDYATIRETYATP
ThaL β1 TT TT α2 η2 β2 α3
70 80 90 100 110 120
ThaL KYPIVLAHGLFGFSELRVSPLLPTIEYWNGIKQALTANNCTVITATVPPSGSIEERAAKL
Th_KKO99473.1 KYPIVLAHGLFGFSELRVSPLLPTIEYWNGIKQALTANNCTVITATVPPSGSIEERAAKL
Th_135964 KYPIVLAHGLFGFSELRVSPLLPTIEYWNGIKQALTANNCTVITATVPPSGSIEERAAKL
ThaL TT η3 β3 α4 TT β4
130 140 150 160 170 180
ThaL AADILAQTTAASLPSAQDADGHATTPPAVNIIAHSMGGLDARYMISLLRPSGIRIASLVT
Th_KKO99473.1 AADILAQTTAASLPSAQDADGHATTPPAVNIIAHSMGGLDARYMISLLRPSGIRIASLVT
Th_135964 AADILAQTTAASLPSAQDAEGHA.SPPAVNIIAHSMGGLDARYMISLLRPSGIQIASLVT
*
ThaL TT α5 α6 α7 α8
190 200 210 220 230 240
ThaL IATPHRGSSFADYLVERGAGPLHLPRLYGVIRRAGLGTSAFGQLTTRYMREEFNPRVRDD
Th_KKO99473.1 IATPHRGSSFADYLVERGAGPLHLPRLYGVIRRAGLGTSAFGQLTTRYMREEFNPRVRDD
Th_135964 IATPHRGSSFADYLVERGAGPLHLPRLYGVIRRAGLGTSAFGQLTTRYMREEFNPRVRDD
ThaL TT β5 α9 β6 η4
250 260 270 280 290 300
ThaL EAVRYFSYGAAIDEPSLLGAFRLPHGVVDKREGENDGLVSVKSSRWGVYKGTLMGVSHLD
Th_KKO99473.1 EAVRYFSYGAAIDEPSLLGAFRLPHGVVDKREGENDGLVSVKSSRWGVYKGTLMGVSHLD
Th_135964 EGVRYFSYGAAIDEPSLLGAFRLPHGVVDKREGENDGLVSVKSSRWGVYKGTLMGVSHLD
* *
ThaL α10 α11
310 320 330 340
ThaL LINWSNRARWTVREWMGMRRTFNAVAFYLDVADMLAKEGL
Th_KKO99473.1 LINWSNRARWTVREWMGMRRTFNAVAFYLDVADMLAKEGL
Th_135964 LINWSNRARWTVREWMGMRRTFNAVAFYLDVADMLAKEGL
