# Supplementary material for: Identification of a Novel Lipase with AHSMG Pentapeptide in Hypocreales and Glomerellales Filamentous Fungi
Source: Int J Mol Sci. 2022 Aug 19;23(16):9367. doi: 10.3390/ijms23169367 (PMC9408867; doi:10.3390/ijms23169367)
Supplement: Supplementary file 1 [file ijms-23-09367-s001.zip › Figure S2.Multialignment of ThaL with known AHSMG-lipases.pdf]

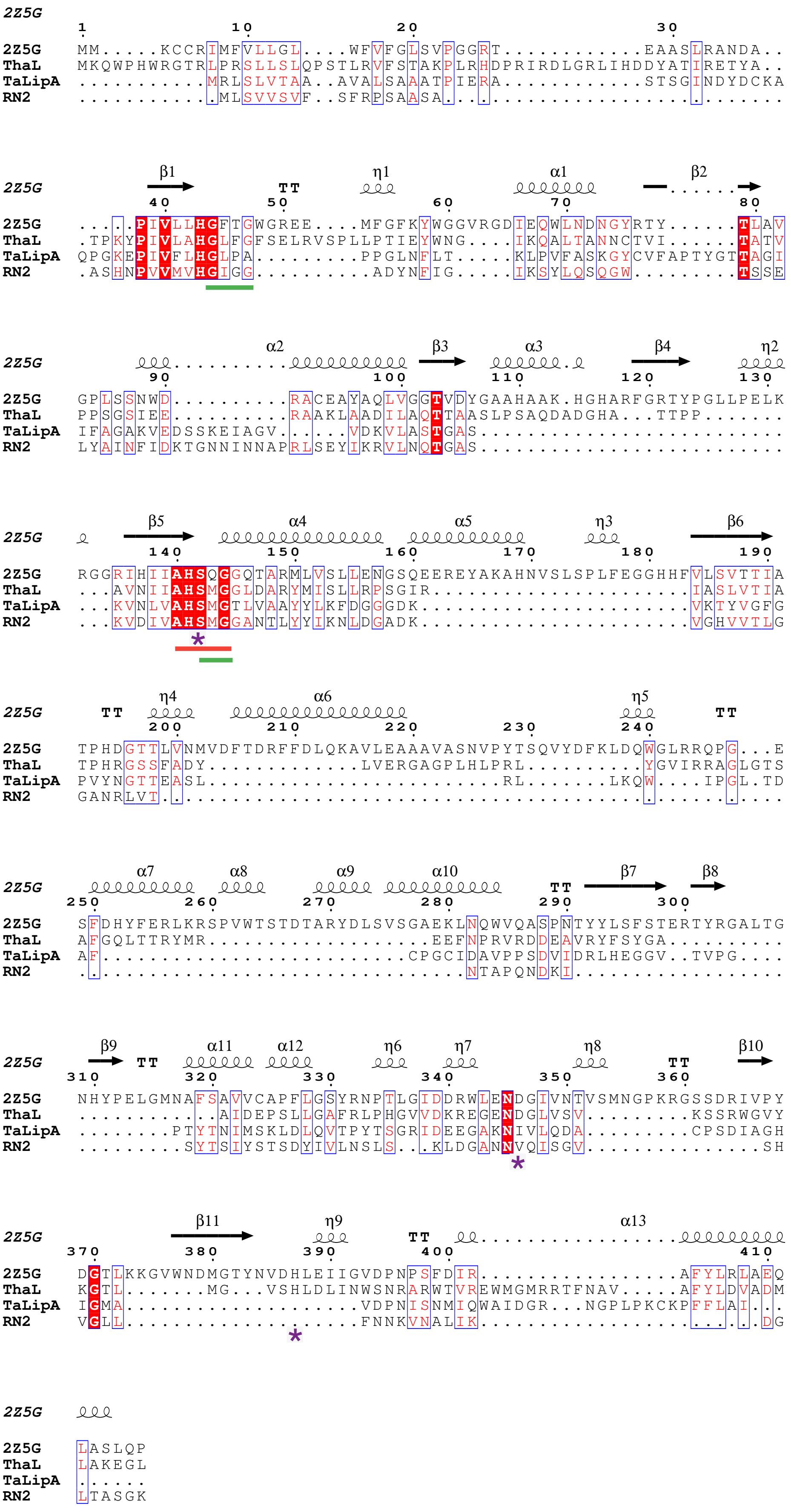
2Z5G
1 10 20 30
2Z5G MM.....KCCRIMFVLLGL....WFVFGLSVPGGRT............EAASLRANDA..
ThaL MKQWPHWRGTRLPRSLLSLQPSTLRVFSTAKPLRHDPRIRDLGRLIHDDYATIRETYA..
TaLipA ...........MRLSLVTAA..AVALSAAATPIERA...........STSGINDYDCKA
RN2 ...........MLSVVSVF..SFRPSASASA.............................
2Z5G β1 TT η1 α1 β2
40 50 60 70 80
2Z5G ....PIVLLHGFTGWGREE....MFGFKYWGGVRGDIEQWLNDNGYRTY......TLAV
ThaL .TPKYPIVLAHGLFGFSELRVSPLLPTIEYWNG....IKQALTANNCTVI......TATV
TaLipA QPGKEPIVFLHGLPA.........PPGLNFLT.....KLPVFASKGYCVFAPTYGTTAGI
RN2 .ASHNPVVMVHGIGG..........ADYNFIG.....IKSYLQSQGW.........TSSE
2Z5G α2 β3 α3 β4 η2
90 100 110 120 130
2Z5G GPLSSNWD..........RACEAYAQLVGGTVDYGAAHAAK.HGHARFGRTYPGLLPELK
ThaL PPSGSIEE..........RAAKLAADILAQTTAASLPSAQDADGHA...TTPP.......
TaLipA IFAGAKVEDSSKEIAGV.....VDKVLASTGAS........................
RN2 LYAINFIDKTGNNINNAPRLSEYIKRVLNQTGAS........................
2Z5G β5 α4 α5 η3 β6
140 150 160 170 180 190
2Z5G RGGRIHIIAHSQGGQTARMLVSLLENGSQEEREYAKAHNVSLSPLFEGGHHFVLSVTTIA
ThaL ...AVNIIAHSMGGLDARYMISLLRPSGIR.....................IASLVTIA
TaLipA ...KVNLVAHSMGTLVAAYYLKFDGGGDK......................VKTYVGFG
RN2 ...KVDIVAHSMGGANTLYYIKNLDGADK......................VGHVVTLG
*
2Z5G TT η4 α6 η5 TT
200 210 220 230 240
2Z5G TPHDGTTLVNMVDFTDRFFDLQKAVLEAAAVASNVPYTSQVYDFKLDQWGLRRQPG...E
ThaL TPHRGSSFADY............LVERGAGPLHLPRL..........YGVIRRAGLGTS
TaLipA PVYNGTTEASL........................RL.......LKQW....IPGL.TD
RN2 GANRLVT...
2Z5G α7 α8 α9 α10 TT β7 β8
250 260 270 280 290 300
2Z5G SFDHYFERLKRSPVWTSTDTARYDLSVSGAEKLNQWVQASPNTYYLSFSTERTYRGALTG
ThaL AFGQLTTRYMR...................EEFNPRVRDDEAVRYFSYGA.........
TaLipA AF.........................CPGCIDAVPPSDVIDRLHEGGV..TVPG....
RN2 ....NTAPQNDKI.................
2Z5G β9 TT α11 α12 η6 η7 η8 TT β10
310 320 330 340 350 360
2Z5G NHYPELGMNAFSAVVCAPFLGSYRNPTLGIDDRWLENDGIVNTVSMNGPKRGSSDRIVPY
ThaL ..........AIDEPSLLGAFRLPHGVVDKREGENDGLVSV.........KSSRWGVY
TaLipA ........PTYTNIMSKLDLQVTPYTSGRIDEEGAKNIVLQDA.........CPSDIAGH
RN2 .........SYTSIYSTSDYIVLNSLS..KLDGANNVQISGV...............SH
*
2Z5G β11 η9 TT α13
370 380 390 400 410
2Z5G DGTLKKGVWNDMGTYNVDHLEIIGVDPNPSFDIR................AFYLRLAEQ
ThaL KGTL.......MG...VSHLDLINWSNRARWTVREWMGMRRTFNAV.....AFYLDVADM
TaLipA IGMA.....................VDPNISNMIQWAIDGR...NGPLPKCKPFFLAI...
RN2 VGLL...................FNNKVNALIK.......................DG
*
2Z5G
2Z5G LASLQP
ThaL LAKEGL
TaLipA ......
RN2 LTASGK
