# Supplementary material for: Identification of a Novel Lipase with AHSMG Pentapeptide in Hypocreales and Glomerellales Filamentous Fungi
Source: Int J Mol Sci. 2022 Aug 19;23(16):9367. doi: 10.3390/ijms23169367 (PMC9408867; doi:10.3390/ijms23169367)
Supplement: Supplementary file 1 [file ijms-23-09367-s001.zip › Figure S3A.Multialignment of ThaL and its homologs, without TaLipA, RN2 and 2Z5G..pdf]

[Trichoderma_harzianum]_ThaL

```
                                                                                              1
[Trichoderma_harzianum]_ThaL                              ........................................................MKQ
[Trichoderma_harzianum]_KKO99473.1                        ........................................................MKQ
[Trichoderma_guizhouense]_OPB43634.1                      ........................................................MKQ
[Trichoderma_simmonsii]_QYS94233.1                        ........................................................MKQ
[Trichoderma_harzianum_CBS_226.95]_XP_024777038.1         ........................................................MKQ
[Trichoderma_harzianum]_PKK42226.1                        ........................................................MKQ
[Trichoderma_lentiforme]_KAF3077685.1_                    ........................................................MKQ
[Trichoderma_harzianum]_PNP59423.1                        ........................................................MTT
[Trichoderma_virens_FT-333]_UKZ73321.1                    ........................................................MKQ
[Trichoderma_virens_Gv29-8]_XP_013960763.1                ........................................................MKQ
[Trichoderma_asperellum_CBS_433.97]_XP_024756506.         ........................................................MRK
[Trichoderma_atroviride_IMI_206040]_XP_013943551.         ........................................................MRK
[Trichoderma_gamsii]_PNP38305.1                           ........................................................MRK
[Trichoderma_gamsii]_XP_018663207.1                       ........................................................MRK
[Trichoderma_arundinaceum]_RFU75476.1                     ........................................................MTK
[Trichoderma_gracile]_KAH0498535.1                        ........................................................MKQ
[Trichoderma_reesei_QM6a]_XP_006968245.1                  ........................................................MKQ
[Trichoderma_citrinoviride]_XP_024753310.1                ........................................................MKQ
[Trichoderma_longibrachiatum_ATCC_18648]_PTB78011         ........................................................MKQ
[Hirsutella_minnesotensis_3608]_KJZ78693.1                ........................................................MST
[Hirsutella_rhossiliensis]_XP_044725020.1                 ........................................................MT.
[Ophiocordyceps_sinensis_CO18]_EQK99704.1                 ........................................................MTN
[Tolypocladium_capitatum]_PNY29418.1                      ........................................................MSR
[Tolypocladium_ophioglossoides_CBS_100239]_KND888         ........................................................MSR
[Tolypocladium_paradoxum]_POR38551.1                      ........................................................MSR
[Drechmeria_coniospora]_ODA83100.1                        ........................................................MRN
[Drechmeria_coniospora]_XP_040657415.1                    MTSVA................RTRIRPQPAHFHFLNVLYRPVLTAPWTHWQPGSRVMRN
[Ophiocordyceps_unilateralis]_PFH57814.1                  ........................................................MSR
[Fusarium_sp._AF-6]_RSL54391.1                            ........................................................MSR
[Fusarium_kuroshium]_RMJ16314.1                           ........................................................MSR
[Fusarium_sp._AF-8]_RSL70798.1                            ........................................................MSR
[Fusarium_ambrosium]_RSM11997.1                           ........................................................MSR
[Fusarium_floridanum]_RSL80024.1                          ........................................................MSR
[Fusarium_vanettenii_77-13-4]_XP_003050716.1              ........................................................MSR
[Fusarium_solani]_XP_046134414.1                          ........................................................MSK
[Fusarium_albosuccineum]_KAF4468415.1                     MRRQTIFIHNI.....................SFGPCD...............SRSMSR
[Fusarium_decemcellulare]_KAF5011412.1                    MRRQTIFINNI.....................SFGPCD...............SRSMSR
[Fusarium_sarcochroum]_KAF4964785.1                       ........................................................MNR
[Fusarium_avenaceum]_KAH6968652.1                         ........................................................MNR
[Fusarium_avenaceum]_KIL96274.1                           ........................................................MNR
[Fusarium_avenaceum]_KAG5656265.1                         MVQQIACTRTVTSPISDFVPR.CDIPQPQPSIANCIHAT..............RSSMNR
[Fusarium_tricinctum]_KAH7262197.1                        ........................................................MNR
[Fusarium_heterosporum]_KAF5663380.1                      MR.......TVTSPMYRTSSHATTVLQPQPSSAHCTHAI..............QSSMNS
[Fusarium_sp._MPI-SDFR-AT-0072]_KAH7164412.1              MHSR....RDVTSS.RHSFHLTTRTLPTPTLLSHCAHAT..............QQSMNR
[Fusarium_oxysporum_f._sp._rapae]_KAG7414252.1            ........................................................MNR
[Fusarium_mangiferae]_XP_041681833.1                      ........................................................MNR
[Fusarium_proliferatum]_CVL11341.1                        ........................................................MNR
[Fusarium_proliferatum_ET1]_XP_031083673.1                ........................................................MNR
[Fusarium_fujikuroi]_SCN78580.1                           ........................................................MNR
[Fusarium_fujikuroi_IMI_58289]_XP_023430709.1             ........................................................MNR
[Fusarium_sp._NRRL_25303]_KAF5626228.1                    ........................................................MNR
[Fusarium_xylarioides]_KAG5746670.1                       ........................................................MNR
[Fusarium_xylarioides]_KAG5788219.1                       ........................................................MNR
[Fusarium_pseudocircinatum]_KAF5575212.1                  ...........................................................
[Fusarium_pseudoanthophilum]_KAF5575021.1                 ........................................................MNR
[Fusarium_verticillioides]_RBQ71412.1                     ........................................................MNR
[Fusarium_verticillioides_7600]_XP_018746479.1            ........................................................MNR
[Fusarium_oxysporum_NRRL_32931]_XP_031039788.1            MHSR....RDVISS.LQSFYLTIRTLPTPTLLSHCAHAT..............QQSMNK
[Fusarium_oxysporum_f._sp._lycopersici_4287]_XP_0         ........................................................MNK
[Fusarium_oxysporum_f._sp._cepae]_RKK20771.1              ........................................................MNK
[Fusarium_redolens]_XP_046051341.1                        ........................................................MNR
[Fusarium_austroafricanum]_KAF4458007.1                   MT.............LDFLHSNATTLNNNASMEFCAYAT..............RQFMNT
[Fusarium_flagelliforme]_XP_045986929.1                   ........................................................MNR
[Fusarium_sp._FIESC_RH6]_KAI1056250.1                     ......................................M..............ARSMSR
[Fusarium_sporotrichioides]_RGP72309.1                    .........................MSPQHSIVSSTHAI..............RKFMNR
[Fusarium_poae]_XP_044713592.1                            ........................................................MNR
[Fusarium_venenatum]_XP_025590637.1                       ........................................................MNR
[Fusarium_pseudograminearum]_KAF0641638.1                 .........................MSPQHSIVSSTHAI..............QKSMNR
[Fusarium_graminearum]_CAG1993854.1                       .........................MSPQHSIVSSTHAI..............QKSMNI
[Fusarium_graminearum]_PCD31638.1                         .........................MSPQHSIVSSTHAI..............QKSMNI
[Fusarium_graminearum]_CZS78629.1                         ........................................................MNI
[Fusarium_graminearum]_CAF3606094.1                       .........................MSPQHSIVSSTHAI..............QKSMNR
[Fusarium_graminearum_PH-1]_XP_011318944.1                ........................................................MNR
[Fusarium_culmorum]_QPC68571.1                            .........................MSPQHSIVSSTHAI..............QKSMNR
[Fusarium_pseudograminearum]_QPC77441.1                   .........................MSPQHSIVSYTHAI..............QKSMNR
[Fusarium_longipes]_RGP79158.1                            .........................MSPQLPIASFTHAV..............QKSMNR
[Ilyonectria_destructans]_KAH7011941.1                    ........................................................MSR
[Ilyonectria_sp._MPI-CAGE-AT-0026]_KAH6985660.1           ........................................................MSR
[Ilyonectria_robusta]_XP_046104990.1                      ........................................................MSR
[Dactylonectria_estremocensis]_KAH7149692.1               ........................................................MSR
[Dactylonectria_macrodidyma]_KAH7148923.1                 ........................................................MSR
[Thelonectria_olida]_KAH6899778.1                         ........................................................MTQ
[Stachybotrys_chartarum_IBT_7711]_KEY69997.1              ........................................................MNK
[Stachybotrys_chartarum_IBT_40293]_KFA50745.1             ........................................................MNK
[Stachybotrys_chlorohalonata_IBT_40285]_KFA62815.         ........................................................MNK
[Purpureocillium_lilacinum]_XP_018174914.1                M.......................................................ITR
[Clonostachys_solani]_CAH0059244.1                        ........................................................MSS
[Clonostachys_rosea]_VUC36940.1                           ........................................................MSS
[Clonostachys_rhizophaga]_CAH0026503.1                    ........................................................MKK
[Emericellopsis_atlantica]_XP_046114137.1                 ........................................................MRP
[Acremonium_chrysogenum_ATCC_11550]_KFH46845.1            ........................................................MST
[Pochonia_chlamydosporia_170]_XP_018148143.1              ........................................................MSK
[Epichloe_festucae_Fl1]_QPG98063.1                        ........................................................MRP
[Colletotrichum_incanum]_KZL82863.1                       MNSAC...................................................LTR
[Colletotrichum_simmondsii]_KXH39849.1                    MNNAC...................................................LSR
[Colletotrichum_sojae]_KAF6819546.1                       MNTAC...................................................LAR
```

```
                                                          η1                                        α1
[Trichoderma_harzianum]_ThaL                                                                        
                                                                   10          20
[Trichoderma_harzianum]_ThaL                          ..........WPH.WRGTRLPR............SLLSLQ.............PST.LRV
[Trichoderma_harzianum]_KKO99473.1                    ..........WPH.WRGTRLPR............SLLSLQ.............PST.SRV
[Trichoderma_guizhouense]_OPB43634.1                  ..........WPH.WRGTRLPR............SLLSLQ.............PST.SRV
[Trichoderma_simmonsii]_QYS94233.1                    ..........WPH.WRGTRLPR............SLLSLQ.............PSH.SRV
[Trichoderma_harzianum_CBS_226.95]_XP_024777038.1     ..........WPH.WRGTRLPR............SLLSLQ.............PSA.SRV
[Trichoderma_harzianum]_PKK42226.1                    ..........WPH.WRGTRLPR............SLLSLQ.............PSA.SRV
[Trichoderma_lentiforme]_KAF3077685.1_                ..........WPH.WRGTRLPR............SLLKLQ.............PSP.SRV
[Trichoderma_harzianum]_PNP59423.1                    ...................LLS..........................................
[Trichoderma_virens_FT-333]_UKZ73321.1                ..........WRH.WRSSRLRA............SLLNLR.............SSP.SRV
[Trichoderma_virens_Gv29-8]_XP_013960763.1            ..........WRH.WRRSRLRV............SLPNLR.............SSP.SRV
[Trichoderma_asperellum_CBS_433.97]_XP_024756506.     ..........WLL.HRGPRLSR............PPPNLR.............SSS.HQL
[Trichoderma_atroviride_IMI_206040]_XP_013943551.     ..........WLLHHRGPRLSR............PLPNLR.............SIPWHHF
[Trichoderma_gamsii]_PNP38305.1                       ..........WLLHYRGPRLSP...........LPPPNLR.............PS..HHF
[Trichoderma_gamsii]_XP_018663207.1                   ..........WLLHYRGPRLSP...........LPPPNLR.............PS..HHF
[Trichoderma_arundinaceum]_RFU75476.1                 ..........WHT.YRGPRLSR............SLPNLR.............PSASYRF
[Trichoderma_gracile]_KAH0498535.1                    ..........WPR.HHGPRLSR............SLPNLR.............RSR.ART
[Trichoderma_reesei_QM6a]_XP_006968245.1              ..........WPR.HHGPRLSR............SLPNLR.............RSR.ART
[Trichoderma_citrinoviride]_XP_024753310.1            ..........WPR.HHGPRLSR............SLLNLR.............PSRPRRA
[Trichoderma_longibrachiatum_ATCC_18648]_PTB78011     ..........WPR.QYGPRLTR............SPPTLR.............RSR.ARP
[Hirsutella_minnesotensis_3608]_KJZ78693.1            ..........CSPRAPAVSIHR.AAAAAARQ.SCRRPSCR.............PLLV.RRA
[Hirsutella_rhossiliensis]_XP_044725020.1             .......................................................TRA
[Ophiocordyceps_sinensis_CO18]_EQK99704.1             VGNSLAS...VHLRIPCKAICQ...RRMHSKTTLLRSACR.............RPT.HRA
[Tolypocladium_capitatum]_PNY29418.1                  ..........WAPPHNILTLAG...LRPQCR.HLPRPSCR..................RRS
[Tolypocladium_ophioglossoides_CBS_100239]_KND888     ..........WLPRYSISTLAG...RQPQCQ.HLPHLSCR...................RR
[Tolypocladium_paradoxum]_POR38551.1                  ..........WLPRCNTPILAG...RRPRRQ.HLPRPSCR..................RRS
[Drechmeria_coniospora]_ODA83100.1                    ........IPWLTRYYSPPSLAAGRLRPH.....STPACR.............LRH.GRT
[Drechmeria_coniospora]_XP_040657415.1                ........IPWLTRYYSPPSLAAGRLRPH.....STPACR.............LRH.GRT
[Ophiocordyceps_unilateralis]_PFH57814.1              .....................KCIIGSCLFARLPCASWP.............SLSN.NRP
[Fusarium_sp._AF-6]_RSL54391.1                        ..........SAPLRRALILAS............PRPSLS.....IQW....LSIG.SRG
[Fusarium_kuroshium]_RMJ16314.1                       ..........SAPLRRALILAS............PRPSSS.....IQW....LPIG.SRG
[Fusarium_sp._AF-8]_RSL70798.1                        ..........SAPLRRALILAS............PRPSSS.....TQW....LPIG.SRG
[Fusarium_ambrosium]_RSM11997.1                       ..........SAPLRRALILAS............PRPSSS.....IQW....LPIG.SRG
[Fusarium_floridanum]_RSL80024.1                      ..........SAPLRRALILAS............PRPSSS.....TQW....LSIG.SRG
[Fusarium_vanettenii_77-13-4]_XP_003050716.1          ..........SALLRRALILAS............PRPSSS.....IQW....LPIV.SRG
[Fusarium_solani]_XP_046134414.1                      ..........SALLRRALTLAS............PRLSSS.....TQW....LPIV.SRG
[Fusarium_albosuccineum]_KAF4468415.1                 ..........STLWRRTPVLAL............KNPPCR.....AQW....HPIP.RQA
[Fusarium_decemcellulare]_KAF5011412.1                ..........STLWRRTPVLAL............KNPPCR.....AQW....HPIP.RQA
[Fusarium_sarcochroum]_KAF4964785.1                   ..........SIIWRRAPTSAL............TKEPCR.....AQW....SPIA.RRS
[Fusarium_avenaceum]_KAH6968652.1                     ..........STIWRKAPAFPL............ARHTCR.....VQW....HHVP.RRN
[Fusarium_avenaceum]_KIL96274.1                       ..........STIWRKAPAFPL............ARHTCR.....VQW....HHVP.RRN
[Fusarium_avenaceum]_KAG5656265.1                     ..........STIWRKAPAFPL............ARHTCR.....AQW....HHVP.RRN
[Fusarium_tricinctum]_KAH7262197.1                    ..........SKIWRKAPAFPR............ARHTCH.....AQW....HPVP.RRN
[Fusarium_heterosporum]_KAF5663380.1                  ..........LIIWRKAPAPPL............ARHSCR.....AQW....HTVS.RRN
[Fusarium_sp._MPI-SDFR-AT-0072]_KAH7164412.1          ..........SAIWRNAPASPL............FRQLYR.....AQC.........HRS
[Fusarium_oxysporum_f._sp._rapae]_KAG7414252.1        ..........SAIWRNAPASPL............FRQLYR.....AQC.........HRS
[Fusarium_mangiferae]_XP_041681833.1                  ..........SVIWRNAPDLLL............SRQLYR.....AQW.........HRS
[Fusarium_proliferatum]_CVL11341.1                    ..........SVIWRNAPDLLL............SRQLYR.....AQW.........HRS
[Fusarium_proliferatum_ET1]_XP_031083673.1            ..........SVIWRNAPDLLL............SRQLYR.....AQW.........HRS
[Fusarium_fujikuroi]_SCN78580.1                       ..........SVIWRSAPDLLL............SRQLYR.....AQW.........HRS
[Fusarium_fujikuroi_IMI_58289]_XP_023430709.1         ..........SVIWRNAPDLLL............SRQLYR.....AQW.........HRS
[Fusarium_sp._NRRL_25303]_KAF5626228.1                ..........SVIWRNAPDLLL............SRQLYR.....AQW.........HRS
[Fusarium_xylarioides]_KAG5746670.1                   ..........SVIWRNAPTLPL............SQQLYR.....AQC.........HRK
[Fusarium_xylarioides]_KAG5788219.1                   ..........SVIWRNAPTLPL............SQQLYR.....AQC.........HRK
[Fusarium_pseudocircinatum]_KAF5575212.1              ..............MWRSAPALPL............SRQLYG.....ARC.........HRS
[Fusarium_pseudoanthophilum]_KAF5575021.1             ..........SVIWRSAPALPL............SRQLYG.....AQC.........HRS
[Fusarium_verticillioides]_RBQ71412.1                 ..........SVIWRSAPALPL............SRQLYG.....VQC.........HRS
[Fusarium_verticillioides_7600]_XP_018746479.1        ..........SVIWRSAPALPL............SRQLYG.....AQC.........HRS
[Fusarium_oxysporum_NRRL_32931]_XP_031039788.1        ..........SAIWRNAPASPL............SRQLYR.....AQC.........HRS
[Fusarium_oxysporum_f._sp._lycopersici_4287]_XP_0     ..........SAIWRNAPASPL............SRQLYR.....AQC.........HRS
[Fusarium_oxysporum_f._sp._cepae]_RKK20771.1          ..........SAIWRNAPASPL............SRQLYR.....AQC.........HRS
[Fusarium_redolens]_XP_046051341.1                    ..........SIIRRNAPTSPL............SRQLYR.....AQC.........HRS
[Fusarium_austroafricanum]_KAF4458007.1               ..........SRTWRKAPASAL............GRLLYH.....AQWH...HPVS.RRT
[Fusarium_flagelliforme]_XP_045986929.1               ..........STIWRKAPALSL............IKYPYR.....KQP....FLLQ.CRG
[Fusarium_sp._FIESC_RH6]_KAI1056250.1                 ..........STIWRKAPALGL............VRYPYH.....KQP....FLLQ.CRS
[Fusarium_sporotrichioides]_RGP72309.1                ..........STIWRKAPAPTL............VKYSYR.....KQP....FITQ.CRS
[Fusarium_poae]_XP_044713592.1                        ..........STIWRNAPASTL............VRYTYR.....KQR....PIVQ.CRS
[Fusarium_venenatum]_XP_025590637.1                   ..........STIWRKAPASTL............VRYACR.....KQH....PIVQ.CRS
[Fusarium_pseudograminearum]_KAF0641638.1             ..........PIIWRNAPAPTL............VRYTYR.....KQQ....PIVQ.CRS
[Fusarium_graminearum]_CAG1993854.1                   ..........SIIWRKAPVPTL............VKYPYR.....KQR....PIVQ.CRS
[Fusarium_graminearum]_PCD31638.1                     ..........SIIWRKAPVPTL............VKYPYR.....KQR....PMVQ.CRS
[Fusarium_graminearum]_CZS78629.1                     ..........SIIWRKAPVPTL............VKYPYR.....KQR....PMVQ.CRS
[Fusarium_graminearum]_CAF3606094.1                   ..........SIIWRKAPVPTL............VKYPYR.....KQR....PIVQ.CRS
[Fusarium_graminearum_PH-1]_XP_011318944.1            ..........SIIWRKAPVPTL............VKYPYR.....KQR....PIVQ.CRS
[Fusarium_culmorum]_QPC68571.1                        ..........SIIWRKAPVPTL............VKYPYR.....KQR....PIVQ.CRS
[Fusarium_pseudograminearum]_QPC77441.1               ..........SIIWRNAPAPTL............VRYTYR.....KQQ....PIVQ.CRS
[Fusarium_longipes]_RGP79158.1                        ..........SIIWRKAPVLTP............PRYPYC.....KQR....PIVQ.SRS
[Ilyonectria_destructans]_KAH7011941.1                ..........WIVQCKPPAIAV............TRPTTYLGVTRAQCLL..RDGH.SRS
[Ilyonectria_sp._MPI-CAGE-AT-0026]_KAH6985660.1       ..........WAVRCKPSAIAV............TSPTTYLGVTRAQCLL..RDRH.SRS
[Ilyonectria_robusta]_XP_046104990.1                  ..........WIVQCKPSAIAV............TNPTTYLGVTRAQCLL..RDRH.SRS
[Dactylonectria_estremocensis]_KAH7149692.1           ..........RLIRGSIPTIAA............ARPAARLSVTRAQRFQ..NGWP.ARS
[Dactylonectria_macrodidyma]_KAH7148923.1             ..........RLIRGNVPTIVV............ARPAARLSVTCAQRSP..NGWP.ART
[Thelonectria_olida]_KAH6899778.1                     ..........WLARCRQPVITA............SGLPSTTITRPVQWLG..KTQN.ARS
[Stachybotrys_chartarum_IBT_7711]_KEY69997.1          ..........WLIRSAKSVLST....AP......SCLVSR......RCPP..PLPS.RRA
[Stachybotrys_chartarum_IBT_40293]_KFA50745.1         ..........WPIRSAKSVLST....AP......SCLVSR......RCPP..PLPS.RRA
[Stachybotrys_chlorohalonata_IBT_40285]_KFA62815.     ..........WPIRSAKSVLST....AP......SCLMSR......YCPP..PLPL.HRA
[Purpureocillium_lilacinum]_XP_018174914.1            ..........WAPRHSTTTTTT.AAAAAVTL.AVRRPAIR.....PHCRPSSPLPRRRL
[Clonostachys_solani]_CAH0059244.1                    RLAHHGVRIGFNPRITTTTLKA............NFLLQR..................QQH
[Clonostachys_rosea]_VUC36940.1                       RLAHHGVRIGFNPRITTTALNP............NFLLQS..................QRH
[Clonostachys_rhizophaga]_CAH0026503.1                ..................................QCVLIR.......................
[Emericellopsis_atlantica]_XP_046114137.1             .....CRR..FHSARSLTGLAQ............ARHRVR.............HTPQ.RRS
[Acremonium_chrysogenum_ATCC_11550]_KFH46845.1        ..........WLLRRNAPIRRL...NGPASKPRLPPAATR...............QQP.CRT
[Pochonia_chlamydosporia_170]_XP_018148143.1          ..........CLP.RSVPLASL............RIHHAR.............HPLS.HRH
[Epichloe_festucae_Fl1]_QPG98063.1                    ..........WLPRRATPLCHE............AVLSTRL....IQCCP..........
[Colletotrichum_incanum]_KZL82863.1                   ..........CRPRAPSPGLIR....APT....ASPRLLR.........PLASPGS.SRC
[Colletotrichum_simmondsii]_KXH39849.1                ..........CRPCAPSSRLIC....APT....ASHRLLR.........PLAAR.D.SRP
[Colletotrichum_sojae]_KAF6819546.1                   FSCSSSSR..HLRRRAIPATFL...SVPTRA.PAASASLR.........PLPCRAS.RRC
```

[Trichoderma_harzianum]_ThaL

```
                                                        TT
                                               30                  40          50
[Trichoderma_harzianum]_ThaL                          FSTAKPL......RHDPRIRDLGRLIHDDYATIRETY......................AT
[Trichoderma_harzianum]_KKO99473.1                    FSTAKPL......RHDPRIRDLGRLIHDDYATIRETY......................AT
[Trichoderma_guizhouense]_OPB43634.1                  FSTAKPL......RHDPRIRDLGRLIHDDYATIRETY......................AT
[Trichoderma_simmonsii]_QYS94233.1                    FSTTKPL......RHDPRIRDLGRLIHDDYATIRETY......................AT
[Trichoderma_harzianum_CBS_226.95]_XP_024777038.1     FSTTKPL......RHDPRIRDLGRLIHDDYATIRETY......................AT
[Trichoderma_harzianum]_PKK42226.1                    FSTTNPL......RHDPRIRDLGRLIHDDYATIRETY......................AT
[Trichoderma_lentiforme]_KAF3077685.1_                FSTAKPLL...SSSHDPRIRDLGRLIHDDYATIRETY......................AT
[Trichoderma_harzianum]_PNP59423.1                    .....................................................................AR
[Trichoderma_virens_FT-333]_UKZ73321.1                LSTARPLL...SSGHDPRIRDLGRRIHDDYATIRETY......................AT
[Trichoderma_virens_Gv29-8]_XP_013960763.1            LSTARPLL...SSGHDPRIRDLGRRIHDDYATIRETY......................AT
[Trichoderma_asperellum_CBS_433.97]_XP_024756506.     FSTAKPHL...KSSHDPRIRDFGRRIHDEYATIRENY......................AT
[Trichoderma_atroviride_IMI_206040]_XP_013943551.     FSTAKAHL...KDSHDPRIRDFGRRIHDDYATIRENY......................AT
[Trichoderma_gamsii]_PNP38305.1                       FSTAKAYL...KDSNDPRIRDFGRRIHDDYATIRENYGMIESSARLQKEVGTFTDTTTAT
[Trichoderma_gamsii]_XP_018663207.1                   FSTAKAYL...KDSSDPRIRDVGRRIHDDYATIRENYGMIESSARLQKEVGTFTDTTTAT
[Trichoderma_arundinaceum]_RFU75476.1                 FSAAKPVL...RPSNDPRIRDLGRRILDDYAIIRDNY......................AT
[Trichoderma_gracile]_KAH0498535.1                    FSTGKPLL...DDSHDPRIRDLGRQILDDYAIIRENY......................AT
[Trichoderma_reesei_QM6a]_XP_006968245.1              FSAAKPLL..HESSHDPRIRDLGRQILDDYAMIRENY......................AT
[Trichoderma_citrinoviride]_XP_024753310.1            FSAGRPFL.EEESSHDPRIRDLGRRILDDYAVIRENY......................AT
[Trichoderma_longibrachiatum_ATCC_18648]_PTB78011     FSTGKPFL..DESSHDPRIRDLGRQILDDYAVIREKY......................AT
[Hirsutella_minnesotensis_3608]_KJZ78693.1            FATDAPL....LRDGDARLRHLGREIADEYATIRENY......................AT
[Hirsutella_rhossiliensis]_XP_044725020.1             LTAGRALR...RDDSDARIRELGREIAHDYATIRESY......................AT
[Ophiocordyceps_sinensis_CO18]_EQK99704.1             STPARPP....RRHSHGRIRDLGPEIARDYAAIRDSY......................AT
[Tolypocladium_capitatum]_PNY29418.1                  LTTSRSH....RRDSDPRIRHLGRRIADEYATIRENYGLRPHGMLTNASSETARLTTAAT
[Tolypocladium_ophioglossoides_CBS_100239]_KND888     LTTSLSH....RRNSDPRIRDLGRQIADEYAAIREAY......................AT
[Tolypocladium_paradoxum]_POR38551.1                  LTTSRPR....RRDSDARMRDLGRQIADEYASIRESY......................AT
[Drechmeria_coniospora]_ODA83100.1                    LTSSPFL....HHSSDPRVRNLGRQISDEYASVRDVY......................LT
[Drechmeria_coniospora]_XP_040657415.1                LTSSPFL....HRSSDPRVRNLGREISDEYASVRDVY......................LT
[Ophiocordyceps_unilateralis]_PFH57814.1              LTTGRAL....RDDNDARMRHPGRPLAQEFADIREKY......................AT
[Fusarium_sp._AF-6]_RSL54391.1                        FSSGRYL....RRSEDPRIKALGREISDDYATIREKY......................KT
[Fusarium_kuroshium]_RMJ16314.1                       FSSGRYL....RRSEDPRIKALGREISDDYATIREKY......................KT
[Fusarium_sp._AF-8]_RSL70798.1                        FSSGRYL....RRSEDPRIKALGREISDDYATIREKY......................KT
[Fusarium_ambrosium]_RSM11997.1                       FSSGRYL....WRSEDPRIKALGREISDDYATIREKY......................KT
[Fusarium_floridanum]_RSL80024.1                      FSSGWYL....RRSEDPRIKALGREISDDYATIREKY......................KT
[Fusarium_vanettenii_77-13-4]_XP_003050716.1          FSSGRYL....RRSEDPRIKALGREISDDYATIREKY......................KT
[Fusarium_solani]_XP_046134414.1                      FSSGRCL....RRSEDPRIKALGREISDDYATIREKY......................KT
[Fusarium_albosuccineum]_KAF4468415.1                 FSSGRHL....RRDEDPRIKALGREISDDYATIREKY......................ST
[Fusarium_decemcellulare]_KAF5011412.1                FSSGRHL....RRDEDPRIKALGREISDDYATIREKY......................ST
[Fusarium_sarcochroum]_KAF4964785.1                   FTCSSCL....RREHDPRIKALGRQISDDYAIIREKY......................ET
[Fusarium_avenaceum]_KAH6968652.1                     FTCSSFL....HREHDPRIKALDREISDDYAIIREKY......................DT
[Fusarium_avenaceum]_KIL96274.1                       FTCSSFL....HREHDPRIKALDREISDDYAIIREKY......................DT
[Fusarium_avenaceum]_KAG5656265.1                     FTCSSFL....HREHDPRIKALDREISDDYAIIREKY......................DT
[Fusarium_tricinctum]_KAH7262197.1                    FTCSSFL....HREDDPRIKALDREISDDYAMIREKY......................DT
[Fusarium_heterosporum]_KAF5663380.1                  FTRSSFL....RREHDPRIKALGREISDDYAIIREKY......................ET
[Fusarium_sp._MPI-SDFR-AT-0072]_KAH7164412.1          FACSTYL....RREHDPRIKALGREISDDYAIIREKY......................ET
[Fusarium_oxysporum_f._sp._rapae]_KAG7414252.1        FACSTYL....RREHDPRIKALGREISDDYAIIREKY......................ET
[Fusarium_mangiferae]_XP_041681833.1                  FACSAYL....TREHDPRIKALGREISDDYATIREKY......................ET
[Fusarium_proliferatum]_CVL11341.1                    FACSAYL....TREHDPRIKALGREISDDYAIIREKY......................ET
[Fusarium_proliferatum_ET1]_XP_031083673.1            FACSAYL....TREHDPRIKALGREISDDYAIIREKY......................ET
[Fusarium_fujikuroi]_SCN78580.1                       FACSAYL....TREHDPRIKALGREISDDYAIIREKY......................ET
[Fusarium_fujikuroi_IMI_58289]_XP_023430709.1         FACSAYL....TREHDPRIKALGREISDDYAIIREKY......................ET
[Fusarium_sp._NRRL_25303]_KAF5626228.1                FACSAYL....TREHDPRIKALGREISDDYATIREKY......................ET
[Fusarium_xylarioides]_KAG5746670.1                   FACSTYL....GREHDPRIKALGREISDDYAIIREKY......................ET
[Fusarium_xylarioides]_KAG5788219.1                   FACSTYL....GREHDPRIKALGREISDDYAIIREKY......................ET
[Fusarium_pseudocircinatum]_KAF5575212.1              FACSTYL....AREHDPRIKALGREISDDYAIIREKY......................ET
[Fusarium_pseudoanthophilum]_KAF5575021.1             FACSAYL....AREHDPRIKALGREISDDYAIIREKY......................ET
[Fusarium_verticillioides]_RBQ71412.1                 FACSTYL....AREHDPRIKALGREISDDYAIIREKY......................ET
[Fusarium_verticillioides_7600]_XP_018746479.1        FACSTYL....AREHDPRIKALGREISDDYAIIREKY......................ET
[Fusarium_oxysporum_NRRL_32931]_XP_031039788.1        FACSTYL....GREHDPRIKALGREISDDYATIREKY......................ET
[Fusarium_oxysporum_f._sp._lycopersici_4287]_XP_0     FACSTYL....GREHDPRIKALGREISDDYAIIREKY......................ET
[Fusarium_oxysporum_f._sp._cepae]_RKK20771.1          FACSTYL....GREHDPRIRALGREISDDYAIIREKY......................ET
[Fusarium_redolens]_XP_046051341.1                    FACSSYL....GREHDPRIKALGREISDDYAIIREKYGA................AFHPTI
[Fusarium_austroafricanum]_KAF4458007.1               FTRSSYQ....GREHDPRIRALGREISGDYAIIREKY......................EI
[Fusarium_flagelliforme]_XP_045986929.1               FTHSHCF....GREHDPRIKALGREISDDYAIIREKY......................ET
[Fusarium_sp._FIESC_RH6]_KAI1056250.1                 FTHSHCF....GREHDPRIKALGREISDDYAIIREKY......................ET
[Fusarium_sporotrichioides]_RGP72309.1                FTYSRCV....ARDYDPRIKAVGRAISDDYALIREKY......................DT
[Fusarium_poae]_XP_044713592.1                        FTYSRYV....GREYDPRIRAVGRAISDDYALIREKY......................DT
[Fusarium_venenatum]_XP_025590637.1                   FTYSRYV....GREYDPRIKAVGRAISDDYALIREKY......................DT
[Fusarium_pseudograminearum]_KAF0641638.1             FTYSRCV....GREFDPRIKAVGRAISDDYALIREKY......................DT
[Fusarium_graminearum]_CAG1993854.1                   FTYSRCV....GREFDPRIKAVGRAISDDYALIREKY......................DT
[Fusarium_graminearum]_PCD31638.1                     FTYSRCV....GREFDPRIKAVGRAISDDYALIREKY......................DT
[Fusarium_graminearum]_CZS78629.1                     FTYSRCV....GREFDPRIKAVGRAISDDYALIREKY......................DT
[Fusarium_graminearum]_CAF3606094.1                   FTYSRCV....GREFDPRIKAVGRAISDDYALIREKY......................DT
[Fusarium_graminearum_PH-1]_XP_011318944.1            FTYSRCV....GREFDPRIKAVGRAISDDYALIREKY......................DT
[Fusarium_culmorum]_QPC68571.1                        FTYSRCV....GREFDPRIKAVGRAISDDYALIREKY......................DT
[Fusarium_pseudograminearum]_QPC77441.1               FTYSRCV....GREFDPRIKAVGRAISDDYALIREKYGRFSKSWFLAWGNLTD.NHATDT
[Fusarium_longipes]_RGP79158.1                        FAYSRCV....GREYDPRIKAVGRAISNDYALIREKY......................DT
[Ilyonectria_destructans]_KAH7011941.1                LTTGRPL....RRDGDPRIRPLGRQISDDYATIRDTY......................DT
[Ilyonectria_sp._MPI-CAGE-AT-0026]_KAH6985660.1       LTTGRPL....RRDGDPRIRPLGRQISDDYATIRDTY......................DT
[Ilyonectria_robusta]_XP_046104990.1                  LTTGRPL....RRDGDPRIRPLGRQISDDYATIRDTY......................DT
[Dactylonectria_estremocensis]_KAH7149692.1           FSTGRPW....RQEGDPRIRPLGKQISDDYATIRDSY......................ET
[Dactylonectria_macrodidyma]_KAH7148923.1             LSTGHTL....RQERDPRIRPLGKQISDDYATIRDSY......................ET
[Thelonectria_olida]_KAH6899778.1                     FTAGRPL....RQDSDPRVRELGREISDDFATIRDTY......................ET
[Stachybotrys_chartarum_IBT_7711]_KEY69997.1          FASERP.....RDNSDPRIRDLGRQISDDYAALRDSY......................AS
[Stachybotrys_chartarum_IBT_40293]_KFA50745.1         FASERP.....RDNSDPRIRDLGRQISDDYAALRDSY......................AS
[Stachybotrys_chlorohalonata_IBT_40285]_KFA62815.     FTSERP.....RDNSDPRIRDLGRQISDDYAALRDSY......................AS
[Purpureocillium_lilacinum]_XP_018174914.1            LSTSFPPRREERKAPDPRIRDLGRQIADDYATIRDTY......................AT
[Clonostachys_solani]_CAH0059244.1                    VGQRRWNS...SRDVDPRIKALGRTILDDYAAVREEY......................AT
[Clonostachys_rosea]_VUC36940.1                       VGQRRWNS...SRDVDPRIKALGRTILDDYAAVREEY......................AT
[Clonostachys_rhizophaga]_CAH0026503.1                .........EFGRDVDPRIKALGRTILDDYAAVREEYGIFQPHRYDSIRKHQQLTTNPAT
[Emericellopsis_atlantica]_XP_046114137.1             FASTALR....PQDHDPRIRELGREISDDYAAIRDSY......................QT
[Acremonium_chrysogenum_ATCC_11550]_KFH46845.1        LSSSTYAL...HRDGDPRIRDLGREISDDFATVRDTY......................AT
[Pochonia_chlamydosporia_170]_XP_018148143.1          ISSTSHN....QRSRDPRIRDVGRQIEDDYAAIREHY......................AT
[Epichloe_festucae_Fl1]_QPG98063.1                    LSQTACAR...TRAQDARVRDIGRKIKDDYAYIRDHY......................AA
[Colletotrichum_incanum]_KZL82863.1                   ISASPSQS..KHHDDDPRFRDLGKQISDEYAVVREHY......................ET
[Colletotrichum_simmondsii]_KXH39849.1                FSVSPLRP...KHDGDPRFRDLGKQISDEYAVVREHY......................DT
[Colletotrichum_sojae]_KAF6819546.1                   LSTTTPRP...RDDSDPRLRDLGRQISDDFAVVREHY......................DT
```

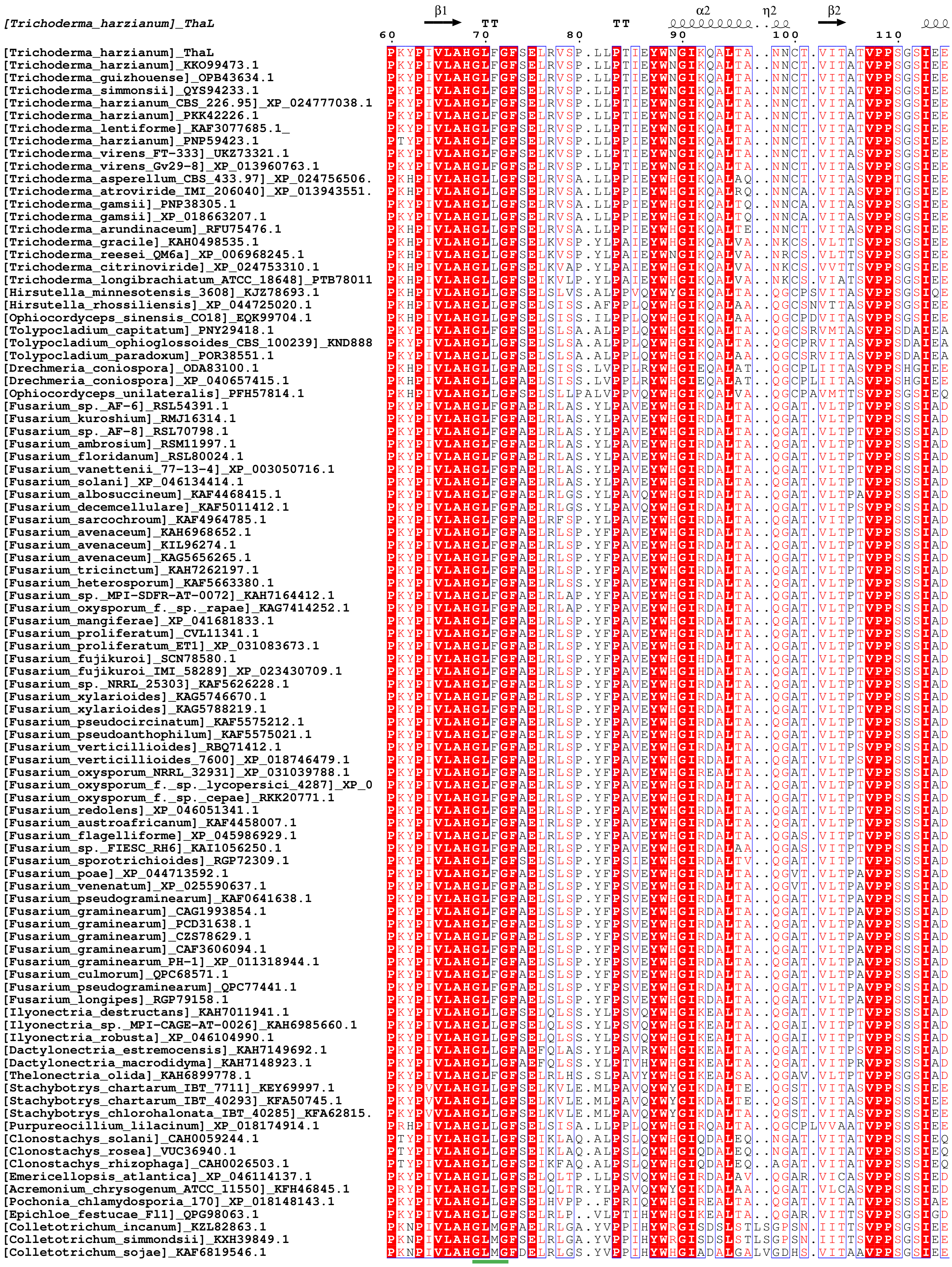

[Trichoderma_harzianum]_ThaL
β1
TT
TT
α2
η2
β2
60
70
80
90
100
110
[Trichoderma_harzianum]_ThaL
[Trichoderma_harzianum]_KKO99473.1
[Trichoderma_guizhouense]_OPB43634.1
[Trichoderma_simmonsii]_QYS94233.1
[Trichoderma_harzianum_CBS_226.95]_XP_024777038.1
[Trichoderma_harzianum]_PKK42226.1
[Trichoderma_lentiforme]_KAF3077685.1_
[Trichoderma_harzianum]_PNP59423.1
[Trichoderma_virens_FT-333]_UKZ73321.1
[Trichoderma_virens_Gv29-8]_XP_013960763.1
[Trichoderma_asperellum_CBS_433.97]_XP_024756506.
[Trichoderma_atroviride_IMI_206040]_XP_013943551.
[Trichoderma_gamsii]_PNP38305.1
[Trichoderma_gamsii]_XP_018663207.1
[Trichoderma_arundinaceum]_RFU75476.1
[Trichoderma_gracile]_KAH0498535.1
[Trichoderma_reesei_QM6a]_XP_006968245.1
[Trichoderma_citrinoviride]_XP_024753310.1
[Trichoderma_longibrachiatum_ATCC_18648]_PTB78011
[Hirsutella_minnesotensis_3608]_KJZ78693.1
[Hirsutella_rhossiliensis]_XP_044725020.1
[Ophiocordyceps_sinensis_CO18]_EQK99704.1
[Tolypocladium_capitatum]_PNY29418.1
[Tolypocladium_ophioglossoides_CBS_100239]_KND888
[Tolypocladium_paradoxum]_POR38551.1
[Drechmeria_coniospora]_ODA83100.1
[Drechmeria_coniospora]_XP_040657415.1
[Ophiocordyceps_unilateralis]_PFH57814.1
[Fusarium_sp._AF-6]_RSL54391.1
[Fusarium_kuroshium]_RMJ16314.1
[Fusarium_sp._AF-8]_RSL70798.1
[Fusarium_ambrosium]_RSM11997.1
[Fusarium_floridanum]_RSL80024.1
[Fusarium_vanettenii_77-13-4]_XP_003050716.1
[Fusarium_solani]_XP_046134414.1
[Fusarium_albosuccineum]_KAF4468415.1
[Fusarium_decemcellulare]_KAF5011412.1
[Fusarium_sarcochroum]_KAF4964785.1
[Fusarium_avenaceum]_KAH6968652.1
[Fusarium_avenaceum]_KIL96274.1
[Fusarium_avenaceum]_KAG5656265.1
[Fusarium_tricinctum]_KAH7262197.1
[Fusarium_heterosporum]_KAF5663380.1
[Fusarium_sp._MPI-SDFR-AT-0072]_KAH7164412.1
[Fusarium_oxysporum_f._sp._rapae]_KAG7414252.1
[Fusarium_mangiferae]_XP_041681833.1
[Fusarium_proliferatum]_CVL11341.1
[Fusarium_proliferatum_ET1]_XP_031083673.1
[Fusarium_fujikuroi]_SCN78580.1
[Fusarium_fujikuroi_IMI_58289]_XP_023430709.1
[Fusarium_sp._NRRL_25303]_KAF5626228.1
[Fusarium_xylarioides]_KAG5746670.1
[Fusarium_xylarioides]_KAG5788219.1
[Fusarium_pseudocircinatum]_KAF5575212.1
[Fusarium_pseudoanthophilum]_KAF5575021.1
[Fusarium_verticillioides]_RBQ71412.1
[Fusarium_verticillioides_7600]_XP_018746479.1
[Fusarium_oxysporum_NRRL_32931]_XP_031039788.1
[Fusarium_oxysporum_f._sp._lycopersici_4287]_XP_0
[Fusarium_oxysporum_f._sp._cepae]_RKK20771.1
[Fusarium_redolens]_XP_046051341.1
[Fusarium_austroafricanum]_KAF4458007.1
[Fusarium_flagelliforme]_XP_045986929.1
[Fusarium_sp._FIESC_RH6]_KAI1056250.1
[Fusarium_sporotrichioides]_RGP72309.1
[Fusarium_poae]_XP_044713592.1
[Fusarium_venenatum]_XP_025590637.1
[Fusarium_pseudograminearum]_KAF0641638.1
[Fusarium_graminearum]_CAG1993854.1
[Fusarium_graminearum]_PCD31638.1
[Fusarium_graminearum]_CZS78629.1
[Fusarium_graminearum]_CAF3606094.1
[Fusarium_graminearum_PH-1]_XP_011318944.1
[Fusarium_culmorum]_QPC68571.1
[Fusarium_pseudograminearum]_QPC77441.1
[Fusarium_longipes]_RGP79158.1
[Ilyonectria_destructans]_KAH7011941.1
[Ilyonectria_sp._MPI-CAGE-AT-0026]_KAH6985660.1
[Ilyonectria_robusta]_XP_046104990.1
[Dactylonectria_estremocensis]_KAH7149692.1
[Dactylonectria_macrodidyma]_KAH7148923.1
[Thelonectria_olida]_KAH6899778.1
[Stachybotrys_chartarum_IBT_7711]_KEY69997.1
[Stachybotrys_chartarum_IBT_40293]_KFA50745.1
[Stachybotrys_chlorohalonata_IBT_40285]_KFA62815.
[Purpureocillium_lilacinum]_XP_018174914.1
[Clonostachys_solani]_CAH0059244.1
[Clonostachys_rosea]_VUC36940.1
[Clonostachys_rhizophaga]_CAH0026503.1
[Emericellopsis_atlantica]_XP_046114137.1
[Acremonium_chrysogenum_ATCC_11550]_KFH46845.1
[Pochonia_chlamydosporia_170]_XP_018148143.1
[Epichloe_festucae_Fl1]_QPG98063.1
[Colletotrichum_incanum]_KZL82863.1
[Colletotrichum_simmondsii]_KXH39849.1
[Colletotrichum_sojae]_KAF6819546.1

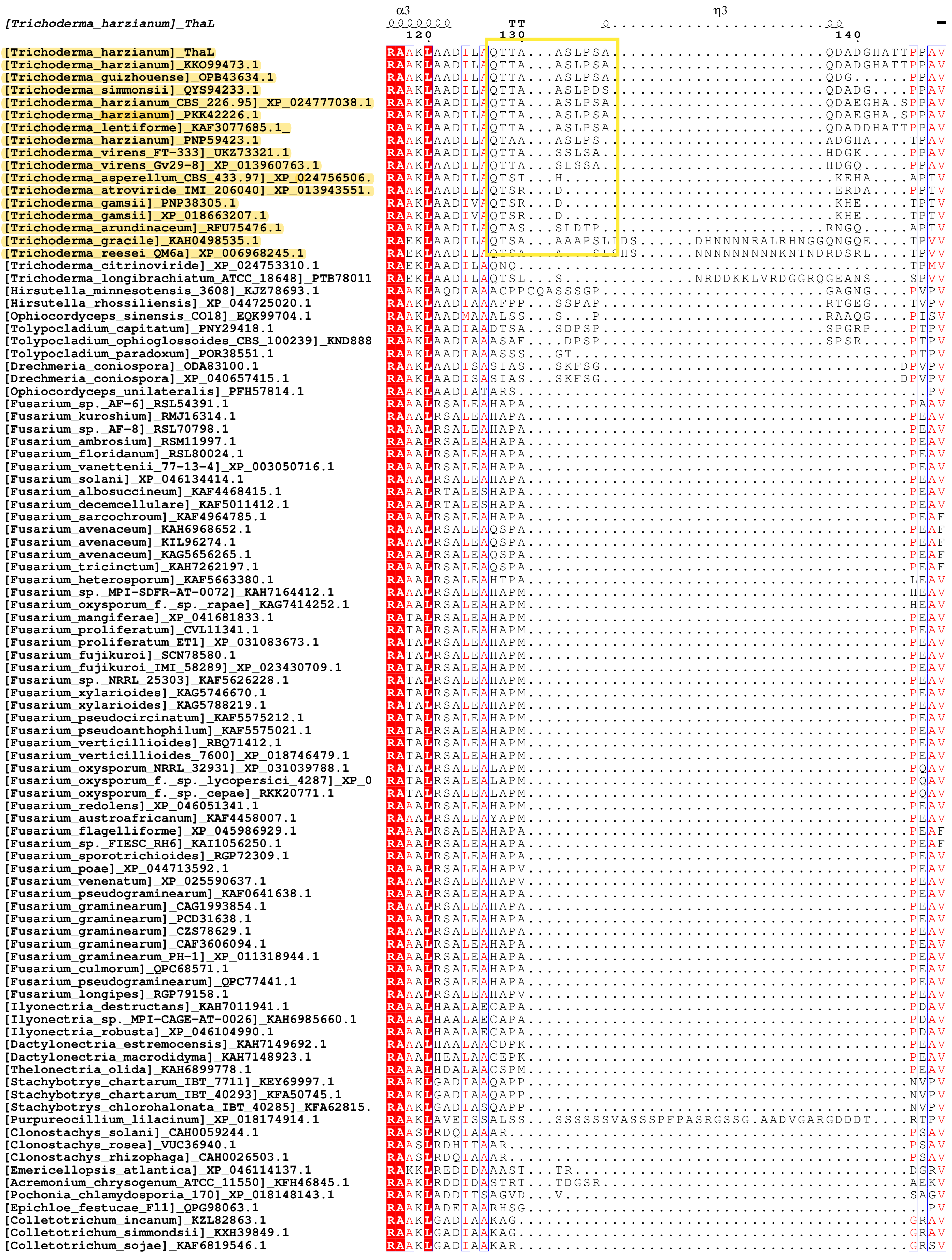

[Trichoderma_harzianum]_ThaL
α3
TT
η3
120
130
140
[Trichoderma_harzianum]_ThaL RAAKLAADILAQTTA...ASLPSA.......................QDADGHATTPPAV
[Trichoderma_harzianum]_KKO99473.1 RAAKLAADILAQTTA...ASLPSA.......................QDADGHATTPPAV
[Trichoderma_guizhouense]_OPB43634.1 RAAKLAADILAQTTA...ASLPSA.......................QDG......PPAV
[Trichoderma_simmonsii]_QYS94233.1 RAAKLAADILAQTTA...ASLPDS.......................QDADG....PPAV
[Trichoderma_harzianum_CBS_226.95]_XP_024777038.1 RAAKLAADILAQTTA...ASLPSA.......................QDAEGHA.SPPAV
[Trichoderma_harzianum]_PKK42226.1 RAAKLAADILAQTTA...ASLPSA.......................QDAEGHA.SPPAV
[Trichoderma_lentiforme]_KAF3077685.1_ RAAKLAADILAQTTA...ASLPSA.......................QDADDHATTPPAV
[Trichoderma_harzianum]_PNP59423.1 RAAKLAADILAQTAA...ASLPS........................ADGHA....TPAV
[Trichoderma_virens_FT-333]_UKZ73321.1 RAAKLAADILAQTTA...SSLSA........................HDGK.....PPAV
[Trichoderma_virens_Gv29-8]_XP_013960763.1 RAAKLAADILAQTTA...SLSSA........................HDGQ.....PPAV
[Trichoderma_asperellum_CBS_433.97]_XP_024756506. RAAKLAADILAQTST...H.............................KEHA....APTV
[Trichoderma_atroviride_IMI_206040]_XP_013943551. RAAKLAADILAQTSR...D.............................ERDA....PPTV
[Trichoderma_gamsii]_PNP38305.1 RAAKLAADIVAQTSR...D.............................KHE.....TPTV
[Trichoderma_gamsii]_XP_018663207.1 RAAKLAADIVAQTSR...D.............................KHE.....TPTV
[Trichoderma_arundinaceum]_RFU75476.1 RAAKLAADILAQTAS...SLDTP........................RNGQ.....APTV
[Trichoderma_gracile]_KAH0498535.1 RAEKLAADILAQTSA...AAAPSLIDS......DHNNNNRALRHNGGQNGQE....TPVV
[Trichoderma_reesei_QM6a]_XP_006968245.1 RAEKLAADILAQTSA...A...SL[illegible]HS......NNNNNNNNNKNTNDRDSRL....TPVV
[Trichoderma_citrinoviride]_XP_024753310.1 RAEKLAADILAQNQ..TPMV
[Trichoderma_longibrachiatum_ATCC_18648]_PTB78011 RAEKLAADILAQTSL...S..............NRDDKKLVRDGGRQGEANS....SPVV
[Hirsutella_minnesotensis_3608]_KJZ78693.1 RAAKLAQDIAAACPPCQASSSGP........................GAGNG....PVPV
[Hirsutella_rhossiliensis]_XP_044725020.1 RAAKLAADIAAAFPP...SSPAP........................RTGEG....TVPV
[Ophiocordyceps_sinensis_CO18]_EQK99704.1 RAAKLAADMAAALSS...S...P........................RAAQG....PISV
[Tolypocladium_capitatum]_PNY29418.1 RAAKLAADIAADTSA...SDPSP........................SPGRP....PTPV
[Tolypocladium_ophioglossoides_CBS_100239]_KND888 RAAKLAADIAAASAF....DPSP........................SPSR.....PTPV
[Tolypocladium_paradoxum]_POR38551.1 RAAKLAADIAAASSS...GT....................................PTPV
[Drechmeria_coniospora]_ODA83100.1 RAAKLAADISASIAS...SKFSG................................DPVPV
[Drechmeria_coniospora]_XP_040657415.1 RAAKLAADISASIAS...SKFSG................................DPVPV
[Ophiocordyceps_unilateralis]_PFH57814.1 RAAKLAADIATARS..PV
[Fusarium_sp._AF-6]_RSL54391.1 RAAALRSALEAHAPA...PAAV
[Fusarium_kuroshium]_RMJ16314.1 RAAALRSALEAHAPA...PEAV
[Fusarium_sp._AF-8]_RSL70798.1 RAAALRSALEAHAPA...PEAV
[Fusarium_ambrosium]_RSM11997.1 RAAALRSALEAHAPA...PEAV
[Fusarium_floridanum]_RSL80024.1 RAAALRSALEAHAPA...PEAV
[Fusarium_vanettenii_77-13-4]_XP_003050716.1 RAAALRSALEAHAPA...PEAV
[Fusarium_solani]_XP_046134414.1 RAAALRSALEAHAPA...PEAV
[Fusarium_albosuccineum]_KAF4468415.1 RAAALRTALESHAPA...PEAV
[Fusarium_decemcellulare]_KAF5011412.1 RAAALRTALESHAPA...PEAV
[Fusarium_sarcochroum]_KAF4964785.1 RAAALRSALEAHAPA...PEAF
[Fusarium_avenaceum]_KAH6968652.1 RAAALRSALEAQSPA...PEAF
[Fusarium_avenaceum]_KIL96274.1 RAAALRSALEAQSPA...PEAF
[Fusarium_avenaceum]_KAG5656265.1 RAAALRSALEAQSPA...PEAF
[Fusarium_tricinctum]_KAH7262197.1 RAAALRSALEAQSPA...PEAF
[Fusarium_heterosporum]_KAF5663380.1 RAAALRSALEAHTPA...LEAV
[Fusarium_sp._MPI-SDFR-AT-0072]_KAH7164412.1 RAAALRSALEAHAPM...HEAV
[Fusarium_oxysporum_f._sp._rapae]_KAG7414252.1 RAAALRSALEAHAPM...HEAV
[Fusarium_mangiferae]_XP_041681833.1 RATALRSALEAHAPM...PEAV
[Fusarium_proliferatum]_CVL11341.1 RATALRSALEAHAPM...PEAV
[Fusarium_proliferatum_ET1]_XP_031083673.1 RATALRSALEAHAPM...PEAV
[Fusarium_fujikuroi]_SCN78580.1 RATALRSALEAHAPM...PEAV
[Fusarium_fujikuroi_IMI_58289]_XP_023430709.1 RATALRSALEAHAPM...PEAV
[Fusarium_sp._NRRL_25303]_KAF5626228.1 RATALRSALEAHAPM...PEAV
[Fusarium_xylarioides]_KAG5746670.1 RATALRSALEAHAPM...PEAV
[Fusarium_xylarioides]_KAG5788219.1 RATALRSALEAHAPM...PEAV
[Fusarium_pseudocircinatum]_KAF5575212.1 RATALRSALEAHAPM...PEAV
[Fusarium_pseudoanthophilum]_KAF5575021.1 RATALRSALEAHAPM...PEAV
[Fusarium_verticillioides]_RBQ71412.1 RATALRSALEAHAPM...PEAV
[Fusarium_verticillioides_7600]_XP_018746479.1 RATALRSALEAHAPM...PEAV
[Fusarium_oxysporum_NRRL_32931]_XP_031039788.1 RATALRSALEALAPM...PQAV
[Fusarium_oxysporum_f._sp._lycopersici_4287]_XP_0 RATALRSALEALAPM...PQAV
[Fusarium_oxysporum_f._sp._cepae]_RKK20771.1 RATALRSALEALAPM...PQAV
[Fusarium_redolens]_XP_046051341.1 RAAALRSALEAHAPM...PEAV
[Fusarium_austroafricanum]_KAF4458007.1 RAAALRSALEAYAPM...PEAV
[Fusarium_flagelliforme]_XP_045986929.1 RAAALRSALEAHAPA...PEAF
[Fusarium_sp._FIESC_RH6]_KAI1056250.1 RAAALRSALEAHAPA...PEAF
[Fusarium_sporotrichioides]_RGP72309.1 RAAALRSALEAHAPA...PEAV
[Fusarium_poae]_XP_044713592.1 RAAALRSALEAHAPV...PEAV
[Fusarium_venenatum]_XP_025590637.1 RAAALRSALEAHAPV...PEAV
[Fusarium_pseudograminearum]_KAF0641638.1 RAAALRSALEAHAPA...PEAV
[Fusarium_graminearum]_CAG1993854.1 RAAALRSALEAHAPA...PEAV
[Fusarium_graminearum]_PCD31638.1 RAAALRSALEAHAPA...PEAV
[Fusarium_graminearum]_CZS78629.1 RAAALRSALEAHAPA...PEAV
[Fusarium_graminearum]_CAF3606094.1 RAAALRSALEAHAPA...PEAV
[Fusarium_graminearum_PH-1]_XP_011318944.1 RAAALRSALEAHAPA...PEAV
[Fusarium_culmorum]_QPC68571.1 RAAALRSALEAHAPA...PEAV
[Fusarium_pseudograminearum]_QPC77441.1 RAAALRSALEAHAPA...PEAV
[Fusarium_longipes]_RGP79158.1 RAAALRSALEAHAPV...PEAV
[Ilyonectria_destructans]_KAH7011941.1 RAAALHAALAECAPA...PDAV
[Ilyonectria_sp._MPI-CAGE-AT-0026]_KAH6985660.1 RAAALHAALAECAPA...PDAV
[Ilyonectria_robusta]_XP_046104990.1 RAAALHAALAECAPA...PDAV
[Dactylonectria_estremocensis]_KAH7149692.1 RAAALHAALAACDPK...PEAV
[Dactylonectria_macrodidyma]_KAH7148923.1 RAAALHEALAACEPK...PEAV
[Thelonectria_olida]_KAH6899778.1 RAAALHDALAACSPM...PEAV
[Stachybotrys_chartarum_IBT_7711]_KEY69997.1 RAAKLGADIAAQAPP...NVPV
[Stachybotrys_chartarum_IBT_40293]_KFA50745.1 RAAKLGADIAAQAPP...NVPV
[Stachybotrys_chlorohalonata_IBT_40285]_KFA62815. RAAKLGADIASQAPP...NVPV
[Purpureocillium_lilacinum]_XP_018174914.1 RAAKLAVEISSALSS...SSSSSSVASSSPFPASRGSSG.AADVGARGDDDT....RTPV
[Clonostachys_solani]_CAH0059244.1 RAASLRDQIAAAR...PSAV
[Clonostachys_rosea]_VUC36940.1 RAASLRDHITAAR...PSAV
[Clonostachys_rhizophaga]_CAH0026503.1 RAASLRDQIAAAR...PSAV
[Emericellopsis_atlantica]_XP_046114137.1 RAKKLREDIDAAAST...TR....................................DGRV
[Acremonium_chrysogenum_ATCC_11550]_KFH46845.1 RAAKLRDDIDASTRT...TDGSR.................................AEKV
[Pochonia_chlamydosporia_170]_XP_018148143.1 RAAKLADDITSAGVD...V.....................................SAGV
[Epichloe_festucae_Fl1]_QPG98063.1 RAAKLADEIAARHSG...PV
[Colletotrichum_incanum]_KZL82863.1 RAAKLGADIAAKAG..GRAV
[Colletotrichum_simmondsii]_KXH39849.1 RAAKLGADIAAKAG..GRAV
[Colletotrichum_sojae]_KAF6819546.1 RAAKLGADIAAKAR..GRSV

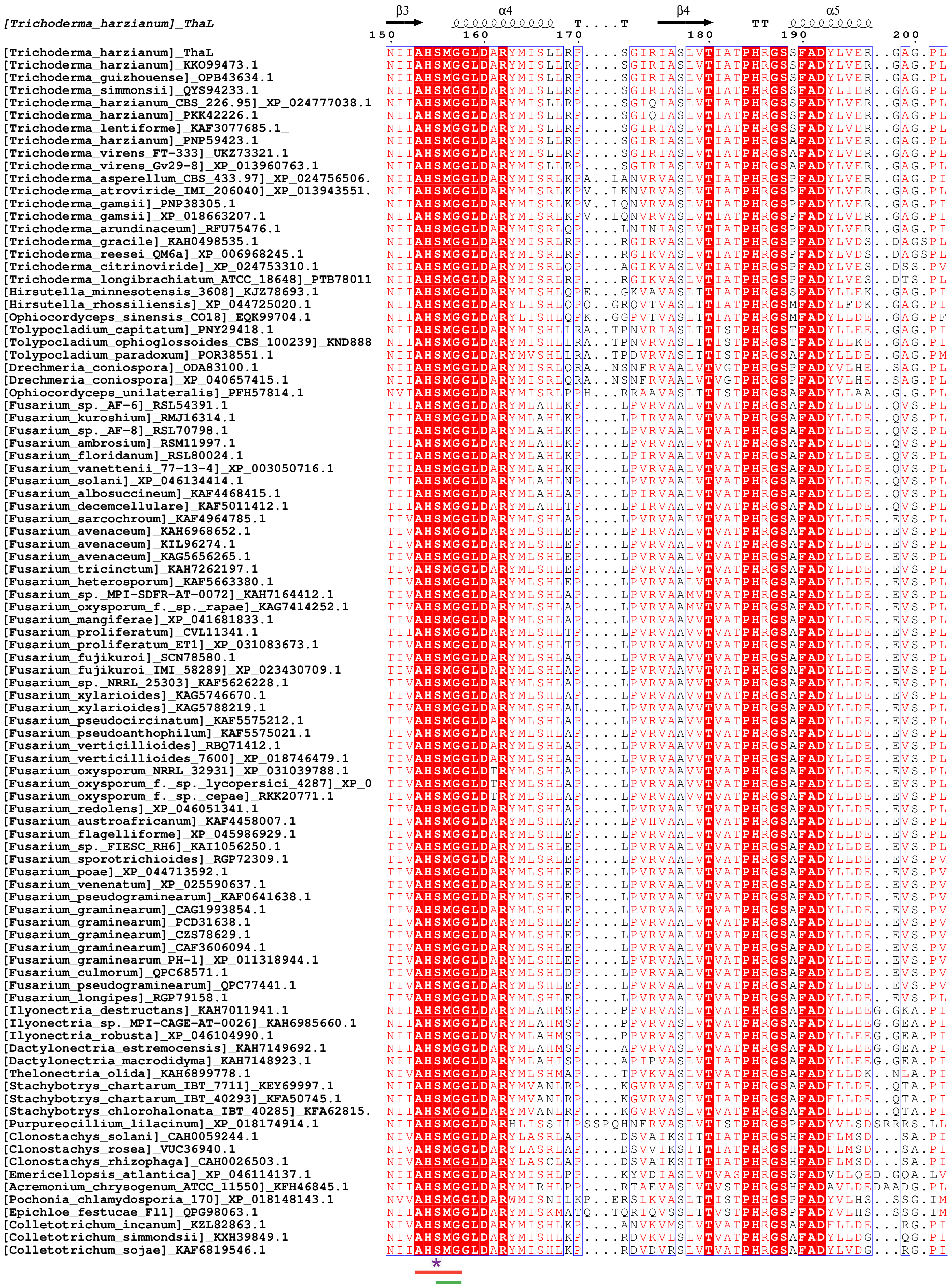

[Trichoderma_harzianum]_ThaL
β3
α4
β4
α5
TT
150
160
170
180
190
200

```
                                                       α6                 α7            α8                     β5
[Trichoderma_harzianum]_ThaL                           ..                                                 TT  ------->
                                                             210       220       230       240       250
[Trichoderma_harzianum]_ThaL                           H..LPRLYGVIRRAGLGTSAFGQLTTRYMREEFNPRVRDDEAVRYFSYGAA.IDEP..SL
[Trichoderma_harzianum]_KKO99473.1                     H..LPRLYGVIRRAGLGTSAFGQLTTRYMREEFNPRVRDDEAVRYFSYGAA.IDEP..SL
[Trichoderma_guizhouense]_OPB43634.1                   H..LPRLYGVIRRAGLGTSAFGQLTTRYMREEFNPRVRDDEGVRYFSYGAA.IDEP..SL
[Trichoderma_simmonsii]_QYS94233.1                     H..LPRLYGVIRRAGLGTSAFGQLTTRYMREEFNPRVRDDEGVRYFSYGAA.IDEP..SL
[Trichoderma_harzianum_CBS_226.95]_XP_024777038.1      H..LPRLYGVIRRAGLGTSAFGQLTTRYMREEFNPRVRDDEGVRYFSYGAA.IDEP..SL
[Trichoderma_harzianum]_PKK42226.1                     H..LPRLYGVIRRAGLGTSAFGQLTTRYMREEFNPRVRDDEGVRYFSYGAA.IDEP..SL
[Trichoderma_lentiforme]_KAF3077685.1_                 H..LPRLYGVIRRAGLGTSAFGQLTTRYMREEFNPRVRDEEGVRYFSYGAA.IDEP..SL
[Trichoderma_harzianum]_PNP59423.1                     H..LPRLYGVIRRAGLGTSAFGQLTTRYMRDEFNPRVRDDEAVRYFSYGAA.IDEP..SL
[Trichoderma_virens_FT-333]_UKZ73321.1                 H..LPRLYGVIRRAGLGTSAFGQLTTRYMRDEFNPRVRDDEAVRYFSYGAA.IDEP..SL
[Trichoderma_virens_Gv29-8]_XP_013960763.1             H..LPRLYGVIRRAGLGTSAFGQLTTRYMRDEFNPRVRDDEAVRYFSYGAA.IDEP..SL
[Trichoderma_asperellum_CBS_433.97]_XP_024756506.      H..LPRLYGIIRRAGLGTSAFGQLTTRYMREEFNPRVRDDESVRYFSYGAA.IDEP..SL
[Trichoderma_atroviride_IMI_206040]_XP_013943551.      H..LPRLYSIIRRAGLGTSAFGQLTTRYMRDEFNPRVRDDEAVRYFSYGAA.IDEP..SL
[Trichoderma_gamsii]_PNP38305.1                        H..LPRLYSIIRRAGLGTSAFGQLTTRYMREEFNTKVLDDEAVRYFSYGAA.IDEP..SL
[Trichoderma_gamsii]_XP_018663207.1                    H..LPRLYSIIRRAGLGTSAFGQLTTRYMREEFNTKVLDDEAVRYFSYGAA.IDEP..SL
[Trichoderma_arundinaceum]_RFU75476.1                  H..LPRLYGIIRRAGLGTSAFGQLTTRYMREEFNPRVQDDESVRYFSYGAA.IDEP..SL
[Trichoderma_gracile]_KAH0498535.1                     H..LPRLYSVIRRAGLGTSAFGQLTTRYMRDEFNPRVPDDERVRYFSYGAM.IDEP..SL
[Trichoderma_reesei_QM6a]_XP_006968245.1               H..LPRLYSVIRRAGLGTSAFGQLTTRYMRHEFNPRVPDDQAVRYFSYGAM.IDEP..SL
[Trichoderma_citrinoviride]_XP_024753310.1             H..LPRLYSVIRRAGLGTSAFGQLTTRYMRDEFNPRVPDDPAVRYFSYGAM.IDEP..SL
[Trichoderma_longibrachiatum_ATCC_18648]_PTB78011      H..LPRLYSVIRRAGLGTSAFGQLTTRYMRDEFNPRVPDVEGVRYFSYGAM.IDEP..SL
[Hirsutella_minnesotensis_3608]_KJZ78693.1             Y..LPKLYGVLKKAGLGTQAFAQLTTHYMVNEFNPQNPDNPSVRYFSYGSV.TDTP..PV
[Hirsutella_rhossiliensis]_XP_044725020.1              Y..LPKLYRLLEKAGLGTKAFAQLTTRYMAQEFNPQTPDDPLVRYYSYGAV.IDKP..PL
[Ophiocordyceps_sinensis_CO18]_EQK99704.1              H..LPRLYRVLEKAGLGTKAFAQLTTRYMAQEFNPQTPDDPSVRYFSYGAV.SDAP..SL
[Tolypocladium_capitatum]_PNY29418.1                   Y..LPRLYGVLQRAGLGTKAFAQLTTRYLADEFNPRTEDDPSVRYFSYGAA.MNEP..PL
[Tolypocladium_ophioglossoides_CBS_100239]_KND888      Y..LPKLYGVLQRAGLGTKAFAQLTTRYMADEFNPRTVDDPSVRYFSYGAV.MNEP..PL
[Tolypocladium_paradoxum]_POR38551.1                   H..LPRLYGVLQRAGLGTRAFAQLTTRYMADEFNPRTEDDPEVRYFSYGAV.MREP..PL
[Drechmeria_coniospora]_ODA83100.1                     Y..LPRLYSLLERAGLGTRAFAQLTTRYLATEFNPRTRDDPAVRYFSYGAV.VEDL..PL
[Drechmeria_coniospora]_XP_040657415.1                 Y..LPRLYSLLERAGLGTRAFAQLTTRYLATEFNPRTRDDPAVRYFSYGAV.VEDL..PL
[Ophiocordyceps_unilateralis]_PFH57814.1               H..LPRLYGLLRRVGLGTQAFEQLTTLYMTDDFNPRTEDDPSVRYFSYGAV.APSP..SL
[Fusarium_sp._AF-6]_RSL54391.1                         H..LPRLYWMLERAGLGTAAFAQLTRRYMADEFNPSTPDCSDVHYFSYGAA.VERP..AL
[Fusarium_kuroshium]_RMJ16314.1                        H..LPRLYWMLERAGLGTAAFAQLTRRYMADEFNPSTPDCPDVHYFSYGAA.VERP..AL
[Fusarium_sp._AF-8]_RSL70798.1                         H..LPRLYWMLERAGLGTAAFAQLTRRYMADEFNPSTPDCPDVHYFSYGAA.VERP..AL
[Fusarium_ambrosium]_RSM11997.1                        H..LPRLYWMLERAGLGTAAFAQLTRRYMADEFNPSTPDCPDVHYFSYGAA.VERP..AL
[Fusarium_floridanum]_RSL80024.1                       H..LPRLYWMLERAGLGTAAFAQLTRRYMADEFNPSTPDCPDVHYFSYGAA.VERP..AL
[Fusarium_vanettenii_77-13-4]_XP_003050716.1           H..LTRLYWMLERAGLGTAAFAQLTRRYMADEFNPSTPDNPDVHYFSYGAA.VERP..AL
[Fusarium_solani]_XP_046134414.1                       H..LPRLYWMLERAGLGTAAFAQLTRRYMTDEFNPSTPDCPDVHYFSYGAA.VERP..AL
[Fusarium_albosuccineum]_KAF4468415.1                  H..LPRLYKTLERAGLGTAAFAQLTRRYMADEFNPSTPDCADVRYFSYGAA.VEQP..AM
[Fusarium_decemcellulare]_KAF5011412.1                 H..LPRLYKTLERAGLGTAAFAQLTRRYMADEFNPSTPDCADVRYFSYGAA.VERP..AL
[Fusarium_sarcochroum]_KAF4964785.1                    HRYLPRLYHTLEKAGLGTAAFSQLTRRYMAEEFNPSTPDKPDVRYFSYGAA.VEQP..PL
[Fusarium_avenaceum]_KAH6968652.1                      HQYLPRLYQTFEKAGLGTAAFSQLTRRYMAEEFNPSTPDRPDVRYFSYGAA.VEQP..AL
[Fusarium_avenaceum]_KIL96274.1                        HQYLPRLYQTFEKAGLGTAAFSQLTRRYMAEEFNPSTPDRPDVRYFSYGAA.VEQP..AL
[Fusarium_avenaceum]_KAG5656265.1                      HQYLPRLYQTFEKAGLGTAAFSQLTRRYMAEEFNPSTPDRPDVRYFSYGAA.VEQP..AL
[Fusarium_tricinctum]_KAH7262197.1                     HHYLPRLYQTFEKAGLGTAAFSQLTRRYMAEEFNPSTPDRPDVRYFSYGAA.VEHP..AL
[Fusarium_heterosporum]_KAF5663380.1                   HHYLPRLYHTLEKAGLGTAAFSQLTRRYMAEEFNPSTPDQPDVRYFSYGAA.VEQP..AL
[Fusarium_sp._MPI-SDFR-AT-0072]_KAH7164412.1           HHYLPQLYQTFEKAGLGTAAFSQLTRRYMAKEFNPSTPDQPGVRYFSYGAA.VERP..AL
[Fusarium_oxysporum_f._sp._rapae]_KAG7414252.1         HHYLPQLYQTFEKAGLGTAAFSQLTRRYMAKEFNPSTPDQPDVRYFSYGAA.VERP..AL
[Fusarium_mangiferae]_XP_041681833.1                   HHYLPQLYQTFEKAGLGTAAFSQLTRRYMAEEFNPSTPDQPDVRYFSYGAA.VERP..AL
[Fusarium_proliferatum]_CVL11341.1                     HHYLPQLYQTFEKAGLGTAAFSQLTRRYMAEEFNPSTPDQPDVRYFSYGAA.VERP..AL
[Fusarium_proliferatum_ET1]_XP_031083673.1             HHYLPQLYQTFEKAGLGTAAFSQLTRRYMAEEFNPSTPDQPDVRYFSYGAA.VERP..AL
[Fusarium_fujikuroi]_SCN78580.1                        HHYLPQLYQTFEKAGLGTAAFSQLTRRYMAEEFNPSTPDQPDVRYFSYGAA.VERP..AL
[Fusarium_fujikuroi_IMI_58289]_XP_023430709.1          HHYLPQLYQTFEKAGLGTAAFSQLTRRYMAEEFNPSTPDQPDVRYFSYGAA.VERP..AL
[Fusarium_sp._NRRL_25303]_KAF5626228.1                 HHYLPQLYQTLEKAGLGTAAFSQLTRRYMAEEFNPSTPDQPDVRYFSYGAA.VERP..AL
[Fusarium_xylarioides]_KAG5746670.1                    HHYLPQLYQTFEKAGLGTAAFSQLTRRYMAEEFNPSTPDQPDVRYFSYGAA.VERP..AL
[Fusarium_xylarioides]_KAG5788219.1                    HHYLPQLYQTFEKAGLGTAAFSQLTRRYMAEEFNPSTPDQPDVRYFSYGAA.VERP..AL
[Fusarium_pseudocircinatum]_KAF5575212.1               HHYLPQLYQTFEKAGLGTAAFSQLTRRYMAEEFNPSTPDQPDVRYFSYGAA.VERP..AL
[Fusarium_pseudoanthophilum]_KAF5575021.1              HHYLPQLYQTFEKAGLGTAAFSQLTRRYMAEEFNPSTPDQPDVRYFSYGAA.VERP..AL
[Fusarium_verticillioides]_RBQ71412.1                  HHYLPQLYQTFEKAGLGTAAFSQLTRRYMAEEFNPSTPDQPDVRYFSYGAA.VERP..AL
[Fusarium_verticillioides_7600]_XP_018746479.1         HHYLPQLYQTFEKAGLGTAAFSQLTRRYMAEEFNPSTPDQPDVRYFSYGAA.VERP..AL
[Fusarium_oxysporum_NRRL_32931]_XP_031039788.1         HHYLPQLYQTFEKAGLGTAAFSQLTRRYMAEEFNPSTPDQPDVRYFSYGAA.VERP..AL
[Fusarium_oxysporum_f._sp._lycopersici_4287]_XP_0      HHYLPQLYQTFEKAGLGTAAFSQLTRRYMAEEFNPSTPDQPDVRYFSYGAA.VERP..AL
[Fusarium_oxysporum_f._sp._cepae]_RKK20771.1           HHYLPQLYQTFEKAGLGTAAFSQLTRRYMAEEFNPSTPDQPDVRYFSYGAA.VERP..AL
[Fusarium_redolens]_XP_046051341.1                     HHYLPRLYQTFEKAGLGTAAFSQLTRRYMAEEFNPSTPDQPDVRYFSYGAA.VERP..AL
[Fusarium_austroafricanum]_KAF4458007.1                HQYLPQLYQTLEKAGLGTAAFSQLTRRYMAEEFNPSTPDQPDVRYFSYGAA.VERP..AL
[Fusarium_flagelliforme]_XP_045986929.1                HRYLPRLYQTFERAGLGTAAFSQLTRRYMAEEFNPSTPDRADVRYFSYGAA.VERP..AL
[Fusarium_sp._FIESC_RH6]_KAI1056250.1                  HQYLPRLYQTFERAGLGTAAFSQLTRRYMAEEFNPSTPDRADVRYFSYGAA.VERP..AL
[Fusarium_sporotrichioides]_RGP72309.1                 HHYLPQLYKTLERAGLGTAAFSQLTRRYMAEEFNPSTPDQPDVRYFSYGAA.VERP..AL
[Fusarium_poae]_XP_044713592.1                         HHYLPQLYKTLERAGLGTAAFSQLTRRYMAEEFNPSTPDQPDVRYFSYGAA.VERP..AL
[Fusarium_venenatum]_XP_025590637.1                    HHYLPQLYKTLERAGLGTAAFSQLTRRYMAEEFNPSTPDQPDVRYFSYGAA.VERP..AL
[Fusarium_pseudograminearum]_KAF0641638.1              HHYLPQLYKTLERAGLGTAAFSQLTRRYMAEEFNPSTPDQPDVRYFSYGAA.VERP..AL
[Fusarium_graminearum]_CAG1993854.1                    HHYLPQLYKTLERAGLGTAAFSQLTRRYMAEEFNPSTPDQPDVRYFSYGAA.VERP..AL
[Fusarium_graminearum]_PCD31638.1                      HHYLPQLYKTLERAGLGTAAFSQLTRRYMAEEFNPSTPDQPDVRYFSYGAA.VERP..AL
[Fusarium_graminearum]_CZS78629.1                      HHYLPQLYKTLERAGLGTAAFSQLTRRYMAEEFNPSTPDQPDVRYFSYGAA.VERP..AL
[Fusarium_graminearum]_CAF3606094.1                    HHYLPQLYKTLERAGLGTAAFSQLTRRYMAEEFNPSTPDQPDVRYFSYGAA.VERP..AL
[Fusarium_graminearum_PH-1]_XP_011318944.1             HHYLPQLYKTLERAGLGTAAFSQLTRRYMAEEFNPSTPDQPDVRYFSYGAA.VERP..AL
[Fusarium_culmorum]_QPC68571.1                         HHYLPQLYKTLERAGLGTAAFSQLTRRYMAEEFNPSTPDQPDVRYFSYGAA.VERP..AL
[Fusarium_pseudograminearum]_QPC77441.1                HHYLPQLYKTLERAGLGTAAFSQLTRRYMAEEFNPSTPDQPDVRYFSYGAA.VERP..AL
[Fusarium_longipes]_RGP79158.1                         HHYLPQLYKTLERAGLGTAAFSQLTRRYMAEEFNPNTPDQPDVRYFSYGAA.VERP..AL
[Ilyonectria_destructans]_KAH7011941.1                 H..LPRLYGAIERAGFGTAAFAQLTRRYMQSEFNPATPDSPDVRYFSYGAAFVKTP..PL
[Ilyonectria_sp._MPI-CAGE-AT-0026]_KAH6985660.1        H..LPRLYGAIERAGFGTAAFAQLTRRYMQSEFNPATPDSPDVRYFSYGAAFVKTP..PL
[Ilyonectria_robusta]_XP_046104990.1                   H..LPRLYGAIERAGFGTAAFAQLTRRYMQSEFNPATPDSPDVRYFSYGAAFVKTP..PL
[Dactylonectria_estremocensis]_KAH7149692.1            H..LPRLYGAIERAGFGTAAFAQLTRRYMQDEFNPATRDSPDVRYFSYGAAFVETP..PL
[Dactylonectria_macrodidyma]_KAH7148923.1              H..LPRLYGAIERAGFGTAAFAQLTQRYMQDEFNPATQDSPDVRYFSYGAAFVGTP..PL
[Thelonectria_olida]_KAH6899778.1                      H..LPRLYGAIERAGFGTAAFAQLTRRYMTDEFNAATPDDPDVQYFSYGAALEDVP..PL
[Stachybotrys_chartarum_IBT_7711]_KEY69997.1           H..LPKLYWALERTGLGTRAFAQLTRRYLTEEFNSATKDIPGVQYYSYGASLTQPP..PL
[Stachybotrys_chartarum_IBT_40293]_KFA50745.1          H..LPKLYWALERTGLGTRAFAQLTRRYLTEEFNSATKDIPGVQYYSYGASLTQPP..PL
[Stachybotrys_chlorohalonata_IBT_40285]_KFA62815.      Y..LPKLYWALERTGLGTQAFAQLTKRYLTEEFNSATKDVPGVQYYSYGASLTQPP..PL
[Purpureocillium_lilacinum]_XP_018174914.1             H..LPRFYSLFRAAGLSTRAFAQLGTRYLADDFNPANPDDPAVRYFSYGAA.LHTAQLPM
[Clonostachys_solani]_CAH0059244.1                     H..LPRLYGLIERAGLGTQAFSQLTREYMTGTFNATVRDRDDVRYFSYGAM.MDEP..AL
[Clonostachys_rosea]_VUC36940.1                        H..LPRLYGLIERAGFGTQAFSQLTREYMTQTFNATVQDRDDVRYFSYGAM.MDEP..AL
[Clonostachys_rhizophaga]_CAH0026503.1                 H..LPRLYGLIERAGFGTQAFSQLTREYMTQTFNATVRDRDDVRYFSYGAM.MDEP..AL
[Emericellopsis_atlantica]_XP_046114137.1              H..LPKLYGLIERFGLGTAAFAQLTTSHMTKVFNPATPDSPSTRYFSYGAS.TTEP..PL
[Acremonium_chrysogenum_ATCC_11550]_KFH46845.1         Y..LPRLYGLLERAGLDTAAFAQLTRAYMTGTFNPATPDRDSVQYFSYGAR.CDEP..PL
[Pochonia_chlamydosporia_170]_XP_018148143.1           Y..LPRLYRLLERAGLSTSAFSQLTTSYMRDEFNPRTRDDEAVRYFSYGAM.MAPP..PL
[Epichloe_festucae_Fl1]_QPG98063.1                     Y..LPRLYGVLERAGLGTKAFAQLTTGYMTGTFNRDVVDDDKVRYFSYGAV.MDPP..PL
[Colletotrichum_incanum]_KZL82863.1                    K..LANLYGLIERAGLGTQAFGQLTQKYMEEEFNPRTPDNDEVRYFSYGAC.TDRP..PL
[Colletotrichum_simmondsii]_KXH39849.1                 K..LANLYGLIERAGLGTQAFEQLTQRYMEEKFNPATPDSDEVRYFSYGAC.TDRP..PL
[Colletotrichum_sojae]_KAF6819546.1                    K..LANLYGLIERAGLGTRAFGQLTRDYMEREFNPATPDSERVRYFSYGAC.TGTP..PL
```

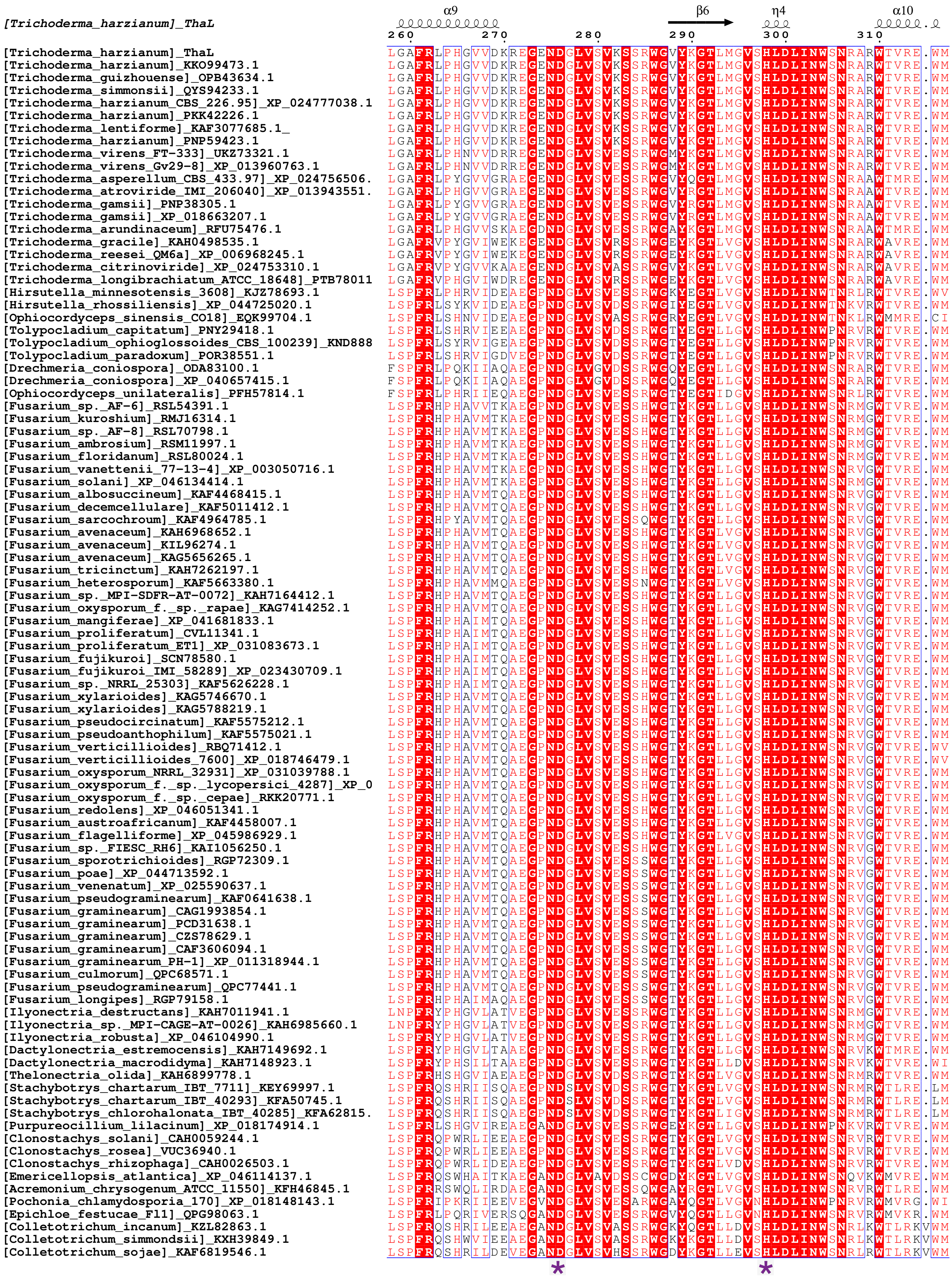
[Trichoderma_harzianum]_ThaL
α9
β6
η4
α10
260
270
280
290
300
310
[Trichoderma_harzianum]_ThaL
[Trichoderma_harzianum]_KKO99473.1
[Trichoderma_guizhouense]_OPB43634.1
[Trichoderma_simmonsii]_QYS94233.1
[Trichoderma_harzianum_CBS_226.95]_XP_024777038.1
[Trichoderma_harzianum]_PKK42226.1
[Trichoderma_lentiforme]_KAF3077685.1_
[Trichoderma_harzianum]_PNP59423.1
[Trichoderma_virens_FT-333]_UKZ73321.1
[Trichoderma_virens_Gv29-8]_XP_013960763.1
[Trichoderma_asperellum_CBS_433.97]_XP_024756506.
[Trichoderma_atroviride_IMI_206040]_XP_013943551.
[Trichoderma_gamsii]_PNP38305.1
[Trichoderma_gamsii]_XP_018663207.1
[Trichoderma_arundinaceum]_RFU75476.1
[Trichoderma_gracile]_KAH0498535.1
[Trichoderma_reesei_QM6a]_XP_006968245.1
[Trichoderma_citrinoviride]_XP_024753310.1
[Trichoderma_longibrachiatum_ATCC_18648]_PTB78011
[Hirsutella_minnesotensis_3608]_KJZ78693.1
[Hirsutella_rhossiliensis]_XP_044725020.1
[Ophiocordyceps_sinensis_CO18]_EQK99704.1
[Tolypocladium_capitatum]_PNY29418.1
[Tolypocladium_ophioglossoides_CBS_100239]_KND888
[Tolypocladium_paradoxum]_POR38551.1
[Drechmeria_coniospora]_ODA83100.1
[Drechmeria_coniospora]_XP_040657415.1
[Ophiocordyceps_unilateralis]_PFH57814.1
[Fusarium_sp._AF-6]_RSL54391.1
[Fusarium_kuroshium]_RMJ16314.1
[Fusarium_sp._AF-8]_RSL70798.1
[Fusarium_ambrosium]_RSM11997.1
[Fusarium_floridanum]_RSL80024.1
[Fusarium_vanettenii_77-13-4]_XP_003050716.1
[Fusarium_solani]_XP_046134414.1
[Fusarium_albosuccineum]_KAF4468415.1
[Fusarium_decemcellulare]_KAF5011412.1
[Fusarium_sarcochroum]_KAF4964785.1
[Fusarium_avenaceum]_KAH6968652.1
[Fusarium_avenaceum]_KIL96274.1
[Fusarium_avenaceum]_KAG5656265.1
[Fusarium_tricinctum]_KAH7262197.1
[Fusarium_heterosporum]_KAF5663380.1
[Fusarium_sp._MPI-SDFR-AT-0072]_KAH7164412.1
[Fusarium_oxysporum_f._sp._rapae]_KAG7414252.1
[Fusarium_mangiferae]_XP_041681833.1
[Fusarium_proliferatum]_CVL11341.1
[Fusarium_proliferatum_ET1]_XP_031083673.1
[Fusarium_fujikuroi]_SCN78580.1
[Fusarium_fujikuroi_IMI_58289]_XP_023430709.1
[Fusarium_sp._NRRL_25303]_KAF5626228.1
[Fusarium_xylarioides]_KAG5746670.1
[Fusarium_xylarioides]_KAG5788219.1
[Fusarium_pseudocircinatum]_KAF5575212.1
[Fusarium_pseudoanthophilum]_KAF5575021.1
[Fusarium_verticillioides]_RBQ71412.1
[Fusarium_verticillioides_7600]_XP_018746479.1
[Fusarium_oxysporum_NRRL_32931]_XP_031039788.1
[Fusarium_oxysporum_f._sp._lycopersici_4287]_XP_0
[Fusarium_oxysporum_f._sp._cepae]_RKK20771.1
[Fusarium_redolens]_XP_046051341.1
[Fusarium_austroafricanum]_KAF4458007.1
[Fusarium_flagelliforme]_XP_045986929.1
[Fusarium_sp._FIESC_RH6]_KAI1056250.1
[Fusarium_sporotrichioides]_RGP72309.1
[Fusarium_poae]_XP_044713592.1
[Fusarium_venenatum]_XP_025590637.1
[Fusarium_pseudograminearum]_KAF0641638.1
[Fusarium_graminearum]_CAG1993854.1
[Fusarium_graminearum]_PCD31638.1
[Fusarium_graminearum]_CZS78629.1
[Fusarium_graminearum]_CAF3606094.1
[Fusarium_graminearum_PH-1]_XP_011318944.1
[Fusarium_culmorum]_QPC68571.1
[Fusarium_pseudograminearum]_QPC77441.1
[Fusarium_longipes]_RGP79158.1
[Ilyonectria_destructans]_KAH7011941.1
[Ilyonectria_sp._MPI-CAGE-AT-0026]_KAH6985660.1
[Ilyonectria_robusta]_XP_046104990.1
[Dactylonectria_estremocensis]_KAH7149692.1
[Dactylonectria_macrodidyma]_KAH7148923.1
[Thelonectria_olida]_KAH6899778.1
[Stachybotrys_chartarum_IBT_7711]_KEY69997.1
[Stachybotrys_chartarum_IBT_40293]_KFA50745.1
[Stachybotrys_chlorohalonata_IBT_40285]_KFA62815.
[Purpureocillium_lilacinum]_XP_018174914.1
[Clonostachys_solani]_CAH0059244.1
[Clonostachys_rosea]_VUC36940.1
[Clonostachys_rhizophaga]_CAH0026503.1
[Emericellopsis_atlantica]_XP_046114137.1
[Acremonium_chrysogenum_ATCC_11550]_KFH46845.1
[Pochonia_chlamydosporia_170]_XP_018148143.1
[Epichloe_festucae_Fl1]_QPG98063.1
[Colletotrichum_incanum]_KZL82863.1
[Colletotrichum_simmondsii]_KXH39849.1
[Colletotrichum_sojae]_KAF6819546.1

```
                                                              α11
[Trichoderma_harzianum]_ThaL                                  320       330       340
[Trichoderma_harzianum]_ThaL                             GMRRTFNAVAFYLDVADMLAKEGL.....................................
[Trichoderma_harzianum]_KKO99473.1                       GMRRTFNAVAFYLDVADMLAKEGL.....................................
[Trichoderma_guizhouense]_OPB43634.1                     GMRRTFNAVAFYLDVADMLAKEGL.....................................
[Trichoderma_simmonsii]_QYS94233.1                       GMRRTFNAVAFYLDVADMLAKEGL.....................................
[Trichoderma_harzianum_CBS_226.95]_XP_024777038.1        GMRRTFNAVAFYLDVADMLAKEGL.....................................
[Trichoderma_harzianum]_PKK42226.1                       GMRRTFNAVAFYLDVADMLAKEGL.....................................
[Trichoderma_lentiforme]_KAF3077685.1_                   GMRRTFNAVAFYLDVADMLAKEGL.....................................
[Trichoderma_harzianum]_PNP59423.1                       GMRRTFNAVAFYLDVADMLAKEGL.....................................
[Trichoderma_virens_FT-333]_UKZ73321.1                   GMRRTFNAVAFYLDVVDMLAKEGL.....................................
[Trichoderma_virens_Gv29-8]_XP_013960763.1               GMRRTFNAVAFYLDVVDMLAKEGL.....................................
[Trichoderma_asperellum_CBS_433.97]_XP_024756506.        GMKRTFNAVAFYLDVADMLAKEGL.....................................
[Trichoderma_atroviride_IMI_206040]_XP_013943551.        GMKRTFNAVAFYLDVADMLAKEGL.....................................
[Trichoderma_gamsii]_PNP38305.1                          GMKRTFNAVAFYLDVADMLAKEGL.....................................
[Trichoderma_gamsii]_XP_018663207.1                      GMKRTFNAVAFYLDVADMLAKEGL.....................................
[Trichoderma_arundinaceum]_RFU75476.1                    GMKRTF.......................................................
[Trichoderma_gracile]_KAH0498535.1                       GMKRTFNAVAFYLDVADMLAKEGL.....................................
[Trichoderma_reesei_QM6a]_XP_006968245.1                 GMKRTFNAVAFYLDVADMLAKEGL.....................................
[Trichoderma_citrinoviride]_XP_024753310.1               GMRRNFNAVAFYLDVADMLAKEGL.....................................
[Trichoderma_longibrachiatum_ATCC_18648]_PTB78011        GMRRTFNAVAFYLDVADMLAKEGL.....................................
[Hirsutella_minnesotensis_3608]_KJZ78693.1               GIKRTFNAIAFYLAITDMLAKQGL.....................................
[Hirsutella_rhossiliensis]_XP_044725020.1                GYKKTFNAIAFYLAITDMLAREGL.....................................
[Ophiocordyceps_sinensis_CO18]_EQK99704.1                GIKRTFNAIAFYLAITDMLAREGL.....................................
[Tolypocladium_capitatum]_PNY29418.1                     GMKRTFNAIAFYLGITDMLAKEGL.....................................
[Tolypocladium_ophioglossoides_CBS_100239]_KND888        GRKRTFNAIAFYLGITDMLAKEGL.....................................
[Tolypocladium_paradoxum]_POR38551.1                     GTKRAFNAIAFYLGITDMLAKAGL.....................................
[Drechmeria_coniospora]_ODA83100.1                       GVKRNFNAVAFYLGIADMLAREGL.....................................
[Drechmeria_coniospora]_XP_040657415.1                   GVKRNFNAVAFYLGIADMLAREGL.....................................
[Ophiocordyceps_unilateralis]_PFH57814.1                 GVRRNFNAIAFYLAITDRLAKEGF.....................................
[Fusarium_sp._AF-6]_RSL54391.1                           GIRRTFNAIAFYLDIADMLAKEGY.....................................
[Fusarium_kuroshium]_RMJ16314.1                          GIRRTFNAIAFYLDIADMLAKEGY.....................................
[Fusarium_sp._AF-8]_RSL70798.1                           GIRRTFNAIAFYLDIADMLAKEGY.....................................
[Fusarium_ambrosium]_RSM11997.1                          GIRRTFNAIAFYLDIADMLAKEGY.....................................
[Fusarium_floridanum]_RSL80024.1                         GIRRTFNAIAFYLDIADMLAKEGY.....................................
[Fusarium_vanettenii_77-13-4]_XP_003050716.1             GIRRTFNAIAFYLDIADMLAKEGY.....................................
[Fusarium_solani]_XP_046134414.1                         GIRRTFNAIAFYLDIADMLAKEGY.....................................
[Fusarium_albosuccineum]_KAF4468415.1                    GIKRTFNAIAFYLDIADMLAKEGF.....................................
[Fusarium_decemcellulare]_KAF5011412.1                   GIKRTFNAIAFYLDIADMLAKEGF.....................................
[Fusarium_sarcochroum]_KAF4964785.1                      GIKKNFNAIAYYLDIADMLAKEGY.....................................
[Fusarium_avenaceum]_KAH6968652.1                        GIKKNFNAIAFYLDIADMLAKEGY.....................................
[Fusarium_avenaceum]_KIL96274.1                          GIKKNFNAIAFYLDIADMLAKEGY.....................................
[Fusarium_avenaceum]_KAG5656265.1                        GIKKNFNAIAFYLDIADMLAKEGY.....................................
[Fusarium_tricinctum]_KAH7262197.1                       GIKKNFNAIAFYLDIADMLAKEGY.....................................
[Fusarium_heterosporum]_KAF5663380.1                     GIKRNFNAIAFYLDIADMLAKEGY.....................................
[Fusarium_sp._MPI-SDFR-AT-0072]_KAH7164412.1             GIKKNFNAIAFYLDIADMLAKEGY.....................................
[Fusarium_oxysporum_f._sp._rapae]_KAG7414252.1           GIKKNFNAIAFYLDIADMLAKEGY.....................................
[Fusarium_mangiferae]_XP_041681833.1                     GIKKNFNAIAFYLDIADMLAKEGY.....................................
[Fusarium_proliferatum]_CVL11341.1                       GIKKNFNAIAFYLDIADMLAKEGY.....................................
[Fusarium_proliferatum_ET1]_XP_031083673.1               GIKKNFNAIAFYLDIADMLAKEGY.....................................
[Fusarium_fujikuroi]_SCN78580.1                          GIKKNFNAIAFYLDIADMLAKEGY.....................................
[Fusarium_fujikuroi_IMI_58289]_XP_023430709.1            GIKKNFNAIAFYLDIADMLAKEGY.....................................
[Fusarium_sp._NRRL_25303]_KAF5626228.1                   GIKKNFNAIAFYLDIADMLAKEATLPRTLDENPPSSTFVPRDPVIALAVDKRGIAEGRNR
[Fusarium_xylarioides]_KAG5746670.1                      GIKKNFNAIAFYLDIADMLAKEGY.....................................
[Fusarium_xylarioides]_KAG5788219.1                      GIKKNFNAIAFYLDIADMLAKEGY.....................................
[Fusarium_pseudocircinatum]_KAF5575212.1                 GIKKNFNAIAFYLDIADMLAKEATLPRTLDENPPSSTFVPRDPVIALAVDKR........
[Fusarium_pseudoanthophilum]_KAF5575021.1                GIKKNFNAIAFYLDIADMLAKEATLPRTLDENPPSSTFAPRDPVIALAVDKRGIGEGRNR
[Fusarium_verticillioides]_RBQ71412.1                    GIKKNFNAIAFYLDIADMLAKEGY.....................................
[Fusarium_verticillioides_7600]_XP_018746479.1           GIKKNFNAIAFYLDIADMLAKEGY.....................................
[Fusarium_oxysporum_NRRL_32931]_XP_031039788.1           GIKRNFNAIAFYLDIADMLAKEGY.....................................
[Fusarium_oxysporum_f._sp._lycopersici_4287]_XP_0        GIKRNFNAIAFYLDIADMLAKEGY.....................................
[Fusarium_oxysporum_f._sp._cepae]_RKK20771.1             GIKRNFNAIAFYLDIADMLAKEGY.....................................
[Fusarium_redolens]_XP_046051341.1                       GIKKKFNAIAFYLDIADMLAKEGY.....................................
[Fusarium_austroafricanum]_KAF4458007.1                  GIKKNFNAIAFYLDIADMLAKEGY.....................................
[Fusarium_flagelliforme]_XP_045986929.1                  GIKKNFNAIAFYLDIADMLAKEGF.....................................
[Fusarium_sp._FIESC_RH6]_KAI1056250.1                    GIKKNFNAIAFYLDIADMLAKEGF.....................................
[Fusarium_sporotrichioides]_RGP72309.1                   GIKKNFNAIAFYLDIADMLAKEGF.....................................
[Fusarium_poae]_XP_044713592.1                           GIKKNFNAIAFYLDIADMLAKEGF.....................................
[Fusarium_venenatum]_XP_025590637.1                      GIKKNFNAIAFYLDIADMLAKEGF.....................................
[Fusarium_pseudograminearum]_KAF0641638.1                GIKKNFNAIAFYLDIADMLAKEGF.....................................
[Fusarium_graminearum]_CAG1993854.1                      GIKKNFNAIAFYLDIADMLAKEGF.....................................
[Fusarium_graminearum]_PCD31638.1                        GIKKNFNAIAFYLDIADMLAKEGF.....................................
[Fusarium_graminearum]_CZS78629.1                        GIKKNFNAIAFYLDIADMLAKEGF.....................................
[Fusarium_graminearum]_CAF3606094.1                      GIKKNFNAIAFYLDIADMLAKEGF.....................................
[Fusarium_graminearum_PH-1]_XP_011318944.1               GIKKNFNAIAFYLDIADMLAKEGF.....................................
[Fusarium_culmorum]_QPC68571.1                           GIKKNFNAIAFYLDIADMLAKEGF.....................................
[Fusarium_pseudograminearum]_QPC77441.1                  GIKKNFNAIAFYLDIADMLAKEGF.....................................
[Fusarium_longipes]_RGP79158.1                           GIKKNFNAIAFYLDIADMLAKEGF.....................................
[Ilyonectria_destructans]_KAH7011941.1                   GMKRTFNAIAFYLDMADMLAKEGL.....................................
[Ilyonectria_sp._MPI-CAGE-AT-0026]_KAH6985660.1          GMKRTFNAIAFYLDMADMLAKEGL.....................................
[Ilyonectria_robusta]_XP_046104990.1                     GMKRTFNAIAFYLDMADMLAKEGL.....................................
[Dactylonectria_estremocensis]_KAH7149692.1              GMKRNFNAIAFYLDMADMLAKEDL.....................................
[Dactylonectria_macrodidyma]_KAH7148923.1                GMKRNFNAIAFYLDMADMLAKEGL.....................................
[Thelonectria_olida]_KAH6899778.1                        GMKRNFNAIAFYLDIADMLAKEGL.....................................
[Stachybotrys_chartarum_IBT_7711]_KEY69997.1             GMKKTFNAVAFYLDIADMLAKEGL.....................................
[Stachybotrys_chartarum_IBT_40293]_KFA50745.1            GMKKTFNAVAFYLDIADMLAKEGL.....................................
[Stachybotrys_chlorohalonata_IBT_40285]_KFA62815.        GMKKTFNAVAFYLDIADMLAKEGL.....................................
[Purpureocillium_lilacinum]_XP_018174914.1               GIKRNFNAVAFYLDIADMLAKEGY.....................................
[Clonostachys_solani]_CAH0059244.1                       GVKRTFNAIAFYLDIADMLAKEGL.....................................
[Clonostachys_rosea]_VUC36940.1                          GVKRTFNAIAFYLDIADMLAKEGL.....................................
[Clonostachys_rhizophaga]_CAH0026503.1                   GVKRTFNAIAFYLDIADMLAKEGL.....................................
[Emericellopsis_atlantica]_XP_046114137.1                GMKRTFNAIAFYLDVADMLAKEGL.....................................
[Acremonium_chrysogenum_ATCC_11550]_KFH46845.1           GMKRNFNAIAFYLDIADMLAKEGY.....................................
[Pochonia_chlamydosporia_170]_XP_018148143.1             GVRKRFNAVAFYLGIADMLAREGL.....................................
[Epichloe_festucae_Fl1]_QPG98063.1                       GVRRGFNAVAFYLGIADMLAKEGL.....................................
[Colletotrichum_incanum]_KZL82863.1                      GQARTFNAVAFYLDIADMLAKEGL.....................................
[Colletotrichum_simmondsii]_KXH39849.1                   GQTRTFNAVAFYLDIADMLAKEGL.....................................
[Colletotrichum_sojae]_KAF6819546.1                      GQTRTFNAVAFYLDIADMLAKEGL.....................................
```

[Trichoderma_harzianum]_ThaL

```
[Trichoderma_harzianum]_ThaL                               ...........................
[Trichoderma_harzianum]_KKO99473.1                         ...........................
[Trichoderma_guizhouense]_OPB43634.1                       ...........................
[Trichoderma_simmonsii]_QYS94233.1                         ...........................
[Trichoderma_harzianum_CBS_226.95]_XP_024777038.1          ...........................
[Trichoderma_harzianum]_PKK42226.1                         ...........................
[Trichoderma_lentiforme]_KAF3077685.1_                     ...........................
[Trichoderma_harzianum]_PNP59423.1                         ...........................
[Trichoderma_virens_FT-333]_UKZ73321.1                     ...........................
[Trichoderma_virens_Gv29-8]_XP_013960763.1                 ...........................
[Trichoderma_asperellum_CBS_433.97]_XP_024756506.          ...........................
[Trichoderma_atroviride_IMI_206040]_XP_013943551.          ...........................
[Trichoderma_gamsii]_PNP38305.1                            ...........................
[Trichoderma_gamsii]_XP_018663207.1                        ...........................
[Trichoderma_arundinaceum]_RFU75476.1                      ...........................
[Trichoderma_gracile]_KAH0498535.1                         ...........................
[Trichoderma_reesei_QM6a]_XP_006968245.1                   ...........................
[Trichoderma_citrinoviride]_XP_024753310.1                 ...........................
[Trichoderma_longibrachiatum_ATCC_18648]_PTB78011          ...........................
[Hirsutella_minnesotensis_3608]_KJZ78693.1                 ...........................
[Hirsutella_rhossiliensis]_XP_044725020.1                  ...........................
[Ophiocordyceps_sinensis_CO18]_EQK99704.1                  ...........................
[Tolypocladium_capitatum]_PNY29418.1                       ...........................
[Tolypocladium_ophioglossoides_CBS_100239]_KND888          ...........................
[Tolypocladium_paradoxum]_POR38551.1                       ...........................
[Drechmeria_coniospora]_ODA83100.1                         ...........................
[Drechmeria_coniospora]_XP_040657415.1                     ...........................
[Ophiocordyceps_unilateralis]_PFH57814.1                   ...........................
[Fusarium_sp._AF-6]_RSL54391.1                             ...........................
[Fusarium_kuroshium]_RMJ16314.1                            ...........................
[Fusarium_sp._AF-8]_RSL70798.1                             ...........................
[Fusarium_ambrosium]_RSM11997.1                            ...........................
[Fusarium_floridanum]_RSL80024.1                           ...........................
[Fusarium_vanettenii_77-13-4]_XP_003050716.1               ...........................
[Fusarium_solani]_XP_046134414.1                           ...........................
[Fusarium_albosuccineum]_KAF4468415.1                      ...........................
[Fusarium_decemcellulare]_KAF5011412.1                     ...........................
[Fusarium_sarcochroum]_KAF4964785.1                        ...........................
[Fusarium_avenaceum]_KAH6968652.1                          ...........................
[Fusarium_avenaceum]_KIL96274.1                            ...........................
[Fusarium_avenaceum]_KAG5656265.1                          ...........................
[Fusarium_tricinctum]_KAH7262197.1                         ...........................
[Fusarium_heterosporum]_KAF5663380.1                       ...........................
[Fusarium_sp._MPI-SDFR-AT-0072]_KAH7164412.1               ...........................
[Fusarium_oxysporum_f._sp._rapae]_KAG7414252.1             ...........................
[Fusarium_mangiferae]_XP_041681833.1                       ...........................
[Fusarium_proliferatum]_CVL11341.1                         ...........................
[Fusarium_proliferatum_ET1]_XP_031083673.1                 ...........................
[Fusarium_fujikuroi]_SCN78580.1                            ...........................
[Fusarium_fujikuroi_IMI_58289]_XP_023430709.1              ...........................
[Fusarium_sp._NRRL_25303]_KAF5626228.1                     QQLTGIRGHSRSPDVSQKAPRRNTTST
[Fusarium_xylarioides]_KAG5746670.1                        ...........................
[Fusarium_xylarioides]_KAG5788219.1                        ...........................
[Fusarium_pseudocircinatum]_KAF5575212.1                   ...........................
[Fusarium_pseudoanthophilum]_KAF5575021.1                  QHLTGIRGHSRSPDVSQKAPRRNTTST
[Fusarium_verticillioides]_RBQ71412.1                      ...........................
[Fusarium_verticillioides_7600]_XP_018746479.1             ...........................
[Fusarium_oxysporum_NRRL_32931]_XP_031039788.1             ...........................
[Fusarium_oxysporum_f._sp._lycopersici_4287]_XP_0          ...........................
[Fusarium_oxysporum_f._sp._cepae]_RKK20771.1               ...........................
[Fusarium_redolens]_XP_046051341.1                         ...........................
[Fusarium_austroafricanum]_KAF4458007.1                    ...........................
[Fusarium_flagelliforme]_XP_045986929.1                    ...........................
[Fusarium_sp._FIESC_RH6]_KAI1056250.1                      ...........................
[Fusarium_sporotrichioides]_RGP72309.1                     ...........................
[Fusarium_poae]_XP_044713592.1                             ...........................
[Fusarium_venenatum]_XP_025590637.1                        ...........................
[Fusarium_pseudograminearum]_KAF0641638.1                  ...........................
[Fusarium_graminearum]_CAG1993854.1                        ...........................
[Fusarium_graminearum]_PCD31638.1                          ...........................
[Fusarium_graminearum]_CZS78629.1                          ...........................
[Fusarium_graminearum]_CAF3606094.1                        ...........................
[Fusarium_graminearum_PH-1]_XP_011318944.1                 ...........................
[Fusarium_culmorum]_QPC68571.1                             ...........................
[Fusarium_pseudograminearum]_QPC77441.1                    ...........................
[Fusarium_longipes]_RGP79158.1                             ...........................
[Ilyonectria_destructans]_KAH7011941.1                     ...........................
[Ilyonectria_sp._MPI-CAGE-AT-0026]_KAH6985660.1            ...........................
[Ilyonectria_robusta]_XP_046104990.1                       ...........................
[Dactylonectria_estremocensis]_KAH7149692.1                ...........................
[Dactylonectria_macrodidyma]_KAH7148923.1                  ...........................
[Thelonectria_olida]_KAH6899778.1                          ...........................
[Stachybotrys_chartarum_IBT_7711]_KEY69997.1               ...........................
[Stachybotrys_chartarum_IBT_40293]_KFA50745.1              ...........................
[Stachybotrys_chlorohalonata_IBT_40285]_KFA62815.          ...........................
[Purpureocillium_lilacinum]_XP_018174914.1                 ...........................
[Clonostachys_solani]_CAH0059244.1                         ...........................
[Clonostachys_rosea]_VUC36940.1                            ...........................
[Clonostachys_rhizophaga]_CAH0026503.1                     ...........................
[Emericellopsis_atlantica]_XP_046114137.1                  ...........................
[Acremonium_chrysogenum_ATCC_11550]_KFH46845.1             ...........................
[Pochonia_chlamydosporia_170]_XP_018148143.1               ...........................
[Epichloe_festucae_Fl1]_QPG98063.1                         ...........................
[Colletotrichum_incanum]_KZL82863.1                        ...........................
[Colletotrichum_simmondsii]_KXH39849.1                     ...........................
[Colletotrichum_sojae]_KAF6819546.1                        ...........................
```
