# Supplementary material for: Identification of a Novel Lipase with AHSMG Pentapeptide in Hypocreales and Glomerellales Filamentous Fungi
Source: Int J Mol Sci. 2022 Aug 19;23(16):9367. doi: 10.3390/ijms23169367 (PMC9408867; doi:10.3390/ijms23169367)
Supplement: Supplementary file 1 [file ijms-23-09367-s001.zip › Figure S3B.Multialignment of ThaL and its homologs, with TaLipA, RN2 and 2Z5G.pdf]

2Z5G_1|Chains

```
2Z5G_1|Chains  ............................................................
[Trichoderma_harzianum]_ThaL  ..........................................................MK
[Trichoderma_harzianum]_KKO99473.1  ..........................................................MK
[Trichoderma_guizhouense]_OPB43634.1  ..........................................................MK
[Trichoderma_simmonsii]_QYS94233.1  ..........................................................MK
[Trichoderma_harzianum_CBS_226.95]_XP_024777038.1  ..........................................................MK
[Trichoderma_harzianum]_PKK42226.1  ..........................................................MK
[Trichoderma_lentiforme]_KAF3077685.1_  ..........................................................MK
[Trichoderma_harzianum]_PNP59423.1  ..........................................................MT
[Trichoderma_virens_FT-333]_UKZ73321.1  ..........................................................MK
[Trichoderma_virens_Gv29-8]_XP_013960763.1  ..........................................................MK
[Trichoderma_asperellum_CBS_433.97]_XP_024756506.  ..........................................................MR
[Trichoderma_atroviride_IMI_206040]_XP_013943551.  ..........................................................MR
[Trichoderma_gamsii]_PNP38305.1  ..........................................................MR
[Trichoderma_gamsii]_XP_018663207.1  ..........................................................MR
[Trichoderma_arundinaceum]_RFU75476.1  ..........................................................MT
[Trichoderma_gracile]_KAH0498535.1  ..........................................................MK
[Trichoderma_reesei_QM6a]_XP_006968245.1  ..........................................................MK
[Trichoderma_citrinoviride]_XP_024753310.1  ..........................................................MK
[Trichoderma_longibrachiatum_ATCC_18648]_PTB78011  ..........................................................MK
[Hirsutella_minnesotensis_3608]_KJZ78693.1  ..........................................................MS
[Hirsutella_rhossiliensis]_XP_044725020.1  ..........................................................MT
[Ophiocordyceps_sinensis_CO18]_EQK99704.1  ..........................................................MT
[Tolypocladium_capitatum]_PNY29418.1  ..........................................................MS
[Tolypocladium_ophioglossoides_CBS_100239]_KND888  ..........................................................MS
[Tolypocladium_paradoxum]_POR38551.1  ..........................................................MS
[Drechmeria_coniospora]_ODA83100.1  ..........................................................MR
[Drechmeria_coniospora]_XP_040657415.1  ..............MTSVAR....TRIRPQPAHFHFLNVLYRPVLTAPWTHWQPGSRVMR
[Ophiocordyceps_unilateralis]_PFH57814.1  ..........................................................MS
[Fusarium_sp._AF-6]_RSL54391.1  ..........................................................MS
[Fusarium_kuroshium]_RMJ16314.1  ..........................................................MS
[Fusarium_sp._AF-8]_RSL70798.1  ..........................................................MS
[Fusarium_ambrosium]_RSM11997.1  ..........................................................MS
[Fusarium_floridanum]_RSL80024.1  ..........................................................MS
[Fusarium_vanettenii_77-13-4]_XP_003050716.1  ..........................................................MS
[Fusarium_solani]_XP_046134414.1  ..........................................................MS
[Fusarium_albosuccineum]_KAF4468415.1  ..............MRRQTIFIH.........NISFGPCDSRS...............MS
[Fusarium_decemcellulare]_KAF5011412.1  ..............MRRQTIFIN.........NISFGPCDSRS...............MS
[Fusarium_sarcochroum]_KAF4964785.1  ..........................................................MN
[Fusarium_avenaceum]_KAH6968652.1  ..........................................................MN
[Fusarium_avenaceum]_KIL96274.1  ..........................................................MN
[Fusarium_avenaceum]_KAG5656265.1  MVQQIACTRTVTSPISDFVPRC..DIPQPQPSIANCIHATRSS...............MN
[Fusarium_tricinctum]_KAH7262197.1  ..........................................................MN
[Fusarium_heterosporum]_KAF5663380.1  M.......RTVTSPMYRTSSHAT.TVLQPQPSSAHCTHAIQSS...............MN
[Fusarium_sp._MPI-SDFR-AT-0072]_KAH7164412.1  MHSR....RDVT..SSRHSFHLTTRTLPTPTLLSHCAHATQQS...............MN
[Fusarium_oxysporum_f._sp._rapae]_KAG7414252.1  ..........................................................MN
[Fusarium_mangiferae]_XP_041681833.1  ..........................................................MN
[Fusarium_proliferatum]_CVL11341.1  ..........................................................MN
[Fusarium_proliferatum_ET1]_XP_031083673.1  ..........................................................MN
[Fusarium_fujikuroi]_SCN78580.1  ..........................................................MN
[Fusarium_fujikuroi_IMI_58289]_XP_023430709.1  ..........................................................MN
[Fusarium_sp._NRRL_25303]_KAF5626228.1  ..........................................................MN
[Fusarium_xylarioides]_KAG5746670.1  ..........................................................MN
[Fusarium_xylarioides]_KAG5788219.1  ..........................................................MN
[Fusarium_pseudocircinatum]_KAF5575212.1  ............................................................
[Fusarium_pseudoanthophilum]_KAF5575021.1  ..........................................................MN
[Fusarium_verticillioides]_RBQ71412.1  ..........................................................MN
[Fusarium_verticillioides_7600]_XP_018746479.1  ..........................................................MN
[Fusarium_oxysporum_NRRL_32931]_XP_031039788.1  MHSR....RDVI..SSLQSFYLTIRTLPTPTLLSHCAHATQQS...............MN
[Fusarium_oxysporum_f._sp._lycopersici_4287]_XP_0  ..........................................................MN
[Fusarium_oxysporum_f._sp._cepae]_RKK20771.1  ..........................................................MN
[Fusarium_redolens]_XP_046051341.1  ..........................................................MN
[Fusarium_austroafricanum]_KAF4458007.1  ..............MTLDFLHSNATTLNNNASMEFCAYATRQF...............MN
[Fusarium_flagelliforme]_XP_045986929.1  ..........................................................MN
[Fusarium_sp._FIESC_RH6]_KAI1056250.1  .......................................MARS...............MS
[Fusarium_sporotrichioides]_RGP72309.1  ..........................MSPQHSIVSSTHAIRKF...............MN
[Fusarium_poae]_XP_044713592.1  ..........................................................MN
[Fusarium_venenatum]_XP_025590637.1  ..........................................................MN
[Fusarium_pseudograminearum]_KAF0641638.1  ..........................MSPQHSIVSSTHAIQKS...............MN
[Fusarium_graminearum]_CAG1993854.1  ..........................MSPQHSIVSSTHAIQKS...............MN
[Fusarium_graminearum]_PCD31638.1  ..........................MSPQHSIVSSTHAIQKS...............MN
[Fusarium_graminearum]_CZS78629.1  ..........................................................MN
[Fusarium_graminearum]_CAF3606094.1  ..........................MSPQHSIVSSTHAIQKS...............MN
[Fusarium_graminearum_PH-1]_XP_011318944.1  ..........................................................MN
[Fusarium_culmorum]_QPC68571.1  ..........................MSPQHSIVSSTHAIQKS...............MN
[Fusarium_pseudograminearum]_QPC77441.1  ..........................MSPQHSIVSYTHAIQKS...............MN
[Fusarium_longipes]_RGP79158.1  ..........................MSPQLPIASFTHAVQKS...............MN
[Ilyonectria_destructans]_KAH7011941.1  ..........................................................MS
[Ilyonectria_sp._MPI-CAGE-AT-0026]_KAH6985660.1  ..........................................................MS
[Ilyonectria_robusta]_XP_046104990.1  ..........................................................MS
[Dactylonectria_estremocensis]_KAH7149692.1  ..........................................................MS
[Dactylonectria_macrodidyma]_KAH7148923.1  ..........................................................MS
[Thelonectria_olida]_KAH6899778.1  ..........................................................MT
[Stachybotrys_chartarum_IBT_7711]_KEY69997.1  ..........................................................MN
[Stachybotrys_chartarum_IBT_40293]_KFA50745.1  ..........................................................MN
[Stachybotrys_chlorohalonata_IBT_40285]_KFA62815.  ..........................................................MN
[Purpureocillium_lilacinum]_XP_018174914.1  ..............M...........................................IT
[Clonostachys_solani]_CAH0059244.1  ..........................................................MS
[Clonostachys_rosea]_VUC36940.1  ..........................................................MS
[Clonostachys_rhizophaga]_CAH0026503.1  ..........................................................MK
[Emericellopsis_atlantica]_XP_046114137.1  ..........................................................MR
[Acremonium_chrysogenum_ATCC_11550]_KFH46845.1  ..........................................................MS
[Pochonia_chlamydosporia_170]_XP_018148143.1  ..........................................................MS
[Epichloe_festucae_Fl1]_QPG98063.1  ..........................................................MR
[Colletotrichum_incanum]_KZL82863.1  ..............MNSAC.......................................LT
[Colletotrichum_simmondsii]_KXH39849.1  ..............MNNAC.......................................LS
[Colletotrichum_sojae]_KAF6819546.1  ..............MNTAC.......................................LA
[Bacillus_licheniformis]_CAB95850.2_RN2  ..............MS................LL........................MK
[Trichosporon_asahii_var._asahii]_AGN98126.1_TaLi  ..........................................................MR
```

2Z5G_1|Chains

```
2Z5G_1|Chains                                        ............................................................
[Trichoderma_harzianum]_ThaL                         Q.............WPH.WRGTRLPR..................SLLSLQ..........
[Trichoderma_harzianum]_KKO99473.1                   Q.............WPH.WRGTRLPR..................SLLSLQ..........
[Trichoderma_guizhouense]_OPB43634.1                 Q.............WPH.WRGTRLPR..................SLLSLQ..........
[Trichoderma_simmonsii]_QYS94233.1                   Q.............WPH.WRGTRLPR..................SLLSLQ..........
[Trichoderma_harzianum_CBS_226.95]_XP_024777038.1    Q.............WPH.WRGTRLPR..................SLLSLQ..........
[Trichoderma_harzianum]_PKK42226.1                   Q.............WPH.WRGTRLPR..................SLLSLQ..........
[Trichoderma_lentiforme]_KAF3077685.1_               Q.............WPH.WRGTRLPR..................SLLKLQ..........
[Trichoderma_harzianum]_PNP59423.1                   T...........................................................
[Trichoderma_virens_FT-333]_UKZ73321.1               Q.............WRH.WRSSRLRA..................SLLNLR..........
[Trichoderma_virens_Gv29-8]_XP_013960763.1           Q.............WRH.WRRSRLRV..................SLPNLR..........
[Trichoderma_asperellum_CBS_433.97]_XP_024756506.    K.............WLL.HRGPRLSR..................PPPNLR..........
[Trichoderma_atroviride_IMI_206040]_XP_013943551.    K.............WLLHHRGPRLSR..................PLPNLR..........
[Trichoderma_gamsii]_PNP38305.1                      K.............WLLHYRGPRLSP.................LPPPNLR..........
[Trichoderma_gamsii]_XP_018663207.1                  K.............WLLHYRGPRLSP.................LPPPNLR..........
[Trichoderma_arundinaceum]_RFU75476.1                K.............WHT.YRGPRLSR..................SLPNLR..........
[Trichoderma_gracile]_KAH0498535.1                   Q.............WPR.HHGPRLSR..................SLPNLR..........
[Trichoderma_reesei_QM6a]_XP_006968245.1             Q.............WPR.HHGPRLSR..................SLPNLR..........
[Trichoderma_citrinoviride]_XP_024753310.1           Q.............WPR.HHGPRLSR..................SLLNLR..........
[Trichoderma_longibrachiatum_ATCC_18648]_PTB78011    Q.............WPR.QYGPRLTR..................SPPTLR..........
[Hirsutella_minnesotensis_3608]_KJZ78693.1           T.............CSPRAPAVSIHR.......AAA.AAARQSCRRPSCR..........
[Hirsutella_rhossiliensis]_XP_044725020.1            ............................................................
[Ophiocordyceps_sinensis_CO18]_EQK99704.1            NVGNSLAS......VHLRIPCKAICQRR...MHSKT......TLLRSACR..........
[Tolypocladium_capitatum]_PNY29418.1                 R.............WAPPHNILTLAGL..........RPQCRHLPRPSCR..........
[Tolypocladium_ophioglossoides_CBS_100239]_KND888    R.............WLPRYSISTLAGR..........QPQCQHLPHLSCR..........
[Tolypocladium_paradoxum]_POR38551.1                 R.............WLPRCNTPILAGR..........RPRRQHLPRPSCR..........
[Drechmeria_coniospora]_ODA83100.1                   NIP...........WLTRYYSPPSLAAGRL.......RPH....STPACR..........
[Drechmeria_coniospora]_XP_040657415.1               NIP...........WLTRYYSPPSLAAGRL.......RPH....STPACR..........
[Ophiocordyceps_unilateralis]_PFH57814.1             R............................KCIIGSC......LFARLPCA......SWP.
[Fusarium_sp._AF-6]_RSL54391.1                       R.............SAPLRRALILAS..................PRPSLS.....IQWL.
[Fusarium_kuroshium]_RMJ16314.1                      R.............SAPLRRALILAS..................PRPSSS.....IQWL.
[Fusarium_sp._AF-8]_RSL70798.1                       R.............SAPLRRALILAS..................PRPSSS.....TQWL.
[Fusarium_ambrosium]_RSM11997.1                      R.............SAPLRRALILAS..................PRPSSS.....IQWL.
[Fusarium_floridanum]_RSL80024.1                     R.............SAPLRRALILAS..................PRPSSS.....TQWL.
[Fusarium_vanettenii_77-13-4]_XP_003050716.1         R.............SALLRRALILAS..................PRPSSS.....IQWL.
[Fusarium_solani]_XP_046134414.1                     K.............SALLRRALTLAS..................PRLSSS.....TQWL.
[Fusarium_albosuccineum]_KAF4468415.1                R.............STLWRRTPVLAL..................KNPPCR.....AQWH.
[Fusarium_decemcellulare]_KAF5011412.1               R.............STLWRRTPVLAL..................KNPPCR.....AQWH.
[Fusarium_sarcochroum]_KAF4964785.1                  R.............SIIWRRAPTSAL..................TKEPCR.....AQWS.
[Fusarium_avenaceum]_KAH6968652.1                    R.............STIWRKAPAFPL..................ARHTCR.....VQWH.
[Fusarium_avenaceum]_KIL96274.1                      R.............STIWRKAPAFPL..................ARHTCR.....VQWH.
[Fusarium_avenaceum]_KAG5656265.1                    R.............STIWRKAPAFPL..................ARHTCR.....AQWH.
[Fusarium_tricinctum]_KAH7262197.1                   R.............SKIWRKAPAFPR..................ARHTCH.....AQWH.
[Fusarium_heterosporum]_KAF5663380.1                 S.............LIIWRKAPAPPL..................ARHSCR.....AQWH.
[Fusarium_sp._MPI-SDFR-AT-0072]_KAH7164412.1         R.............SAIWRNAPASPL..................FRQLYR.....AQCH.
[Fusarium_oxysporum_f._sp._rapae]_KAG7414252.1       R.............SAIWRNAPASPL..................FRQLYR.....AQCH.
[Fusarium_mangiferae]_XP_041681833.1                 R.............SVIWRNAPDLLL..................SRQLYR.....AQWH.
[Fusarium_proliferatum]_CVL11341.1                   R.............SVIWRNAPDLLL..................SRQLYR.....AQWH.
[Fusarium_proliferatum_ET1]_XP_031083673.1           R.............SVIWRNAPDLLL..................SRQLYR.....AQWH.
[Fusarium_fujikuroi]_SCN78580.1                      R.............SVIWRSAPDLLL..................SRQLYR.....AQWH.
[Fusarium_fujikuroi_IMI_58289]_XP_023430709.1        R.............SVIWRNAPDLLL..................SRQLYR.....AQWH.
[Fusarium_sp._NRRL_25303]_KAF5626228.1               R.............SVIWRNAPDLLL..................SRQLYR.....AQWH.
[Fusarium_xylarioides]_KAG5746670.1                  R.............SVIWRNAPTLPL..................SQQLYR.....AQCH.
[Fusarium_xylarioides]_KAG5788219.1                  R.............SVIWRNAPTLPL..................SQQLYR.....AQCH.
[Fusarium_pseudocircinatum]_KAF5575212.1             ................MWRSAPALPL..................SRQLYG.....ARCH.
[Fusarium_pseudoanthophilum]_KAF5575021.1            R.............SVIWRSAPALPL..................SRQLYG.....AQCH.
[Fusarium_verticillioides]_RBQ71412.1                R.............SVIWRSAPALPL..................SRQLYG.....VQCH.
[Fusarium_verticillioides_7600]_XP_018746479.1       R.............SVIWRSAPALPL..................SRQLYG.....AQCH.
[Fusarium_oxysporum_NRRL_32931]_XP_031039788.1       K.............SAIWRNAPASPL..................SRQLYR.....AQCH.
[Fusarium_oxysporum_f._sp._lycopersici_4287]_XP_0    K.............SAIWRNAPASPL..................SRQLYR.....AQCH.
[Fusarium_oxysporum_f._sp._cepae]_RKK20771.1         K.............SAIWRNAPASPL..................SRQLYR.....AQCH.
[Fusarium_redolens]_XP_046051341.1                   R.............SIIRRNAPTSPL..................SRQLYR.....AQCH.
[Fusarium_austroafricanum]_KAF4458007.1              T.............SRTWRKAPASAL..................GRLLYH.....AQWH.
[Fusarium_flagelliforme]_XP_045986929.1              R.............STIWRKAPALSL..................IKYPYR.....KQPF.
[Fusarium_sp._FIESC_RH6]_KAI1056250.1                R.............STIWRKAPALGL..................VRYPYH.....KQPF.
[Fusarium_sporotrichioides]_RGP72309.1               R.............STIWRKAPAPTL..................VKYSYR.....KQPF.
[Fusarium_poae]_XP_044713592.1                       R.............STIWRNAPASTL..................VRYTYR.....KQRP.
[Fusarium_venenatum]_XP_025590637.1                  R.............STIWRKAPASTL..................VRYACR.....KQHP.
[Fusarium_pseudograminearum]_KAF0641638.1            R.............PIIWRNAPAPTL..................VRYTYR.....KQQP.
[Fusarium_graminearum]_CAG1993854.1                  I.............SIIWRKAPVPTL..................VKYPYR.....KQRP.
[Fusarium_graminearum]_PCD31638.1                    I.............SIIWRKAPVPTL..................VKYPYR.....KQRP.
[Fusarium_graminearum]_CZS78629.1                    I.............SIIWRKAPVPTL..................VKYPYR.....KQRP.
[Fusarium_graminearum]_CAF3606094.1                  R.............SIIWRKAPVPTL..................VKYPYR.....KQRP.
[Fusarium_graminearum_PH-1]_XP_011318944.1           R.............SIIWRKAPVPTL..................VKYPYR.....KQRP.
[Fusarium_culmorum]_QPC68571.1                       R.............SIIWRKAPVPTL..................VKYPYR.....KQRP.
[Fusarium_pseudograminearum]_QPC77441.1              R.............SIIWRNAPAPTL..................VRYTYR.....KQQP.
[Fusarium_longipes]_RGP79158.1                       R.............SIIWRKAPVLTP..................PRYPYC.....KQRP.
[Ilyonectria_destructans]_KAH7011941.1               R.............WIVQCKPPAIAV..................TRPTTYLGVTRAQCLL
[Ilyonectria_sp._MPI-CAGE-AT-0026]_KAH6985660.1      R.............WAVRCKPSAIAV..................TSPTTYLGVTRAQCLL
[Ilyonectria_robusta]_XP_046104990.1                 R.............WIVQCKPSAIAV..................TNPTTYLGVTRAQCLL
[Dactylonectria_estremocensis]_KAH7149692.1          R.............RLIRGSIPTIAA..................ARPAARLSVTRAQRFQ
[Dactylonectria_macrodidyma]_KAH7148923.1            R.............RLIRGNVPTIVV..................ARPAARLSVTCAQRSP
[Thelonectria_olida]_KAH6899778.1                    Q.............WLARCRQPVITA..................SGLPSTTITRPVQWLG
[Stachybotrys_chartarum_IBT_7711]_KEY69997.1         K.............WLIRSAKSVLSTAP................SCLVSR......RCPP
[Stachybotrys_chartarum_IBT_40293]_KFA50745.1        K.............WPIRSAKSVLSTAP................SCLVSR......RCPP
[Stachybotrys_chlorohalonata_IBT_40285]_KFA62815.    K.............WPIRSAKSVLSTAP................SCLMSR......YCPP
[Purpureocillium_lilacinum]_XP_018174914.1           R.............WAPRHSTTTTTTAAAAAVTLAVRRPA....IRPHCR.....PSSP.
[Clonostachys_solani]_CAH0059244.1                   S...RLAHHGVRIGFNPRITTTTLKA................NFLLQRQQ..........
[Clonostachys_rosea]_VUC36940.1                      S...RLAHHGVRIGFNPRITTTALNP................NFLLQSQR..........
[Clonostachys_rhizophaga]_CAH0026503.1               K...........................................QCVLIR..........
[Emericellopsis_atlantica]_XP_046114137.1            P..........CRRFHSARSLTGLAQ..................ARHRVR..........
[Acremonium_chrysogenum_ATCC_11550]_KFH46845.1       T.............WLLRRNAPIRRL......NGPASKPR...LPPAATR..........
[Pochonia_chlamydosporia_170]_XP_018148143.1         K.............CLP.RSVPLASL..................RIHHAR..........
[Epichloe_festucae_Fl1]_QPG98063.1                   P.............WLPRRATPLCHE..................AVLSTR..........
[Colletotrichum_incanum]_KZL82863.1                  R.............CRPRAPSPGLIR.......APTA.......SPRLLRPLA.......
[Colletotrichum_simmondsii]_KXH39849.1               R.............CRPCAPSSRLIC.......APTA.......SHRLLRPLA.......
[Colletotrichum_sojae]_KAF6819546.1                  RFSCSSS.....SRHLRRRAIPATFL......SVPTRAPA....ASASLRPLP.......
[Bacillus_licheniformis]_CAB95850.2_RN2              R.............SLQILVAFALVIG.................................
[Trichosporon_asahii_var._asahii]_AGN98126.1_TaLi    L.............SLVTAAAVALSAA.................................
```

2Z5G_1|Chains

```
                                                                  1
                                                                  .
2Z5G_1|Chains                                           .........................................SL................
[Trichoderma_harzianum]_ThaL                            PST.LRVFSTAKPL......RHDPRIRDLGRLIHDDYATIRETY................
[Trichoderma_harzianum]_KKO99473.1                      PST.SRVFSTAKPL......RHDPRIRDLGRLIHDDYATIRETY................
[Trichoderma_guizhouense]_OPB43634.1                    PST.SRVFSTAKPL......RHDPRIRDLGRLIHDDYATIRETY................
[Trichoderma_simmonsii]_QYS94233.1                      PSH.SRVFSTTKPL......RHDPRIRDLGRLIHDDYATIRETY................
[Trichoderma_harzianum_CBS_226.95]_XP_024777038.1       PSA.SRVFSTTKPL......RHDPRIRDLGRLIHDDYATIRETY................
[Trichoderma_harzianum]_PKK42226.1                      PSA.SRVFSTTNPL......RHDPRIRDLGRLIHDDYATIRETY................
[Trichoderma_lentiforme]_KAF3077685.1_                  PSP.SRVFSTAKPLL...SSSHDPRIRDLGRLIHDDYATIRETY................
[Trichoderma_harzianum]_PNP59423.1                      ...........................................................
[Trichoderma_virens_FT-333]_UKZ73321.1                  SSP.SRVLSTARPLL...SSGHDPRIRDLGRRIHDDYATIRETY................
[Trichoderma_virens_Gv29-8]_XP_013960763.1              SSP.SRVLSTARPLL...SSGHDPRIRDLGRRIHDDYATIRETY................
[Trichoderma_asperellum_CBS_433.97]_XP_024756506.       SSS.HQLFSTAKPHL...KSSHDPRIRDFGRRIHDEYATIRENY................
[Trichoderma_atroviride_IMI_206040]_XP_013943551.       SIPWHHFFSTAKAHL...KDSHDPRIRDFGRRIHDDYATIRENY................
[Trichoderma_gamsii]_PNP38305.1                         ..PSHHFFSTAKAYL...KDSNDPRIRDFGRRIHDDYATIRENYGMIESSARLQKEVGTF
[Trichoderma_gamsii]_XP_018663207.1                     ..PSHHFFSTAKAYL...KDSSDPRIRDVGRRIHDDYATIRENYGMIESSARLQKEVGTF
[Trichoderma_arundinaceum]_RFU75476.1                   PSASYRFFSAAKPVL...RPSNDPRIRDLGRRILDDYAIIRDNY................
[Trichoderma_gracile]_KAH0498535.1                      RSR.ARTFSTGKPLL...DDSHDPRIRDLGRQILDDYAIIRENY................
[Trichoderma_reesei_QM6a]_XP_006968245.1                RSR.ARTFSAAKPLL..HESSHDPRIRDLGRQILDDYAMIRENY................
[Trichoderma_citrinoviride]_XP_024753310.1              PSRPRRAFSAGRPFL.EEESSHDPRIRDLGRRILDDYAVIRENY................
[Trichoderma_longibrachiatum_ATCC_18648]_PTB78011       RSR.ARPFSTGKPFL..DESSHDPRIRDLGRQILDDYAVIREKY................
[Hirsutella_minnesotensis_3608]_KJZ78693.1              PLLVRRAFATDAPL....LRDGDARLRHLGREIADEYATIRENY................
[Hirsutella_rhossiliensis]_XP_044725020.1               ....TRALTAGRALR...RDDSDARIRELGREIAHDYATIRESY................
[Ophiocordyceps_sinensis_CO18]_EQK99704.1               .RPTHRASTPARPP....RRHSHGRIRDLGPEIARDYAAIRDSY................
[Tolypocladium_capitatum]_PNY29418.1                    ....RRSLTTSRSH....RRDSDPRIRHLGRRIADEYATIRENYGLRPHGMLTNASSETA
[Tolypocladium_ophioglossoides_CBS_100239]_KND888       .....RRLTTSLSH....RRNSDPRIRDLGRQIADEYAAIREAY................
[Tolypocladium_paradoxum]_POR38551.1                    ....RRSLTTSRPR....RRDSDARMRDLGRQIADEYASIRESY................
[Drechmeria_coniospora]_ODA83100.1                      .LRHGRTLTSSPFL....HHSSDPRVRNLGREISDEYASVRDVY................
[Drechmeria_coniospora]_XP_040657415.1                  .LRHGRTLTSSPFL....HRSSDPRVRNLGREISDEYASVRDVY................
[Ophiocordyceps_unilateralis]_PFH57814.1                SLSNNRPLTTGRAL....RDDNDARMRHPGRPLAQEFADIREKY................
[Fusarium_sp._AF-6]_RSL54391.1                          .SIGSRGFSSGRYL....RRSEDPRIKALGREISDDYATIREKY................
[Fusarium_kuroshium]_RMJ16314.1                         .PIGSRGFSSGRYL....RRSEDPRIKALGREISDDYATIREKY................
[Fusarium_sp._AF-8]_RSL70798.1                          .PIGSRGFSSGRYL....RRSEDPRIKALGREISDDYATIREKY................
[Fusarium_ambrosium]_RSM11997.1                         .PIGSRGFSSGRYL....WRSEDPRIKALGREISDDYATIREKY................
[Fusarium_floridanum]_RSL80024.1                        .SIGSRGFSSGWYL....RRSEDPRIKALGREISDDYATIREKY................
[Fusarium_vanettenii_77-13-4]_XP_003050716.1            .PIVSRGFSSGRYL....RRSEDPRIKALGREISDDYATIREKY................
[Fusarium_solani]_XP_046134414.1                        .PIVSRGFSSGRCL....RRSEDPRIKALGREISDDYATIREKY................
[Fusarium_albosuccineum]_KAF4468415.1                   .PIPRQAFSSGRHL....RRDEDPRIKALGREISDDYATIREKY................
[Fusarium_decemcellulare]_KAF5011412.1                  .PIPRQAFSSGRHL....RRDEDPRIKALGREISDDYATIREKY................
[Fusarium_sarcochroum]_KAF4964785.1                     .PIARRSFTCSSCL....RREHDPRIKALGRQISDDYAIIREKY................
[Fusarium_avenaceum]_KAH6968652.1                       .HVPRRNFTCSSFL....HREHDPRIKALDREISDDYAIIREKY................
[Fusarium_avenaceum]_KIL96274.1                         .HVPRRNFTCSSFL....HREHDPRIKALDREISDDYAIIREKY................
[Fusarium_avenaceum]_KAG5656265.1                       .HVPRRNFTCSSFL....HREHDPRIKALDREISDDYAIIREKY................
[Fusarium_tricinctum]_KAH7262197.1                      .PVPRRNFTCSSFL....HREDDPRIKALDREISDDYAMIREKY................
[Fusarium_heterosporum]_KAF5663380.1                    .TVSRRNFTRSSFL....RREHDPRIKALGREISDDYAIIREKY................
[Fusarium_sp._MPI-SDFR-AT-0072]_KAH7164412.1            .....RSFACSTYL....RREHDPRIKALGREISDDYAIIREKY................
[Fusarium_oxysporum_f._sp._rapae]_KAG7414252.1          .....RSFACSTYL....RREHDPRIKALGREISDDYAIIREKY................
[Fusarium_mangiferae]_XP_041681833.1                    .....RSFACSAYL....TREHDPRIKALGREISDDYATIREKY................
[Fusarium_proliferatum]_CVL11341.1                      .....RSFACSAYL....TREHDPRIKALGREISDDYAIIREKY................
[Fusarium_proliferatum_ET1]_XP_031083673.1              .....RSFACSAYL....TREHDPRIKALGREISDDYAIIREKY................
[Fusarium_fujikuroi]_SCN78580.1                         .....RSFACSAYL....TREHDPRIKALGREISDDYAIIREKY................
[Fusarium_fujikuroi_IMI_58289]_XP_023430709.1           .....RSFACSAYL....TREHDPRIKALGREISDDYAIIREKY................
[Fusarium_sp._NRRL_25303]_KAF5626228.1                  .....RSFACSAYL....TREHDPRIKALGREISDDYATIREKY................
[Fusarium_xylarioides]_KAG5746670.1                     .....RKFACSTYL....GREHDPRIKALGREISDDYAIIREKY................
[Fusarium_xylarioides]_KAG5788219.1                     .....RKFACSTYL....GREHDPRIKALGREISDDYAIIREKY................
[Fusarium_pseudocircinatum]_KAF5575212.1                .....RSFACSTYL....AREHDPRIKALGREISDDYAIIREKY................
[Fusarium_pseudoanthophilum]_KAF5575021.1               .....RSFACSAYL....AREHDPRIKALGREISDDYAIIREKY................
[Fusarium_verticillioides]_RBQ71412.1                   .....RSFACSTYL....AREHDPRIKALGREISDDYAIIREKY................
[Fusarium_verticillioides_7600]_XP_018746479.1          .....RSFACSTYL....AREHDPRIKALGREISDDYAIIREKY................
[Fusarium_oxysporum_NRRL_32931]_XP_031039788.1          .....RSFACSTYL....GREHDPRIKALGREISDDYATIREKY................
[Fusarium_oxysporum_f._sp._lycopersici_4287]_XP_0       .....RSFACSTYL....GREHDPRIKALGREISDDYAIIREKY................
[Fusarium_oxysporum_f._sp._cepae]_RKK20771.1            .....RSFACSTYL....GREHDPRIRALGREISDDYAIIREKY................
[Fusarium_redolens]_XP_046051341.1                      .....RSFACSSYL....GREHDPRIKALGREISDDYAIIREKYGA..............
[Fusarium_austroafricanum]_KAF4458007.1                 HPVSRRTFTRSSYQ....GREHDPRIRALGREISGDYAIIREKY................
[Fusarium_flagelliforme]_XP_045986929.1                 .LLQCRGFTHSHCF....GREHDPRIKALGREISDDYAIIREKY................
[Fusarium_sp._FIESC_RH6]_KAI1056250.1                   .LLQCRSFTHSHCF....GREHDPRIKALGREISDDYAIIREKY................
[Fusarium_sporotrichioides]_RGP72309.1                  .ITQCRSFTYSRCV....ARDYDPRIKAVGRAISDDYALIREKY................
[Fusarium_poae]_XP_044713592.1                          .IVQCRSFTYSRYV....GREYDPRIRAVGRAISDDYALIREKY................
[Fusarium_venenatum]_XP_025590637.1                     .IVQCRSFTYSRYV....GREYDPRIKAVGRAISDDYALIREKY................
[Fusarium_pseudograminearum]_KAF0641638.1               .IVQCRSFTYSRCV....GREFDPRIKAVGRAISDDYALIREKY................
[Fusarium_graminearum]_CAG1993854.1                     .IVQCRSFTYSRCV....GREFDPRIKAVGRAISDDYALIREKY................
[Fusarium_graminearum]_PCD31638.1                       .MVQCRSFTYSRCV....GREFDPRIKAVGRAISDDYALIREKY................
[Fusarium_graminearum]_CZS78629.1                       .MVQCRSFTYSRCV....GREFDPRIKAVGRAISDDYALIREKY................
[Fusarium_graminearum]_CAF3606094.1                     .IVQCRSFTYSRCV....GREFDPRIKAVGRAISDDYALIREKY................
[Fusarium_graminearum_PH-1]_XP_011318944.1              .IVQCRSFTYSRCV....GREFDPRIKAVGRAISDDYALIREKY................
[Fusarium_culmorum]_QPC68571.1                          .IVQCRSFTYSRCV....GREFDPRIKAVGRAISDDYALIREKY................
[Fusarium_pseudograminearum]_QPC77441.1                 .IVQCRSFTYSRCV....GREFDPRIKAVGRAISDDYALIREKYGRFSKSWFLAWGNLTD
[Fusarium_longipes]_RGP79158.1                          .IVQSRSFAYSRCV....GREYDPRIKAVGRAISNDYALIREKY................
[Ilyonectria_destructans]_KAH7011941.1                  RDGHSRSLTTGRPL....RRDGDPRIRPLGRQISDDYATIRDTY................
[Ilyonectria_sp._MPI-CAGE-AT-0026]_KAH6985660.1         RDRHSRSLTTGRPL....RRDGDPRIRPLGRQISDDYATIRDTY................
[Ilyonectria_robusta]_XP_046104990.1                    RDRHSRSLTTGRPL....RRDGDPRIRPLGRQISDDYATIRDTY................
[Dactylonectria_estremocensis]_KAH7149692.1             NGWPARSFSTGRPW....RQEGDPRIRPLGKQISDDYATIRDSY................
[Dactylonectria_macrodidyma]_KAH7148923.1               NGWPARTLSTGHTL....RQERDPRIRPLGKQISDDYATIRDSY................
[Thelonectria_olida]_KAH6899778.1                       KTQNARSFTAGRPL....RQDGDPRVRELGREISDDFATIRDTY................
[Stachybotrys_chartarum_IBT_7711]_KEY69997.1            PLPSRRAFASERP.....RDNSDPRIRDLGRQISDDYAALRDSY................
[Stachybotrys_chartarum_IBT_40293]_KFA50745.1           PLPSRRAFASERP.....RDNSDPRIRDLGRQISDDYAALRDSY................
[Stachybotrys_chlorohalonata_IBT_40285]_KFA62815.       PLPLHRAFTSERP.....RDNSDPRIRDLGRQISDDYAALRDSY................
[Purpureocillium_lilacinum]_XP_018174914.1              LPPRRRLLSTSFPPRREERKAPDPRIRDLGRQIADDYATIRDTY................
[Clonostachys_solani]_CAH0059244.1                      HVGQRRWNS.........SRDVDPRIKALGRTILDDYAAVREEY................
[Clonostachys_rosea]_VUC36940.1                         HVGQRRWNS.........SRDVDPRIKALGRTILDDYAAVREEY................
[Clonostachys_rhizophaga]_CAH0026503.1                  ................EFGRDVDPRIKALGRTILDDYAAVREEYGIFQPHRYDSIRKHQQ
[Emericellopsis_atlantica]_XP_046114137.1               HTPQRRSFASTALR....PQDHDPRIRELGREISDDYAAIRDSY................
[Acremonium_chrysogenum_ATCC_11550]_KFH46845.1          QQPCRTLSSSTYAL....HRDGDPRIRDLGREISDDFATVRDTY................
[Pochonia_chlamydosporia_170]_XP_018148143.1            HPLSHRHISSTSHN....QRSRDPRIRDVGRQIEDDYAAIREHY................
[Epichloe_festucae_Fl1]_QPG98063.1                      LIQCCPLSQTACAR....TRAQDARVRDIGRKIKDDYAYIRDHY................
[Colletotrichum_incanum]_KZL82863.1                     SPGSSRCISASPSQS..KHHDDDPRFRDLGKQISDEYAVVREHY................
[Colletotrichum_simmondsii]_KXH39849.1                  AR.DSRPFSVSPLRP...KHDGDPRFRDLGKQISDEYAVVREHY................
[Colletotrichum_sojae]_KAF6819546.1                     CRASRRCLSTTTPRP...RDDSDPRLRDLGRQISDDFAVVREHY................
[Bacillus_licheniformis]_CAB95850.2_RN2                 SMA................................FIQPKEVK................
[Trichosporon_asahii_var._asahii]_AGN98126.1_TaLi       ATPIERASTSG.....................INDY....DCK................
```

```
                                                          β1             η1                 α1
2Z5G_1|Chains                                            ---->        TT ℓℓℓ             ℓℓℓℓℓℓℓ
                                                           10         20             30        40
2Z5G_1|Chains                                              .....RAN.DAPIVLLHGLTGWGREEMFG.F....KYWGGVRGDIEQWLND..NGYR...
[Trichoderma_harzianum]_ThaL                               .....ATP.KYPIVLAHGLFGFSELRVSP.LLPTIEYWNGIK....QALTA..NNCT...
[Trichoderma_harzianum]_KKO99473.1                         .....ATP.KYPIVLAHGLFGFSELRVSP.LLPTIEYWNGIK....QALTA..NNCT...
[Trichoderma_guizhouense]_OPB43634.1                       .....ATP.KYPIVLAHGLFGFSELRVSP.LLPTIEYWNGIK....QALTA..NNCT...
[Trichoderma_simmonsii]_QYS94233.1                         .....ATP.KYPIVLAHGLFGFSELRVSP.LLPTIEYWNGIK....QALTA..NNCT...
[Trichoderma_harzianum_CBS_226.95]_XP_024777038.1          .....ATP.KYPIVLAHGLFGFSELRVSP.LLPTIEYWNGIK....QALTA..NNCT...
[Trichoderma_harzianum]_PKK42226.1                         .....ATP.KYPIVLAHGLFGFSELRVSP.LLPTIEYWNGIK....QALTA..NNCT...
[Trichoderma_lentiforme]_KAF3077685.1_                     .....ATP.KYPIVLAHGLFGFSELRVSP.LLPTIEYWNGIK....QALTA..NNCT...
[Trichoderma_harzianum]_PNP59423.1                         ..LLSARP.TYPIVLAHGLFGFSELRVSP.LLPPIEYWNGIK....QALTA..NNCT...
[Trichoderma_virens_FT-333]_UKZ73321.1                     .....ATP.KYPIVLAHGLFGFSELKVSP.LLPTIEYWNGIK....QALTA..NNCT...
[Trichoderma_virens_Gv29-8]_XP_013960763.1                 .....ATP.KYPIVLAHGLFGFSELRVSP.LLPTIEYWNGIK....QALTA..NNCT...
[Trichoderma_asperellum_CBS_433.97]_XP_024756506.          .....ATP.KHPIVLAHGLLGFSELRVSA.LLPPIEYWHGIK....QALAQ..NNCT...
[Trichoderma_atroviride_IMI_206040]_XP_013943551.          .....ATP.KHPIVLAHGLLGFSELRVSA.LLPPIEYWHGIK....QALRQ..NNCA...
[Trichoderma_gamsii]_PNP38305.1                            TDTTTATP.KYPIVLAHGLLGFSELRVSA.LLPPIEYWHGIK....QALTQ..NNCA...
[Trichoderma_gamsii]_XP_018663207.1                        TDTTTATP.KYPIVLAHGLLGFSELRVSA.LLPPIEYWHGIK....QALTQ..NNCA...
[Trichoderma_arundinaceum]_RFU75476.1                      .....ATP.KHPIVLAHGLLGFSELRVSA.LLPPIEYWHGIK....QALTE..NNCT...
[Trichoderma_gracile]_KAH0498535.1                         .....ATP.KHPIVLAHGLLGFSELKVSP.YLPAIEYWHGIK....QALVA..NKCS...
[Trichoderma_reesei_QM6a]_XP_006968245.1                   .....ATP.KHPIVLAHGLLGFSELKVSP.YLPAIEYWNGIK....QALVA..NRCS...
[Trichoderma_citrinoviride]_XP_024753310.1                 .....ATP.KHPIVLAHGLLGFSELKVAP.YLPAIEYWHGIK....QALVA..NKCS...
[Trichoderma_longibrachiatum_ATCC_18648]_PTB78011          .....ATP.KHPIVLAHGLLGFSEIKVLP.YLPAIEYWHGIK....QALVA..NKCS...
[Hirsutella_minnesotensis_3608]_KJZ78693.1                 .....ATP.KHPIVLAHGLLGFSELSLVS.ALPPVQYWYGIK....QALTA..QGCPS..
[Hirsutella_rhossiliensis]_XP_044725020.1                  .....ATP.KHPIVLAHGLLGFSELSISS.AFPPLQYWHGIK....QALAA..QGCSN..
[Ophiocordyceps_sinensis_CO18]_EQK99704.1                  .....ATP.KHPIVLAHGLLGFSELSISS.ILPPLQYWHGIK....QALAA..QGCPD..
[Tolypocladium_capitatum]_PNY29418.1                       RLTTAATP.KYPIVLAHGLFGFSELSLSA.ALPPLQYWHGIK....QALTA..QGCSR..
[Tolypocladium_ophioglossoides_CBS_100239]_KND888          .....ATP.KYPIVLAHGLLGFSELSLSA.ALPPLQYWHGIK....QALTA..QGCPR..
[Tolypocladium_paradoxum]_POR38551.1                       .....ATP.KYPIVLAHGLLGFSELSLSA.ALPPLQYWHGIK....QALAA..QGCSR..
[Drechmeria_coniospora]_ODA83100.1                         .....LTP.KHPIVLAHGLLGFAELSISS.LVPPLRYWHGIE....QALAT..QGCPL..
[Drechmeria_coniospora]_XP_040657415.1                     .....LTP.KHPIVLAHGLLGFAELSISS.LVPPLRYWHGIE....QALAT..QGCPL..
[Ophiocordyceps_unilateralis]_PFH57814.1                   .....ATP.KHPIVLAHGLLGFSELSLLPALVPPVQYWHGIK....QALVA..QGCPA..
[Fusarium_sp._AF-6]_RSL54391.1                             .....KTP.KYPIVLAHGLFGFAELRLAS.YLPAVEYWHGIR....DALTA..QGAT...
[Fusarium_kuroshium]_RMJ16314.1                            .....KTP.KYPIVLAHGLFGFAELRLAS.YLPAVEYWHGIR....DALTA..QGAT...
[Fusarium_sp._AF-8]_RSL70798.1                             .....KTP.KYPIVLAHGLFGFAELRLAS.YLPAVEYWHGIR....DALTA..QGAT...
[Fusarium_ambrosium]_RSM11997.1                            .....KTP.KYPIVLAHGLFGFAELRLAS.YLPAVEYWHGIR....DALTA..QGAT...
[Fusarium_floridanum]_RSL80024.1                           .....KTP.KYPIVLAHGLFGFAELRLAS.YLPAVEYWHGIR....DALTA..QGAT...
[Fusarium_vanettenii_77-13-4]_XP_003050716.1               .....KTP.KYPIVLAHGLFGFAELRLAS.YLPAVEYWHGIR....DALTA..QGAT...
[Fusarium_solani]_XP_046134414.1                           .....KTP.KYPIVLAHGLFGFAELRLAS.YLPAVEYWHGIR....DALTA..QGAT...
[Fusarium_albosuccineum]_KAF4468415.1                      .....STP.KYPIVLAHGLFGFAELRLGS.YLPAVQYWHGIR....DALTA..QGAT...
[Fusarium_decemcellulare]_KAF5011412.1                     .....STP.KYPIVLAHGLFGFAELRLGS.YLPAVQYWHGIR....DALTA..QGAT...
[Fusarium_sarcochroum]_KAF4964785.1                        .....ETP.KYPIVLAHGLFGFAELRFSP.YLPAVEYWHGIR....DALTA..QGAT...
[Fusarium_avenaceum]_KAH6968652.1                          .....DTP.KYPIVLAHGLFGFAELRLSP.YFPAVEYWHGIR....DALTA..QGAT...
[Fusarium_avenaceum]_KIL96274.1                            .....DTP.KYPIVLAHGLFGFAELRLSP.YFPAVEYWHGIR....DALTA..QGAT...
[Fusarium_avenaceum]_KAG5656265.1                          .....DTP.KYPIVLAHGLFGFAELRLSP.YFPAVEYWHGIR....DALTA..QGAT...
[Fusarium_tricinctum]_KAH7262197.1                         .....DTP.KYPIVLAHGLFGFAELRLSP.YFPAVEYWHGIR....DALTA..QGAT...
[Fusarium_heterosporum]_KAF5663380.1                       .....ETP.KYPIVLAHGLFGFAELRLSS.YFPAVEYWHGIR....DALTA..QGAT...
[Fusarium_sp._MPI-SDFR-AT-0072]_KAH7164412.1               .....ETP.KYPIVLAHGLFGFAELRLAP.YFPAVEYWHGIR....DALTA..QGAT...
[Fusarium_oxysporum_f._sp._rapae]_KAG7414252.1             .....ETP.KYPIVLAHGLFGFAELRLAP.YFPAVEYWHGIR....DALTA..QGAT...
[Fusarium_mangiferae]_XP_041681833.1                       .....ETP.KYPIVLAHGLFGFAELRLSP.YFPAVEYWHGIR....DALTA..QGAT...
[Fusarium_proliferatum]_CVL11341.1                         .....ETP.KYPIVLAHGLFGFAELRLSP.YFPAVEYWHGIR....DALTA..QGAT...
[Fusarium_proliferatum_ET1]_XP_031083673.1                 .....ETP.KYPIVLAHGLFGFAELRLSP.YFPAVEYWHGIR....DALTA..QGAT...
[Fusarium_fujikuroi]_SCN78580.1                            .....ETP.KYPIVLAHGLFGFAELRLSP.YFPAVEYWHGIH....DALTA..QGAT...
[Fusarium_fujikuroi_IMI_58289]_XP_023430709.1              .....ETP.KYPIVLAHGLFGFAELRLSP.YFPAVEYWHGIH....DALTA..QGAT...
[Fusarium_sp._NRRL_25303]_KAF5626228.1                     .....ETP.KYPIVLAHGLFGFAELRLSP.YFPAVEYWHGIR....DALTA..QGAT...
[Fusarium_xylarioides]_KAG5746670.1                        .....ETP.KYPIVLAHGLFGFAELRLSP.YFPAVEYWHGIR....DALTA..QGAT...
[Fusarium_xylarioides]_KAG5788219.1                        .....ETP.KYPIVLAHGLFGFAELRLSP.YFPAVEYWHGIR....DALTA..QGAT...
[Fusarium_pseudocircinatum]_KAF5575212.1                   .....ETP.KYPIVLAHGLFGFAELRLSP.YFPAVEYWHGIR....DALTA..QGAT...
[Fusarium_pseudoanthophilum]_KAF5575021.1                  .....ETP.KYPIVLAHGLFGFAELRLSP.YFPAVEYWHGIR....DALTA..QGAT...
[Fusarium_verticillioides]_RBQ71412.1                      .....ETP.KYPIVLAHGLFGFAELRLSP.YFPAVEYWHGIR....DALTA..QGAT...
[Fusarium_verticillioides_7600]_XP_018746479.1             .....ETP.KYPIVLAHGLFGFAELRLSP.YFPAVEYWHGIR....DALTA..QGAT...
[Fusarium_oxysporum_NRRL_32931]_XP_031039788.1             .....ETP.KYPIVLAHGLFGFAELRLSP.YFPAVEYWHGIR....EALTA..QGAT...
[Fusarium_oxysporum_f._sp._lycopersici_4287]_XP_0          .....ETP.KYPIVLAHGLFGFAELRLSP.YFPAVEYWHGIR....DALTA..QGAT...
[Fusarium_oxysporum_f._sp._cepae]_RKK20771.1               .....ETP.KYPIVLAHGLFGFAELRLSP.YFPAVEYWHGIR....DALTA..QGAT...
[Fusarium_redolens]_XP_046051341.1                         .AFHPTIP.KYPIVLAHGLFGFAELRLSP.YFPAVEYWHGIR....DALTA..QGAT...
[Fusarium_austroafricanum]_KAF4458007.1                    .....EIP.KYPIVLAHGLFGFAEIRLSP.YFPAVEYWHGIR....DALTA..QGAT...
[Fusarium_flagelliforme]_XP_045986929.1                    .....ETP.KYPIVLAHGLFGFAELRLSP.YFPAVEYWHGIR....DALTA..QGAS...
[Fusarium_sp._FIESC_RH6]_KAI1056250.1                      .....ETP.KYPIVLAHGLFGFAELRLSP.YFPAVEYWHGIR....DALAA..QGAS...
[Fusarium_sporotrichioides]_RGP72309.1                     .....DTP.KYPIVLAHGLFGFSELSLSP.YFPSIEYWHGIR....DALTV..QGAT...
[Fusarium_poae]_XP_044713592.1                             .....DTP.KYPIVLAHGLFGFAELSLSP.YFPSVEYWHGIR....DALTA..QGVT...
[Fusarium_venenatum]_XP_025590637.1                        .....DTP.KYPIVLAHGLFGFAELSLSP.YFPSVEYWHGIR....DALTA..QGVT...
[Fusarium_pseudograminearum]_KAF0641638.1                  .....DTP.KYPIVLAHGLFGFAELSLSP.YFPSVEYWHGIR....DALTA..QGAT...
[Fusarium_graminearum]_CAG1993854.1                        .....DTP.KYPIVLAHGLFGFAELSLSP.YFPSVEYWHGIR....DALTA..QGAT...
[Fusarium_graminearum]_PCD31638.1                          .....DTP.KYPIVLAHGLFGFAELSLSP.YFPSVEYWHGIR....DALTA..QGAT...
[Fusarium_graminearum]_CZS78629.1                          .....DTP.KYPIVLAHGLFGFAELSLSP.YFPSVEYWHGIR....DALTA..QGAT...
[Fusarium_graminearum]_CAF3606094.1                        .....DTP.KYPIVLAHGLFGFAELSLSP.YFPSVEYWHGIR....DALTA..QGAT...
[Fusarium_graminearum_PH-1]_XP_011318944.1                 .....DTP.KYPIVLAHGLFGFAELSLSP.YFPSVEYWHGIR....DALTA..QGAT...
[Fusarium_culmorum]_QPC68571.1                             .....DTP.KYPIVLAHGLFGFAELSLSP.YFPSVEYWHGIR....DALTA..QGAT...
[Fusarium_pseudograminearum]_QPC77441.1                    .NHATDTP.KYPIVLAHGLFGFAELSLSP.YFPSVEYWHGIR....DALTA..QGAT...
[Fusarium_longipes]_RGP79158.1                             .....DTP.KYPIVLAHGLFGFAELSLSP.YFPSVEYWHGIR....DALTA..QGAT...
[Ilyonectria_destructans]_KAH7011941.1                     .....DTP.KYPIVLAHGLLGFSELQLSS.YLPSVQYWHGIK....EALTA..QGAT...
[Ilyonectria_sp._MPI-CAGE-AT-0026]_KAH6985660.1            .....DTP.KYPIVLAHGLLGFSELQLSS.YLPSVQYWHGIK....EALTA..QGAI...
[Ilyonectria_robusta]_XP_046104990.1                       .....DTP.KYPIVLAHGLLGFSELQLSS.YLPSVQYWHGIK....EALTA..QGAI...
[Dactylonectria_estremocensis]_KAH7149692.1                .....ETP.KYPIVLAHGLLGFAEFQLAS.YLPAVRYWHGIK....EALTA..QGAT...
[Dactylonectria_macrodidyma]_KAH7148923.1                  .....ETP.KYPIVLAHGLLGFAEFQLSS.YLPTVHYWHGIK....EALTA..QGAT...
[Thelonectria_olida]_KAH6899778.1                          .....ETP.KYPIVLAHGLFGFSELRLHS.SLPAVYYWHGIK....EALSA..QGAV...
[Stachybotrys_chartarum_IBT_7711]_KEY69997.1               .....ASP.KYPVVLAHGLLGFSELKVLE.MLPAVQYWYGIK....DALTE..QGST...
[Stachybotrys_chartarum_IBT_40293]_KFA50745.1              .....ASP.KYPVVLAHGLLGFSELKVLE.MLPAVQYWYGIK....DALTE..QGST...
[Stachybotrys_chlorohalonata_IBT_40285]_KFA62815.          .....ASP.KYPVVLAHGLLGFSELKVLE.MLPAVQYWYGIK....DALTA..QGST...
[Purpureocillium_lilacinum]_XP_018174914.1                 .....ATP.RHPIVLAHGLLGFSELSISA.LLPPLQYWHGIR....QALTA..QGCPL..
[Clonostachys_solani]_CAH0059244.1                         .....ATP.TYPIVLAHGLLGFSEIKLAQ.ALPSLQYWHGIQ....DALEQ..NGAT...
[Clonostachys_rosea]_VUC36940.1                            .....ATP.TYPIVLAHGLLGFSEIKLAQ.ALPSLQYWHGIQ....DALEQ..NGAT...
[Clonostachys_rhizophaga]_CAH0026503.1                     LTTNPATP.TYPIVLAHGLLGFSEIKFAQ.ALPSLQYWHGIQ....DALEQ..AGAT...
[Emericellopsis_atlantica]_XP_046114137.1                  .....QTP.KYPIVLAHGLLGFSELQLTP.LLPSVQYWHGIR....DALAV..QGAR...
[Acremonium_chrysogenum_ATCC_11550]_KFH46845.1             .....ATP.RYPIVLAHGLLGFSELQLTR.YLPAVQYWYGIK....DALAA..QGAT...
[Pochonia_chlamydosporia_170]_XP_018148143.1               .....ATP.KYPIVLAHGLLGFSELHVPP..FPRIQYWHGIR....EALTA..QGAT...
[Epichloe_festucae_Fl1]_QPG98063.1                         .....AAP.KYPIVLAHGLLGFSELRL.P.VLPTIHYWHGIK....EALTA..QGAR...
[Colletotrichum_incanum]_KZL82863.1                        .....ETP.KNPIVLAHGLMGFAELRLGA.YVPPIHYWRGIS....DSLSTLSGPSN...
[Colletotrichum_simmondsii]_KXH39849.1                     .....DTP.KNPIVLAHGLMGFSELRLGA.YVPPIHYWRGIS....DSLSTLSGPSN...
[Colletotrichum_sojae]_KAF6819546.1                        .....DTP.KNPIVLAHGLMGFDELRLGS.YVPPIHYWHGIA....DALGALVGDHS...
[Bacillus_licheniformis]_CAB95850.2_RN2                    .....AAE.HNPVVMVHGIGGAS............YNFASIK....SYLVG..QGWDRNQ
[Trichosporon_asahii_var._asahii]_AGN98126.1_TaLi          .....AQPGKEPIVFLHGLPAPPGLNFLT.KLP..............VFAS..KGYC...
```

```
2Z5G_1|Chains                                           β2                α2           β3      α3       β4
                                                         50        60        70        80        90
2Z5G_1|Chains                                           .TYTLAVGPLSSN..........WDRACEAYAQLVGGTVD..YGAAHAAKHGHARF..GR
[Trichoderma_harzianum]_ThaL                            .VITATVPPSGSI..........EERAAKLAADILAQTTA...ASLPSAQD.........
[Trichoderma_harzianum]_KKO99473.1                      .VITATVPPSGSI..........EERAAKLAADILAQTTA...ASLPSAQD.........
[Trichoderma_guizhouense]_OPB43634.1                    .VITATVPPSGSI..........EERAAKLAADILAQTTA...ASLPSAQD.........
[Trichoderma_simmonsii]_QYS94233.1                      .VITATVPPSGSI..........EERAAKLAADILAQTTA...ASLPDSQD.........
[Trichoderma_harzianum_CBS_226.95]_XP_024777038.1       .VITATVPPSGSI..........EERAAKLAADILAQTTA...ASLPSAQD.........
[Trichoderma_harzianum]_PKK42226.1                      .VITATVPPSGSI..........EERAAKLAADILAQTTA...ASLPSAQD.........
[Trichoderma_lentiforme]_KAF3077685.1_                  .VITATVPPSGSI..........EERAAKLAADILAQTTA...ASLPSAQD.........
[Trichoderma_harzianum]_PNP59423.1                      .VITATVPPSGSI..........EERAAKLAADILAQTAA...ASLPS............
[Trichoderma_virens_FT-333]_UKZ73321.1                  .VITASVPPSGSI..........EERAAKLAADILAQTTA...SSLSA............
[Trichoderma_virens_Gv29-8]_XP_013960763.1              .VITASVPPSGSI..........EERAAKLAADILAQTTA...SLSSA............
[Trichoderma_asperellum_CBS_433.97]_XP_024756506.       .VITASVPPTGSI..........EERAAKLAADILAQTST...H................
[Trichoderma_atroviride_IMI_206040]_XP_013943551.       .VITASVPPTGSI..........EERAAKLAADILAQTSR...D................
[Trichoderma_gamsii]_PNP38305.1                         .VITASVPPSGSI..........EERAAKLAADIVAQTSR....................
[Trichoderma_gamsii]_XP_018663207.1                     .VITASVPPSGSI..........EERAAKLAADIVAQTSR....................
[Trichoderma_arundinaceum]_RFU75476.1                   .VITASVPPSGSI..........EERAAKLAADILAQTAS...SLDTP............
[Trichoderma_gracile]_KAH0498535.1                      .VLTTSVPPSGSI..........EERAEKLAADILAQTSA...AAAPSLIDS......DH
[Trichoderma_reesei_QM6a]_XP_006968245.1                .VLTTSVPPSGSI..........EERAEKLAADILAQTSA...A...SLSHS......NN
[Trichoderma_citrinoviride]_XP_024753310.1              .VVTTSVPPSGSI..........EERAEKLAADILAQNQ.....................
[Trichoderma_longibrachiatum_ATCC_18648]_PTB78011       .VIATSVPPSGSI..........EERAEKLAADILAQTSL...S..............NR
[Hirsutella_minnesotensis_3608]_KJZ78693.1              .VITASVPPSGSI..........QERAAKLAQDIAAACPPCQASSSGP............
[Hirsutella_rhossiliensis]_XP_044725020.1               .VTTASVPPSGSI..........EERAAKLAADIAAAFPP...SSPAP............
[Ophiocordyceps_sinensis_CO18]_EQK99704.1               .VITASVPPSGSI..........EERAAKLAADMAAALSS......SP............
[Tolypocladium_capitatum]_PNY29418.1                    .VMTASVPPSDAI..........EARAAKLAADIAADTSA...SDPSP............
[Tolypocladium_ophioglossoides_CBS_100239]_KND888       .VITASVPPSDAI..........EARAAKLAADIAAASAF....DPSP............
[Tolypocladium_paradoxum]_POR38551.1                    .VITASVPPSDAI..........EARAAKLAADIAAASSS...GT...............
[Drechmeria_coniospora]_ODA83100.1                      .IITASVPPSHGI..........EERAAKLAADISASIAS...S................
[Drechmeria_coniospora]_XP_040657415.1                  .IITASVPPSHGI..........EERAAKLAADISASIAS...S................
[Ophiocordyceps_unilateralis]_PFH57814.1                .VMTTSVPPSGSI..........EQRAAKLAADIATA.......................
[Fusarium_sp._AF-6]_RSL54391.1                          .VLTPTVPPSSSI..........ADRAAALRSALEAHAPA....................
[Fusarium_kuroshium]_RMJ16314.1                         .VLTPTVPPSSSI..........ADRAAALRSALEAHAPA....................
[Fusarium_sp._AF-8]_RSL70798.1                          .VLTPTVPPSSSI..........ADRAAALRSALEAHAPA....................
[Fusarium_ambrosium]_RSM11997.1                         .VLTPTVPPSSSI..........ADRAAALRSALEAHAPA....................
[Fusarium_floridanum]_RSL80024.1                        .VLTPTVPPSSSI..........ADRAAALRSALEAHAPA....................
[Fusarium_vanettenii_77-13-4]_XP_003050716.1            .VLTPTVPPSSSI..........ADRAAALRSALEAHAPA....................
[Fusarium_solani]_XP_046134414.1                        .VLTPTVPPSSSI..........ADRAAALRSALEAHAPA....................
[Fusarium_albosuccineum]_KAF4468415.1                   .VLTPAVPPSSSI..........ADRAAALRTALESHAPA....................
[Fusarium_decemcellulare]_KAF5011412.1                  .VLTPSVPPSSSI..........ADRAAALRTALESHAPA....................
[Fusarium_sarcochroum]_KAF4964785.1                     .VITPTVPPSSSI..........ADRAAALRSALEAHAPA....................
[Fusarium_avenaceum]_KAH6968652.1                       .VLTPTVPPSSSI..........ADRAAALRSALEAQSPA....................
[Fusarium_avenaceum]_KIL96274.1                         .VLTPTVPPSSSI..........ADRAAALRSALEAQSPA....................
[Fusarium_avenaceum]_KAG5656265.1                       .VLTPTVPPSSSI..........ADRAAALRSALEAQSPA....................
[Fusarium_tricinctum]_KAH7262197.1                      .VLTPTVPPSSSI..........ADRAAALRSALEAQSPA....................
[Fusarium_heterosporum]_KAF5663380.1                    .VLTPTVPPSSSI..........ADRAAALRSALEAHTPA....................
[Fusarium_sp._MPI-SDFR-AT-0072]_KAH7164412.1            .VLTPTVPPSSSI..........ADRAAALRSALEAHAPM....................
[Fusarium_oxysporum_f._sp._rapae]_KAG7414252.1          .VLTPTVPPSSSI..........ADRAAALRSALEAHAPM....................
[Fusarium_mangiferae]_XP_041681833.1                    .VLTPTVPPSSSI..........ADRATALRSALEAHAPM....................
[Fusarium_proliferatum]_CVL11341.1                      .VLTPTVPPSSSI..........ADRATALRSALEAHAPM....................
[Fusarium_proliferatum_ET1]_XP_031083673.1              .VLTPTVPPSSSI..........ADRATALRSALEAHAPM....................
[Fusarium_fujikuroi]_SCN78580.1                         .VLTPTVPPSSSI..........ADRATALRSALEAHAPM....................
[Fusarium_fujikuroi_IMI_58289]_XP_023430709.1           .VLTPTVPPSSSI..........ADRATALRSALEAHAPM....................
[Fusarium_sp._NRRL_25303]_KAF5626228.1                  .VLTPTVPPSSSI..........ADRATALRSALEAHAPM....................
[Fusarium_xylarioides]_KAG5746670.1                     .VLTPTVPPSSSI..........ADRATALRSALEAHAPM....................
[Fusarium_xylarioides]_KAG5788219.1                     .VLTPTVPPSSSI..........ADRATALRSALEAHAPM....................
[Fusarium_pseudocircinatum]_KAF5575212.1                .VLTPTVPPSSSI..........ADRATALRSALEAHAPM....................
[Fusarium_pseudoanthophilum]_KAF5575021.1               .VLTPTVPPSSSI..........ADRATALRSALEAHAPM....................
[Fusarium_verticillioides]_RBQ71412.1                   .VLTPSVPPSSSI..........ADRATALRSALEAHAPM....................
[Fusarium_verticillioides_7600]_XP_018746479.1          .VLTPSVPPSSSI..........ADRATALRSALEAHAPM....................
[Fusarium_oxysporum_NRRL_32931]_XP_031039788.1          .VLTPTVPPSSSI..........ADRATALRSALEALAPM....................
[Fusarium_oxysporum_f._sp._lycopersici_4287]_XP_0       .VLTPTVPPSSSI..........ADRATALRSALEALAPM....................
[Fusarium_oxysporum_f._sp._cepae]_RKK20771.1            .VLTPTVPPSSSI..........ADRATALRSALEALAPM....................
[Fusarium_redolens]_XP_046051341.1                      .VLTPTVPPSSSI..........ADRAAALRSALEAHAPM....................
[Fusarium_austroafricanum]_KAF4458007.1                 .VLTPTVPPSSSI..........ADRAAALRSALEAYAPM....................
[Fusarium_flagelliforme]_XP_045986929.1                 .VITPTVPPSSSI..........ADRAAALRSALEAHAPA....................
[Fusarium_sp._FIESC_RH6]_KAI1056250.1                   .VITPTVPPSSSI..........ADRAAALRSALEAHAPA....................
[Fusarium_sporotrichioides]_RGP72309.1                  .VITPTVPPSSSI..........ADRAAALRSALEAHAPA....................
[Fusarium_poae]_XP_044713592.1                          .VLTPAVPPSSSI..........ADRAAALRSALEAHAPV....................
[Fusarium_venenatum]_XP_025590637.1                     .VLTPAVPPSSSI..........ADRAAALRSALEAHAPV....................
[Fusarium_pseudograminearum]_KAF0641638.1               .VLTPAVPPSSSI..........ADRAAALRSALEAHAPA....................
[Fusarium_graminearum]_CAG1993854.1                     .VLTPAVPPSSSI..........ADRAAALRSALEAHAPA....................
[Fusarium_graminearum]_PCD31638.1                       .VLTPAVPPSSSI..........ADRAAALRSALEAHAPA....................
[Fusarium_graminearum]_CZS78629.1                       .VLTPAVPPSSSI..........ADRAAALRSALEAHAPA....................
[Fusarium_graminearum]_CAF3606094.1                     .VLTPAVPPSSSI..........ADRAAALRSALEAHAPA....................
[Fusarium_graminearum_PH-1]_XP_011318944.1              .VLTPAVPPSSSI..........ADRAAALRSALEAHAPA....................
[Fusarium_culmorum]_QPC68571.1                          .VLTPAVPPSSSI..........ADRAAALRSALEAHAPA....................
[Fusarium_pseudograminearum]_QPC77441.1                 .VLTPAVPPSSSI..........ADRAAALRSALEAHAPA....................
[Fusarium_longipes]_RGP79158.1                          .VLTPAVPPSSSI..........ADRAAALRSALEAHAPV....................
[Ilyonectria_destructans]_KAH7011941.1                  .VITPTVPPSSSI..........ADRAAALHAALAECAPA....................
[Ilyonectria_sp._MPI-CAGE-AT-0026]_KAH6985660.1         .VITPTVPPSSSI..........ADRAAALHAALAECAPA....................
[Ilyonectria_robusta]_XP_046104990.1                    .VITPTVPPSSSI..........ADRAAALHAALAECAPA....................
[Dactylonectria_estremocensis]_KAH7149692.1             .VITPSVPPSSSI..........ADRAAALHAALAACDPK....................
[Dactylonectria_macrodidyma]_KAH7148923.1               .VITPRVPPSSSI..........ADRAAALHEALAACEPK....................
[Thelonectria_olida]_KAH6899778.1                       .VLTPTVPPSGSI..........ADRAAALHDALAACSPM....................
[Stachybotrys_chartarum_IBT_7711]_KEY69997.1            .VITASVPPSSSI..........EERAAKLGADIAAQAPP....................
[Stachybotrys_chartarum_IBT_40293]_KFA50745.1           .VITASVPPSSSI..........EERAAKLGADIAAQAPP....................
[Stachybotrys_chlorohalonata_IBT_40285]_KFA62815.       .VITASVPPSSSI..........EERAAKLGADIASQAPP....................
[Purpureocillium_lilacinum]_XP_018174914.1              .VVAATVPPSSSI..........EERAAKLAVEISSALSS...SSSSSSVASSSPFPASR
[Clonostachys_solani]_CAH0059244.1                      .VITATVPPSSSI..........EQRAASLRDQIAAAR......................
[Clonostachys_rosea]_VUC36940.1                         .VITATVPPSSSI..........EQRAASLRDHITAAR......................
[Clonostachys_rhizophaga]_CAH0026503.1                  .VITATVPPSSSI..........EQRAASLRDQIAAAR......................
[Emericellopsis_atlantica]_XP_046114137.1               .VICASVPPSSSI..........EDRAKKLREDIDAAAST...TR...............
[Acremonium_chrysogenum_ATCC_11550]_KFH46845.1          .VLCASVPPSSSI..........EDRAAKLRDDIDASTRT...T................
[Pochonia_chlamydosporia_170]_XP_018148143.1            .VITATVPPSSSI..........AERAAKLADDITSAGVD...V................
[Epichloe_festucae_Fl1]_QPG98063.1                      .VITTSVPPSGSI..........GDRAAKLADEIAARH......................
[Colletotrichum_incanum]_KZL82863.1                     .IITTSVPPSGSI..........EERAAKLGADIAAKAG.....................
[Colletotrichum_simmondsii]_KXH39849.1                  .IITTSVPPSGSI..........EERAAKLGADIAAKAG.....................
[Colletotrichum_sojae]_KAF6819546.1                     .VITAAVPPSGSI..........EERAAKLGADIAAKAR.....................
[Bacillus_licheniformis]_CAB95850.2_RN2                 LFAIDFIDKTGNNR......NNGPRLSRFVKDVLDKTG......................
[Trichosporon_asahii_var._asahii]_AGN98126.1_TaLi       .VFAPTYGTTAGIIFAGAKVEDSSKEIAGVVDKVLASTG.....................
```

```
2Z5G_1|Chains                                                   η2          β5            α4               α5
                                                               100          110          120          130          140

2Z5G_1|Chains                                       TYPG......LLPELKR..GGRIHIIAHSQGGQTARMLVSLLENGSQEEREYAKAHNVSL
[Trichoderma_harzianum]_ThaL                        ............ADGHATTPPAVNIIAHSMGGLDARYMISLLRP...........SGI..
[Trichoderma_harzianum]_KKO99473.1                  ............ADGHATTPPAVNIIAHSMGGLDARYMISLLRP...........SGI..
[Trichoderma_guizhouense]_OPB43634.1                ............G......PPAVNIIAHSMGGLDARYMISLLRP...........SGI..
[Trichoderma_simmonsii]_QYS94233.1                  ............ADG....PPAVNIIAHSMGGLDARYMISLLRP...........SGI..
[Trichoderma_harzianum_CBS_226.95]_XP_024777038.1   ............AEGHA.SPPAVNIIAHSMGGLDARYMISLLRP...........SGI..
[Trichoderma_harzianum]_PKK42226.1                  ............AEGHA.SPPAVNIIAHSMGGLDARYMISLLRP...........SGI..
[Trichoderma_lentiforme]_KAF3077685.1_              ............ADDHATTPPAVNIIAHSMGGLDARYMISLLRP...........SGI..
[Trichoderma_harzianum]_PNP59423.1                  ............ADGHA..TPAVNIIAHSMGGLDARYMISLLRP...........SGI..
[Trichoderma_virens_FT-333]_UKZ73321.1              .............HDGK..PPAVNIIAHSMGGLDARYMISLLRP...........SGI..
[Trichoderma_virens_Gv29-8]_XP_013960763.1          .............HDGQ..PPAVNIIAHSMGGLDARYMISLLRP...........SGI..
[Trichoderma_asperellum_CBS_433.97]_XP_024756506.   .............KEHA..APTVNIIAHSMGGLDARYMISRLKPA.........LANV..
[Trichoderma_atroviride_IMI_206040]_XP_013943551.   .............ERDA..PPTVNIIAHSMGGLDARYMISRLKPV.........LKNV..
[Trichoderma_gamsii]_PNP38305.1                     .............DKHE..TPTVNIIAHSMGGLDARYMISRLKPV.........LQNV..
[Trichoderma_gamsii]_XP_018663207.1                 .............DKHE..TPTVNIIAHSMGGLDARYMISRLKPV.........LQNV..
[Trichoderma_arundinaceum]_RFU75476.1               .............RNGQ..APTVNIIAHSMGGLDARYMISRLQP...........LNI..
[Trichoderma_gracile]_KAH0498535.1                  NNNNRALRHNGGQNGQE..TPVVNIIAHSMGGLDARYMISRLRP...........RGI..
[Trichoderma_reesei_QM6a]_XP_006968245.1            NNNNNNNKNTNDRDSRL..TPVVNIIAHSMGGLDARYMISRLRP...........RGI..
[Trichoderma_citrinoviride]_XP_024753310.1          ...................TPMVNIIAHSMGGLDARYMISRLQP...........AGI..
[Trichoderma_longibrachiatum_ATCC_18648]_PTB78011   DDKKLVRDGGRQGEANS..SPVVNIIAHSMGGLDARYMISRLRP...........RGI..
[Hirsutella_minnesotensis_3608]_KJZ78693.1          ............GAGNG..PVPVNIIAHSMGGLDARYMISHLQPE..........GKV..
[Hirsutella_rhossiliensis]_XP_044725020.1           ............RTGEG..TVPVNIIAHSMGGLDARYLISHLQPQ.........GRQV..
[Ophiocordyceps_sinensis_CO18]_EQK99704.1           ............RAAQG..PISVNIIAHSMGGLDARYLISHLQPK.........GGPV..
[Tolypocladium_capitatum]_PNY29418.1                ............SPGRP..PTPVNIIAHSMGGLDARYMISHLLRA.........TPNV..
[Tolypocladium_ophioglossoides_CBS_100239]_KND888   ............SPSR...PTPVNIIAHSMGGLDARYMISHLLRA.........TPNV..
[Tolypocladium_paradoxum]_POR38551.1                ...................PTPVNIIAHSMGGLDARYMVSHLLRA.........TPDV..
[Drechmeria_coniospora]_ODA83100.1                  ............KFSGD..PVPVNIIAHSMGGLDARYMISRLQRA.........NSNF..
[Drechmeria_coniospora]_XP_040657415.1              ............KFSGD..PVPVNIIAHSMGGLDARYMISRLQRA.........NSNF..
[Ophiocordyceps_unilateralis]_PFH57814.1            ...................RSPVNVIAHSMGGLDARYMISRLPPH..........RRA..
[Fusarium_sp._AF-6]_RSL54391.1                      ...................PAAVTIIAHSMGGLDARYMLAHLKP...........LPV..
[Fusarium_kuroshium]_RMJ16314.1                     ...................PEAVTIIAHSMGGLDARYMLAHLKP...........LPI..
[Fusarium_sp._AF-8]_RSL70798.1                      ...................PEAVTIIAHSMGGLDARYMLAHLKP...........LPV..
[Fusarium_ambrosium]_RSM11997.1                     ...................PEAVTIIAHSMGGLDARYMLAHLKP...........LPV..
[Fusarium_floridanum]_RSL80024.1                    ...................PEAVTIIAHSMGGLDARYMLAHLKP...........LPI..
[Fusarium_vanettenii_77-13-4]_XP_003050716.1        ...................PEAVTIIAHSMGGLDARYMLAHLKP...........LPV..
[Fusarium_solani]_XP_046134414.1                    ...................PEAVTIIAHSMGGLDARYMLAHLNP...........LPV..
[Fusarium_albosuccineum]_KAF4468415.1               ...................PEAVTIIAHSMGGLDARYMLAHLAP...........LPI..
[Fusarium_decemcellulare]_KAF5011412.1              ...................PEAVTIIAHSMGGLDARYMLAHLTP...........LPI..
[Fusarium_sarcochroum]_KAF4964785.1                 ...................PEAFTIVAHSMGGLDARYMLSHLAP...........LPV..
[Fusarium_avenaceum]_KAH6968652.1                   ...................PEAFTIVAHSMGGLDARYMLSHLEP...........LPI..
[Fusarium_avenaceum]_KIL96274.1                     ...................PEAFTIVAHSMGGLDARYMLSHLEP...........LPV..
[Fusarium_avenaceum]_KAG5656265.1                   ...................PEAFTIVAHSMGGLDARYMLSHLEP...........LPV..
[Fusarium_tricinctum]_KAH7262197.1                  ...................PEAFTIVAHSMGGLDARYMLSHLEP...........LPV..
[Fusarium_heterosporum]_KAF5663380.1                ...................LEAVTIVAHSMGGLDARYMLSHLEP...........LPV..
[Fusarium_sp._MPI-SDFR-AT-0072]_KAH7164412.1        ...................HEAVTIVAHSMGGLDARYMLSHLAP...........LPV..
[Fusarium_oxysporum_f._sp._rapae]_KAG7414252.1      ...................HEAVTIVAHSMGGLDARYMLSHLAP...........LPV..
[Fusarium_mangiferae]_XP_041681833.1                ...................PEAVTIVAHSMGGLDARYMLSHLAP...........LPV..
[Fusarium_proliferatum]_CVL11341.1                  ...................PEAVTIIAHSMGGLDARYMLSHLTP...........LPV..
[Fusarium_proliferatum_ET1]_XP_031083673.1          ...................PEAVTIVAHSMGGLDARYMLSHLTP...........LPV..
[Fusarium_fujikuroi]_SCN78580.1                     ...................PEAVTIVAHSMGGLDARYMLSHLAP...........LPV..
[Fusarium_fujikuroi_IMI_58289]_XP_023430709.1       ...................PEAVTIVAHSMGGLDARYMLSHLAP...........LPV..
[Fusarium_sp._NRRL_25303]_KAF5626228.1              ...................PEAVTIVAHSMGGLDARYMLSHLAP...........LPV..
[Fusarium_xylarioides]_KAG5746670.1                 ...................PEAVTIVAHSMGGLDARYMLSHLAP...........LPV..
[Fusarium_xylarioides]_KAG5788219.1                 ...................PEAVTIVAHSMGGLDARYMLSHLAL...........LPV..
[Fusarium_pseudocircinatum]_KAF5575212.1            ...................PEAVTIVAHSMGGLDARYMLSHLAP...........LPV..
[Fusarium_pseudoanthophilum]_KAF5575021.1           ...................PEAVTIVAHSMGGLDARYMLSHLAP...........LPV..
[Fusarium_verticillioides]_RBQ71412.1               ...................PEAVTIVAHSMGGLDARYMLSHLAP...........LPV..
[Fusarium_verticillioides_7600]_XP_018746479.1      ...................PEAVTIVAHSMGGLDARYMLSHLAP...........LPV..
[Fusarium_oxysporum_NRRL_32931]_XP_031039788.1      ...................PQAVTIVAHSMGGLDTRYMLSHLAP...........LPV..
[Fusarium_oxysporum_f._sp._lycopersici_4287]_XP_0   ...................PQAVTIVAHSMGGLDTRYMLSHLAP...........LPV..
[Fusarium_oxysporum_f._sp._cepae]_RKK20771.1        ...................PQAVTIVAHSMGGLDTRYMLSHLAP...........LPV..
[Fusarium_redolens]_XP_046051341.1                  ...................PEAVTIVAHSMGGLDARYMLSHLAP...........LPV..
[Fusarium_austroafricanum]_KAF4458007.1             ...................PEAVTIVAHSMGGLDARYMLSHLAP...........LPV..
[Fusarium_flagelliforme]_XP_045986929.1             ...................PEAFTIVAHSMGGLDARYMLSHLEP...........LPV..
[Fusarium_sp._FIESC_RH6]_KAI1056250.1               ...................PEAFTIVAHSMGGLDARYMLSHLEP...........LPV..
[Fusarium_sporotrichioides]_RGP72309.1              ...................PEAVTIVAHSMGGLDARYMLSRLEP...........LPV..
[Fusarium_poae]_XP_044713592.1                      ...................PEAVTIVAHSMGGLDARYMLSHLEP...........LPV..
[Fusarium_venenatum]_XP_025590637.1                 ...................PEAVTIVAHSMGGLDARYMLSHLEP...........LPV..
[Fusarium_pseudograminearum]_KAF0641638.1           ...................PEAVTIVAHSMGGLDARYMLSHLEP...........LPV..
[Fusarium_graminearum]_CAG1993854.1                 ...................PEAVTIVAHSMGGLDARYMLSHLEP...........LPV..
[Fusarium_graminearum]_PCD31638.1                   ...................PEAVTIVAHSMGGLDARYMLSHLEP...........LPV..
[Fusarium_graminearum]_CZS78629.1                   ...................PEAVTIVAHSMGGLDARYMLSHLEP...........LPV..
[Fusarium_graminearum]_CAF3606094.1                 ...................PEAVTIVAHSMGGLDARYMLSHLEP...........LPV..
[Fusarium_graminearum_PH-1]_XP_011318944.1          ...................PEAVTIVAHSMGGLDARYMLSHLEP...........LPV..
[Fusarium_culmorum]_QPC68571.1                      ...................PEAVTIVAHSMGGLDARYMLSHLDP...........LPV..
[Fusarium_pseudograminearum]_QPC77441.1             ...................PEAVTIVAHSMGGLDARYMLSHLEP...........LPV..
[Fusarium_longipes]_RGP79158.1                      ...................PEAVTIVAHSMGGLDARYMLSHLEP...........LPV..
[Ilyonectria_destructans]_KAH7011941.1              ...................PDAVNIIAHSMGGLDARYMLAHMSP...........PPV..
[Ilyonectria_sp._MPI-CAGE-AT-0026]_KAH6985660.1     ...................PDAVNIIAHSMGGLDARYMLAHMSP...........PPV..
[Ilyonectria_robusta]_XP_046104990.1                ...................PDAVNIIAHSMGGLDVRYMLAHMSP...........PPV..
[Dactylonectria_estremocensis]_KAH7149692.1         ...................PEAVNIIAHSMGGLDARYMLAHMSP...........APV..
[Dactylonectria_macrodidyma]_KAH7148923.1           ...................PEAVNIIAHSMGGLDARYMLAHISP...........API..
[Thelonectria_olida]_KAH6899778.1                   ...................PEAVNIVAHSMGGLDARYMLSHMAP...........TPV..
[Stachybotrys_chartarum_IBT_7711]_KEY69997.1        ...................NVPVNIIAHSMGGLDARYMVANLRP...........KGV..
[Stachybotrys_chartarum_IBT_40293]_KFA50745.1       ...................NVPVNIIAHSMGGLDARYMVANLRP...........KGV..
[Stachybotrys_chlorohalonata_IBT_40285]_KFA62815.   ...................NVPVNIIAHSMGGLDARYMVANLRP...........KGV..
[Purpureocillium_lilacinum]_XP_018174914.1          GSSG.AADVGARGDDDT..RTPVNIIAHSMGGLDARHLISSILPSS.......PQHNF..
[Clonostachys_solani]_CAH0059244.1                  ...................PSAVNIVAHSMGGLDARYLASRLAP...........DSV..
[Clonostachys_rosea]_VUC36940.1                     ...................PSAVNIVAHSMGGLDARYLASRLAP...........DSV..
[Clonostachys_rhizophaga]_CAH0026503.1              ...................PSAVNIVAHSMGGLDARYLASCLAP...........DSV..
[Emericellopsis_atlantica]_XP_046114137.1           ...................DGRVNIIAHSMGGLDARYMISHLPP...........KYV..
[Acremonium_chrysogenum_ATCC_11550]_KFH46845.1      .............DGSR..AEKVNIIAHSMGGLDARYMIRHLPP...........RTA..
[Pochonia_chlamydosporia_170]_XP_018148143.1        ...................SAGVNVVAHSMGGLDARWMISNILKP.........ERSL..
[Epichloe_festucae_Fl1]_QPG98063.1                  ...................SGPVNIIAHSMGGLDARYMISKMATQ.........TQRI..
[Colletotrichum_incanum]_KZL82863.1                 ...................GRAVNIVAHSMGGLDARYMISHLKP...........ANV..
[Colletotrichum_simmondsii]_KXH39849.1              ...................GRAVNIVAHSMGGLDARYMISHLKP...........RDV..
[Colletotrichum_sojae]_KAF6819546.1                 ...................GRSVNIIAHSMGGLDARYMISHLKP...........RDV..
[Bacillus_licheniformis]_CAB95850.2_RN2             ...................AKKVDIVAHSMGGANTLYYIKNLDGGD.............
[Trichosporon_asahii_var._asahii]_AGN98126.1_TaLi   ...................ASKVNLVAHSMGTLVAAYYLKFDGGGD.............
                                                                                *
```

```
2Z5G_1|Chains                                           η3       β6      TT  η4        α6
                                                          150      160      170      180      190       200
2Z5G_1|Chains                                           SPLFEGGHHFVLSVTTIATPHDGTTLVNMVDFTDRFFDL..QKA.VLEAAAVASNVPYTS
[Trichoderma_harzianum]_ThaL                            .........RIASLVTIATPHRGSS.....FADYLVER..GAG.PLH..........LP
[Trichoderma_harzianum]_KKO99473.1                      .........RIASLVTIATPHRGSS.....FADYLVER..GAG.PLH..........LP
[Trichoderma_guizhouense]_OPB43634.1                    .........RIASLVTIATPHRGSS.....FADYLVER..GAG.PLH..........LP
[Trichoderma_simmonsii]_QYS94233.1                      .........RIASLVTIATPHRGSS.....FADYLIER..GAG.PLH..........LP
[Trichoderma_harzianum_CBS_226.95]_XP_024777038.1       .........QIASLVTIATPHRGSS.....FADYLVER..GAG.PLH..........LP
[Trichoderma_harzianum]_PKK42226.1                      .........QIASLVTIATPHRGSS.....FADYLVER..GAG.PLH..........LP
[Trichoderma_lentiforme]_KAF3077685.1_                  .........RIASLVTIATPHRGSS.....FADYLVER..GAG.PLH..........LP
[Trichoderma_harzianum]_PNP59423.1                      .........RIASLVTIATPHRGSA.....FADYLVER..GAG.PLH..........LP
[Trichoderma_virens_FT-333]_UKZ73321.1                  .........RIASLVTIATPHRGSA.....FADYLVER..GAG.PLH..........LP
[Trichoderma_virens_Gv29-8]_XP_013960763.1              .........RIASLVTIATPHRGSA.....FADYLVER..GAG.PLH..........LP
[Trichoderma_asperellum_CBS_433.97]_XP_024756506.       .........RVASLVTIATPHRGSP.....FADYLVER..GAG.PIH..........LP
[Trichoderma_atroviride_IMI_206040]_XP_013943551.       .........RVASLVTIATPHRGSP.....FADYLVER..GAG.PLH..........LP
[Trichoderma_gamsii]_PNP38305.1                         .........RVASLVTIATPHRGSP.....FADYLVER..GAG.PIH..........LP
[Trichoderma_gamsii]_XP_018663207.1                     .........RVASLVTIATPHRGSP.....FADYLVER..GAG.PIH..........LP
[Trichoderma_arundinaceum]_RFU75476.1                   .........NIASLVTIATPHRGSP.....FADYLVER..GAG.PIH..........LP
[Trichoderma_gracile]_KAH0498535.1                      .........RVASLVTIATPHRGSP.....FADYLVDS..DAGSPLH..........LP
[Trichoderma_reesei_QM6a]_XP_006968245.1                .........KVASLVTIATPHRGSP.....FADYLVDS..DAGSPLH..........LP
[Trichoderma_citrinoviride]_XP_024753310.1              .........KVASLVTIATPHRGSP.....FADYLVES..DSS.PVH..........LP
[Trichoderma_longibrachiatum_ATCC_18648]_PTB78011       .........KVASLVTIATPHRGSP.....FADYLVES..DTS.PLH..........LP
[Hirsutella_minnesotensis_3608]_KJZ78693.1              .........AVASLTTIATPHRGSS.....FADYLLEK..GAG.PIY..........LP
[Hirsutella_rhossiliensis]_XP_044725020.1               .........TVASLTTIATPHRGSM.....FADYLFDK..GAG.PIY..........LP
[Ophiocordyceps_sinensis_CO18]_EQK99704.1               .........TVASLTTIATPHRGSM.....FADYLLDE..GAG.PFH..........LP
[Tolypocladium_capitatum]_PNY29418.1                    .........RIASLTTISTPHRGST.....FADYLLEE..GAG.PIY..........LP
[Tolypocladium_ophioglossoides_CBS_100239]_KND888       .........RVASLTTISTPHRGST.....FADYLLKE..GAG.PIY..........LP
[Tolypocladium_paradoxum]_POR38551.1                    .........RVASLTTISTPHRGSA.....FADYLLDE..GAG.PMH..........LP
[Drechmeria_coniospora]_ODA83100.1                      .........RVAALVTVGTPHRGSP.....FADYVLHE..SAG.PLY..........LP
[Drechmeria_coniospora]_XP_040657415.1                  .........RVAALVTVGTPHRGSP.....FADYVLHE..SAG.PLY..........LP
[Ophiocordyceps_unilateralis]_PFH57814.1                .........AVASLTTISTPHRGSA.....FADYLLAA..G.G.PLH..........LP
[Fusarium_sp._AF-6]_RSL54391.1                          .........RVAALVTVATPHRGSA.....FADYLLDE..QVS.PLH..........LP
[Fusarium_kuroshium]_RMJ16314.1                         .........RVAALVTVATPHRGSA.....FADYLLDE..QVS.PLH..........LP
[Fusarium_sp._AF-8]_RSL70798.1                          .........RVAALVTVATPHRGSA.....FADYLLDE..QVS.PLH..........LP
[Fusarium_ambrosium]_RSM11997.1                         .........RVAALVTVATPHRGSA.....FADYLLDE..QVS.PLH..........LP
[Fusarium_floridanum]_RSL80024.1                        .........RVAALVTVATPHRGSA.....FADYLLDE..QVS.PLH..........LP
[Fusarium_vanettenii_77-13-4]_XP_003050716.1            .........RVAALVTVATPHRGSA.....FADYLLDE..QVS.PLH..........LT
[Fusarium_solani]_XP_046134414.1                        .........RVAALVTVATPHRGSA.....FADYLLDE..QVS.PLH..........LP
[Fusarium_albosuccineum]_KAF4468415.1                   .........RVAALVTVATPHRGSA.....FADYLLDE..QVS.PLH..........LP
[Fusarium_decemcellulare]_KAF5011412.1                  .........RVAALVTVATPHRGSA.....FADYLLDE..QVS.PLH..........LP
[Fusarium_sarcochroum]_KAF4964785.1                     .........RVAALVTVATPHRGSA.....FADYLLDE..EVS.PLH........RYLP
[Fusarium_avenaceum]_KAH6968652.1                       .........RVAALVTVATPHRGSA.....FADYLLDE..EVS.PLH........QYLP
[Fusarium_avenaceum]_KIL96274.1                         .........RVAALVTVATPHRGSA.....FADYLLDE..EVS.PLH........QYLP
[Fusarium_avenaceum]_KAG5656265.1                       .........RVAALVTVATPHRGSA.....FADYLLDE..EVS.PLH........QYLP
[Fusarium_tricinctum]_KAH7262197.1                      .........RVAALVTVATPHRGSA.....FADYLLDE..EVS.PLH........HYLP
[Fusarium_heterosporum]_KAF5663380.1                    .........RVAALVTVATPHRGSA.....FADYLLDE..EVS.PLH........HYLP
[Fusarium_sp._MPI-SDFR-AT-0072]_KAH7164412.1            .........RVAAMVTVATPHRGSA.....FADYLLDE..EVS.PLH........HYLP
[Fusarium_oxysporum_f._sp._rapae]_KAG7414252.1          .........RVAAMVTVATPHRGSA.....FADYLLDE..EVS.PLH........HYLP
[Fusarium_mangiferae]_XP_041681833.1                    .........RVAAVVTVATPHRGSA.....FADYLLDE..EVS.PLH........HYLP
[Fusarium_proliferatum]_CVL11341.1                      .........RVAAVVTVATPHRGSA.....FADYLLDE..EVS.PLH........HYLP
[Fusarium_proliferatum_ET1]_XP_031083673.1              .........RVAAVVTVATPHRGSA.....FADYLLDE..EVS.PLH........HYLP
[Fusarium_fujikuroi]_SCN78580.1                         .........RVAAVVTVATPHRGSA.....FADYLLDE..EVS.PLH........HYLP
[Fusarium_fujikuroi_IMI_58289]_XP_023430709.1           .........RVAAVVTVATPHRGSA.....FADYLLDE..EVS.PLH........HYLP
[Fusarium_sp._NRRL_25303]_KAF5626228.1                  .........RVAAVVTVATPHRGSA.....FADYLLDE..EVS.PLH........HYLP
[Fusarium_xylarioides]_KAG5746670.1                     .........RVAAVVTVATPHRGSA.....FADYLLDE..EVS.PLH........HYLP
[Fusarium_xylarioides]_KAG5788219.1                     .........RVAAVVTVATPHRGSA.....FADYLLDE..EVS.PLH........HYLP
[Fusarium_pseudocircinatum]_KAF5575212.1                .........RVAAVVTVATPHRGSA.....FADYLLDE..EVS.PLH........HYLP
[Fusarium_pseudoanthophilum]_KAF5575021.1               .........RVAAVVTVATPHRGSA.....FADYLLDE..EVS.PLH........HYLP
[Fusarium_verticillioides]_RBQ71412.1                   .........RVAAVVTVATPHRGSA.....FADYLLDE..EVS.PLH........HYLP
[Fusarium_verticillioides_7600]_XP_018746479.1          .........RVAAVVTVATPHRGSA.....FADYLLDE..EVS.PLH........HYLP
[Fusarium_oxysporum_NRRL_32931]_XP_031039788.1          .........RVAAVVTVATPHRGSA.....FADYLLDE..EVS.PLH........HYLP
[Fusarium_oxysporum_f._sp._lycopersici_4287]_XP_0       .........RVAAVVTVATPHRGSA.....FADYLLDE..EVS.PLH........HYLP
[Fusarium_oxysporum_f._sp._cepae]_RKK20771.1            .........RVAAVVTVATPHRGSA.....FADYLLDE..EVS.PLH........HYLP
[Fusarium_redolens]_XP_046051341.1                      .........RVAALVTVATPHRGSA.....FADYLLDE..EVS.PLH........HYLP
[Fusarium_austroafricanum]_KAF4458007.1                 .........HVAALVTVATPHRGSA.....FADYLLDE..EVS.PLH........QYLP
[Fusarium_flagelliforme]_XP_045986929.1                 .........RVAALVTVATPHRGSA.....FADYLLDE..EVS.PLH........RYLP
[Fusarium_sp._FIESC_RH6]_KAI1056250.1                   .........RVAALVTVATPHRGSA.....FADYLLDE..EVS.PLH........QYLP
[Fusarium_sporotrichioides]_RGP72309.1                  .........RVAALVTVATPHRGSA.....FADYLLDE..EVS.PVH........HYLP
[Fusarium_poae]_XP_044713592.1                          .........RVAALVTVATPHRGSA.....FADYLLDE..EVS.PVH........HYLP
[Fusarium_venenatum]_XP_025590637.1                     .........RVAALVTVATPHRGSA.....FADYLLDE..EVS.PVH........HYLP
[Fusarium_pseudograminearum]_KAF0641638.1               .........RVAALVTVATPHRGSA.....FADYLLDE..EVS.PVH........HYLP
[Fusarium_graminearum]_CAG1993854.1                     .........RVAALVTVATPHRGSA.....FADYLLDE..EVS.PVH........HYLP
[Fusarium_graminearum]_PCD31638.1                       .........RVAALVTVATPHRGSA.....FADYLLDE..EVS.PVH........HYLP
[Fusarium_graminearum]_CZS78629.1                       .........RVAALVTVATPHRGSA.....FADYLLDE..EVS.PVH........HYLP
[Fusarium_graminearum]_CAF3606094.1                     .........RVAALVTVATPHRGSA.....FADYLLDE..EVS.PVH........HYLP
[Fusarium_graminearum_PH-1]_XP_011318944.1              .........RVAALVTVATPHRGSA.....FADYLLDE..EVS.PVH........HYLP
[Fusarium_culmorum]_QPC68571.1                          .........RVAALVTVATPHRGSA.....FADYLLDE..EVS.PVH........HYLP
[Fusarium_pseudograminearum]_QPC77441.1                 .........RVAALVTVATPHRGSA.....FADYLLDE..EVS.PVH........HYLP
[Fusarium_longipes]_RGP79158.1                          .........RVAALVTVATPHRGSA.....FADYLLDE..EVS.PLH........HYLP
[Ilyonectria_destructans]_KAH7011941.1                  .........RVASLVTVATPHRGSA.....FADYLLEEG.GKA.PIH..........LP
[Ilyonectria_sp._MPI-CAGE-AT-0026]_KAH6985660.1         .........RVASLVTVATPHRGSA.....FADYLLEEG.GEA.PIH..........LP
[Ilyonectria_robusta]_XP_046104990.1                    .........RVASLVTVATPHRGSA.....FADYLLEEG.GEA.PIH..........LP
[Dactylonectria_estremocensis]_KAH7149692.1             .........RVASLVTVATPHRGSA.....FADYLLEEG.GEA.PIH..........LP
[Dactylonectria_macrodidyma]_KAH7148923.1               .........PVASLVTIATPHRGSA.....FADYLLEEG.GEA.PIH..........LP
[Thelonectria_olida]_KAH6899778.1                       .........KVASLVTVATPHRGSA.....FADYLLDK..NLA.PIH..........LP
[Stachybotrys_chartarum_IBT_7711]_KEY69997.1            .........RVASLVTIATPHRGSA.....FADFLLDE..QTA.PIH..........LP
[Stachybotrys_chartarum_IBT_40293]_KFA50745.1           .........RVASLVTIATPHRGSA.....FADFLLDE..QTA.PIH..........LP
[Stachybotrys_chlorohalonata_IBT_40285]_KFA62815.       .........RVASLVTVATPHRGSA.....FADFLLDE..QTA.PIY..........LP
[Purpureocillium_lilacinum]_XP_018174914.1              .........RVASLVTISTPHRGSP.....FADYVLSDSRRRS.LLH..........LP
[Clonostachys_solani]_CAH0059244.1                      .........AIKSITTIATPHRGSH.....FADFLMSD..S.A.PIH..........LP
[Clonostachys_rosea]_VUC36940.1                         .........VIKSITTIATPHRGSH.....FADFLMSD..S.A.PIH..........LP
[Clonostachys_rhizophaga]_CAH0026503.1                  .........AIKSITTIATPHRGSH.....FADFLMSD..S.A.PIH..........LP
[Emericellopsis_atlantica]_XP_046114137.1               .........DIASLVTVASPHRGSS.....FADVLLQED.GQA.LVH..........LP
[Acremonium_chrysogenum_ATCC_11550]_KFH46845.1          .........EVASLVTVSTPHRGSH.....FADAVLDEDAADG.PLY..........LP
[Pochonia_chlamydosporia_170]_XP_018148143.1            .........KVASLTTISTPHHGSP.....FADYVLHS..SSG.IMY..........LP
[Epichloe_festucae_Fl1]_QPG98063.1                      .........QVSSLTTVSTPHRGSP.....FADYVLHS..SSG.IMY..........LP
[Colletotrichum_incanum]_KZL82863.1                     .........KVMSLVTVATPHRGSA.....FADFLLDE..R.G.PIK..........LA
[Colletotrichum_simmondsii]_KXH39849.1                  .........KVLSLVTVATPHRGSA.....FADYLLDG..Q.G.PIK..........LA
[Colletotrichum_sojae]_KAF6819546.1                     .........DVRSLVTVATPHRGSA.....FADYLVDG..R.G.PIK..........LA
[Bacillus_licheniformis]_CAB95850.2_RN2                 .........KIENVIPIG...........GANGLVSS..RA...............LP
[Trichosporon_asahii_var._asahii]_AGN98126.1_TaLi       .........KVKTYVGFGPVYNGTT.....EASLRLLK..QW...............IP
```

```
                                                          η5         α7        α8       α9       α10
2Z5G_1|Chains                                             222   TT  2222222222  2222    22222  22222222222      TT
                                                          210       220        230      240       250           260
2Z5G_1|Chains                                           QVYDFKLDQWGLRRQPGESFDHYFERLKRSPVWTSTDTARYDLSVSGAEKLNQWVQASPN
[Trichoderma_harzianum]_ThaL                            RLYGV.IRRAGLGTSA.......FGQL..........TTRY.....MREEFNPRVRDDEA
[Trichoderma_harzianum]_KKO99473.1                      RLYGV.IRRAGLGTSA.......FGQL..........TTRY.....MREEFNPRVRDDEA
[Trichoderma_guizhouense]_OPB43634.1                    RLYGV.IRRAGLGTSA.......FGQL..........TTRY.....MREEFNPRVRDDEG
[Trichoderma_simmonsii]_QYS94233.1                      RLYGV.IRRAGLGTSA.......FGQL..........TTRY.....MREEFNPRVRDDEG
[Trichoderma_harzianum_CBS_226.95]_XP_024777038.1       RLYGV.IRRAGLGTSA.......FGQL..........TTRY.....MREEFNPRVRDDEG
[Trichoderma_harzianum]_PKK42226.1                      RLYGV.IRRAGLGTSA.......FGQL..........TTRY.....MREEFNPRVRDDEG
[Trichoderma_lentiforme]_KAF3077685.1_                  RLYGV.IRRAGLGTSA.......FGQL..........TTRY.....MREEFNPRVRDEEG
[Trichoderma_harzianum]_PNP59423.1                      RLYGV.IRRAGLGTSA.......FGQL..........TTRY.....MRDEFNPRVRDDEA
[Trichoderma_virens_FT-333]_UKZ73321.1                  RLYGV.IRRAGLGTSA.......FGQL..........TTRY.....MRDEFNPRVRDDEA
[Trichoderma_virens_Gv29-8]_XP_013960763.1              RLYGV.IRRAGLGTSA.......FGQL..........TTRY.....MRDEFNPRVRDDEA
[Trichoderma_asperellum_CBS_433.97]_XP_024756506.       RLYGI.IRRAGLGTSA.......FGQL..........TTRY.....MREEFNPRVRDDES
[Trichoderma_atroviride_IMI_206040]_XP_013943551.       RLYSI.IRRAGLGTSA.......FGQL..........TTRY.....MRDEFNPRVRDDEA
[Trichoderma_gamsii]_PNP38305.1                         RLYSI.IRRAGLGTSA.......FGQL..........TTRY.....MREEFNTKVLDDEA
[Trichoderma_gamsii]_XP_018663207.1                     RLYSI.IRRAGLGTSA.......FGQL..........TTRY.....MREEFNTKVLDDEA
[Trichoderma_arundinaceum]_RFU75476.1                   RLYGI.IRRAGLGTSA.......FGQL..........TTRY.....MREEFNPRVQDDES
[Trichoderma_gracile]_KAH0498535.1                      RLYSV.IRRAGLGTSA.......FGQL..........TTRY.....MRDEFNPRVPDDER
[Trichoderma_reesei_QM6a]_XP_006968245.1                RLYSV.IRRAGLGTSA.......FGQL..........TTRY.....MRHEFNPRVPDDQA
[Trichoderma_citrinoviride]_XP_024753310.1              RLYSV.IRRAGLGTSA.......FGQL..........TTRY.....MRDEFNPRVPDDPA
[Trichoderma_longibrachiatum_ATCC_18648]_PTB78011       RLYSV.IRRAGLGTSA.......FGQL..........TTRY.....MRDEFNPRVPDVEG
[Hirsutella_minnesotensis_3608]_KJZ78693.1              KLYGV.LKKAGLGTQA.......FAQL..........TTHY.....MVNEFNPQNPDNPS
[Hirsutella_rhossiliensis]_XP_044725020.1               KLYRL.LEKAGLGTKA.......FAQL..........TTRY.....MAQEFNPQTPDDPL
[Ophiocordyceps_sinensis_CO18]_EQK99704.1               RLYRV.LEKAGLGTKA.......FAQL..........TTRY.....MAQEFNPQTPDDPS
[Tolypocladium_capitatum]_PNY29418.1                    RLYGV.LQRAGLGTKA.......FAQL..........TTRY.....LADEFNPRTEDDPS
[Tolypocladium_ophioglossoides_CBS_100239]_KND888       KLYGV.LQRAGLGTKA.......FAQL..........TTRY.....MADEFNPRTVDDPS
[Tolypocladium_paradoxum]_POR38551.1                    RLYGV.LQRAGLGTRA.......FAQL..........TTRY.....MADEFNPRTEDDPE
[Drechmeria_coniospora]_ODA83100.1                      RLYSL.LERAGLGTRA.......FAQL..........TTRY.....LATEFNPRTRDDPA
[Drechmeria_coniospora]_XP_040657415.1                  RLYSL.LERAGLGTRA.......FAQL..........TTRY.....LATEFNPRTRDDPA
[Ophiocordyceps_unilateralis]_PFH57814.1                RLYGL.LRRVGLGTQA.......FEQL..........TTLY.....MTDDFNPRTEDDPS
[Fusarium_sp._AF-6]_RSL54391.1                          RLYWM.LERAGLGTAA.......FAQL..........TRRY.....MADEFNPSTPDCSD
[Fusarium_kuroshium]_RMJ16314.1                         RLYWM.LERAGLGTAA.......FAQL..........TRRY.....MADEFNPSTPDCPD
[Fusarium_sp._AF-8]_RSL70798.1                          RLYWM.LERAGLGTAA.......FAQL..........TRRY.....MADEFNPSTPDCPD
[Fusarium_ambrosium]_RSM11997.1                         RLYWM.LERAGLGTAA.......FAQL..........TRRY.....MADEFNPSTPDCPD
[Fusarium_floridanum]_RSL80024.1                        RLYWM.LERAGLGTAA.......FAQL..........TRRY.....MADEFNPSTPDCPD
[Fusarium_vanettenii_77-13-4]_XP_003050716.1            RLYWM.LERAGLGTAA.......FAQL..........TRRY.....MADEFNPSTPDNPD
[Fusarium_solani]_XP_046134414.1                        RLYWM.LERAGLGTAA.......FAQL..........TRRY.....MTDEFNPSTPDCPD
[Fusarium_albosuccineum]_KAF4468415.1                   RLYKT.LERAGLGTAA.......FAQL..........TRRY.....MADEFNPSTPDCAD
[Fusarium_decemcellulare]_KAF5011412.1                  RLYKT.LERAGLGTAA.......FAQL..........TRRY.....MADEFNPSTPDCAD
[Fusarium_sarcochroum]_KAF4964785.1                     RLYHT.LEKAGLGTAA.......FSQL..........TRRY.....MAEEFNPSTPDKPD
[Fusarium_avenaceum]_KAH6968652.1                       RLYQT.FEKAGLGTAA.......FSQL..........TRRY.....MAEEFNPSTPDRPD
[Fusarium_avenaceum]_KIL96274.1                         RLYQT.FEKAGLGTAA.......FSQL..........TRRY.....MAEEFNPSTPDRPD
[Fusarium_avenaceum]_KAG5656265.1                       RLYQT.FEKAGLGTAA.......FSQL..........TRRY.....MAEEFNPSTPDRPD
[Fusarium_tricinctum]_KAH7262197.1                      RLYQT.FEKAGLGTAA.......FSQL..........TRRY.....MAEEFNPSTPDRPD
[Fusarium_heterosporum]_KAF5663380.1                    RLYHT.LEKAGLGTAA.......FSQL..........TRRY.....MAEEFNPSTPDQPD
[Fusarium_sp._MPI-SDFR-AT-0072]_KAH7164412.1            QLYQT.FEKAGLGTAA.......FSQL..........TRRY.....MAKEFNPSTPDQPG
[Fusarium_oxysporum_f._sp._rapae]_KAG7414252.1          QLYQT.FEKAGLGTAA.......FSQL..........TRRY.....MAKEFNPSTPDQPD
[Fusarium_mangiferae]_XP_041681833.1                    QLYQT.FEKAGLGTAA.......FSQL..........TRRY.....MAEEFNPSTPDQPD
[Fusarium_proliferatum]_CVL11341.1                      QLYQT.FEKAGLGTAA.......FSQL..........TRRY.....MAEEFNPSTPDQPD
[Fusarium_proliferatum_ET1]_XP_031083673.1              QLYQT.FEKAGLGTAA.......FSQL..........TRRY.....MAEEFNPSTPDQPD
[Fusarium_fujikuroi]_SCN78580.1                         QLYQT.FEKAGLGTAA.......FSQL..........TRRY.....MAEEFNPSTPDQPD
[Fusarium_fujikuroi_IMI_58289]_XP_023430709.1           QLYQT.FEKAGLGTAA.......FSQL..........TRRY.....MAEEFNPSTPDQPD
[Fusarium_sp._NRRL_25303]_KAF5626228.1                  QLYQT.LEKAGLGTAA.......FSQL..........TRRY.....MAEEFNPSTPDQPD
[Fusarium_xylarioides]_KAG5746670.1                     QLYQT.FEKAGLGTAA.......FSQL..........TRRY.....MAEEFNPSTPDQPD
[Fusarium_xylarioides]_KAG5788219.1                     QLYQT.FEKAGLGTAA.......FSQL..........TRRY.....MAEEFNPSTPDQPD
[Fusarium_pseudocircinatum]_KAF5575212.1                QLYQT.FEKAGLGTAA.......FSQL..........TRRY.....MAEEFNPSTPDQPD
[Fusarium_pseudoanthophilum]_KAF5575021.1               QLYQT.FEKAGLGTAA.......FSQL..........TRRY.....MAEEFNPSTPDQPD
[Fusarium_verticillioides]_RBQ71412.1                   QLYQT.FEKAGLGTAA.......FSQL..........TRRY.....MAEEFNPSTPDQPD
[Fusarium_verticillioides_7600]_XP_018746479.1          QLYQT.FEKAGLGTAA.......FSQL..........TRRY.....MAEEFNPSTPDQPD
[Fusarium_oxysporum_NRRL_32931]_XP_031039788.1          QLYQT.FEKAGLGTAA.......FSQL..........TRRY.....MAEEFNPSTPDQPD
[Fusarium_oxysporum_f._sp._lycopersici_4287]_XP_0       QLYQT.FEKAGLGTAA.......FSQL..........TRRY.....MAEEFNPSTPDQPD
[Fusarium_oxysporum_f._sp._cepae]_RKK20771.1            QLYQT.FEKAGLGTAA.......FSQL..........TRRY.....MAEEFNPSTPDQPD
[Fusarium_redolens]_XP_046051341.1                      RLYQT.FEKAGLGTAA.......FSQL..........TRRY.....MAEEFNPSTPDQPD
[Fusarium_austroafricanum]_KAF4458007.1                 QLYQT.LEKAGLGTAA.......FSQL..........TRRY.....MAEEFNPSTPDQPD
[Fusarium_flagelliforme]_XP_045986929.1                 RLYQT.FERAGLGTAA.......FSQL..........TRRY.....MAEEFNPSTPDRAD
[Fusarium_sp._FIESC_RH6]_KAI1056250.1                   RLYQT.FERAGLGTAA.......FSQL..........TRRY.....MAEEFNPSTPDRAD
[Fusarium_sporotrichioides]_RGP72309.1                  QLYKT.LERAGLGTAA.......FSQL..........TRRY.....MAEEFNPSTPDQPD
[Fusarium_poae]_XP_044713592.1                          QLYKT.LERAGLGTAA.......FSQL..........TRRY.....MAEEFNPSTPDQPD
[Fusarium_venenatum]_XP_025590637.1                     QLYKT.LERAGLGTAA.......FSQL..........TRRY.....MAEEFNPSTPDQPD
[Fusarium_pseudograminearum]_KAF0641638.1               QLYKT.LERAGLGTAA.......FSQL..........TRRY.....MAEEFNPSTPDQPD
[Fusarium_graminearum]_CAG1993854.1                     QLYKT.LERAGLGTAA.......FSQL..........TRRY.....MAEEFNPSTPDQPD
[Fusarium_graminearum]_PCD31638.1                       QLYKT.LERAGLGTAA.......FSQL..........TRRY.....MAEEFNPSTPDQPD
[Fusarium_graminearum]_CZS78629.1                       QLYKT.LERAGLGTAA.......FSQL..........TRRY.....MAEEFNPSTPDQPD
[Fusarium_graminearum]_CAF3606094.1                     QLYKT.LERAGLGTAA.......FSQL..........TRRY.....MAEEFNPSTPDQPD
[Fusarium_graminearum_PH-1]_XP_011318944.1              QLYKT.LERAGLGTAA.......FSQL..........TRRY.....MAEEFNPSTPDQPD
[Fusarium_culmorum]_QPC68571.1                          QLYKT.LERAGLGTAA.......FSQL..........TRRY.....MAEEFNPSTPDQPD
[Fusarium_pseudograminearum]_QPC77441.1                 QLYKT.LERAGLGTAA.......FSQL..........TRRY.....MAEEFNPSTPDQPD
[Fusarium_longipes]_RGP79158.1                          QLYKT.LERAGLGTAA.......FSQL..........TRRY.....MAEEFNPNTPDQPD
[Ilyonectria_destructans]_KAH7011941.1                  RLYGA.IERAGFGTAA.......FAQL..........TRRY.....MQSEFNPATPDSPD
[Ilyonectria_sp._MPI-CAGE-AT-0026]_KAH6985660.1         RLYGA.IERAGFGTAA.......FAQL..........TRRY.....MQSEFNPATPDSPD
[Ilyonectria_robusta]_XP_046104990.1                    RLYGA.IERAGFGTAA.......FAQL..........TRRY.....MQSEFNPATPDSPD
[Dactylonectria_estremocensis]_KAH7149692.1             RLYGA.IERAGFGTAA.......FAQL..........TRRY.....MQDEFNPATRDSPD
[Dactylonectria_macrodidyma]_KAH7148923.1               RLYGA.IERAGFGTAA.......FAQL..........TQRY.....MQDEFNPATQDSPD
[Thelonectria_olida]_KAH6899778.1                       RLYGA.IERAGFGTAA.......FAQL..........TRRY.....MTDEFNAATPDDPD
[Stachybotrys_chartarum_IBT_7711]_KEY69997.1            KLYWA.LERTGLGTRA.......FAQL..........TRRY.....LTEEFNSATKDIPG
[Stachybotrys_chartarum_IBT_40293]_KFA50745.1           KLYWA.LERTGLGTRA.......FAQL..........TRRY.....LTEEFNSATKDIPG
[Stachybotrys_chlorohalonata_IBT_40285]_KFA62815.       KLYWA.LERTGLGTQA.......FAQL..........TKRY.....LTEEFNSATKDVPG
[Purpureocillium_lilacinum]_XP_018174914.1              RFYSL.FRAAGLSTRA.......FAQL..........GTRY.....LADDFNPANPDDPA
[Clonostachys_solani]_CAH0059244.1                      RLYGL.IERAGLGTQA.......FSQL..........TREY.....MTGTFNATVRDRDD
[Clonostachys_rosea]_VUC36940.1                         RLYGL.IERAGFGTQA.......FSQL..........TREY.....MTQTFNATVQDRDD
[Clonostachys_rhizophaga]_CAH0026503.1                  RLYGL.IERAGFGTQA.......FSQL..........TREY.....MTQTFNATVRDRDD
[Emericellopsis_atlantica]_XP_046114137.1               KLYGL.IERFGLGTAA.......FAQL..........TTSH.....MTKVFNPATPDSPS
[Acremonium_chrysogenum_ATCC_11550]_KFH46845.1          RLYGL.LERAGLDTAA.......FAQL..........TRAY.....MTGTFNPATPDRDS
[Pochonia_chlamydosporia_170]_XP_018148143.1            RLYRL.LERAGLSTSA.......FSQL..........TTSY.....MRDEFNPRTRDDEA
[Epichloe_festucae_Fl1]_QPG98063.1                      RLYGV.LERAGLGTKA.......FAQL..........TTGY.....MTGTFNRDVVDDDK
[Colletotrichum_incanum]_KZL82863.1                     NLYGL.IERAGLGTQA.......FGQL..........TQKY.....MEEEFNPRTPDNDE
[Colletotrichum_simmondsii]_KXH39849.1                  NLYGL.IERAGLGTQA.......FEQL..........TQRY.....MEEKFNPATPDSDE
[Colletotrichum_sojae]_KAF6819546.1                     NLYGL.IERAGLGTRA.......FGQL..........TRDY.....MEREFNPATPDSER
[Bacillus_licheniformis]_CAB95850.2_RN2                 ............GTDP.......NQKI....LY....TSVYS....................
[Trichosporon_asahii_var._asahii]_AGN98126.1_TaLi       GL.....................................TDAF.....CPGCIDAVPPSDVI
```

```
                                                      β7      β8       β9    TT  α11             α12            η6
2Z5G_1|Chains                                                                                               
                                                             270       280       290                   300

2Z5G_1|Chains                                          TYYLSFSTERTYRGALTGNHYPELGMNAFSA.VVCAPF..........LGSYRNPTLGID
[Trichoderma_harzianum]_ThaL                           VRYFSYG......................AA.IDEPSL..........LGAFRLPHGVVD
[Trichoderma_harzianum]_KKO99473.1                     VRYFSYG......................AA.IDEPSL..........LGAFRLPHGVVD
[Trichoderma_guizhouense]_OPB43634.1                   VRYFSYG......................AA.IDEPSL..........LGAFRLPHGVVD
[Trichoderma_simmonsii]_QYS94233.1                     VRYFSYG......................AA.IDEPSL..........LGAFRLPHGVVD
[Trichoderma_harzianum_CBS_226.95]_XP_024777038.1      VRYFSYG......................AA.IDEPSL..........LGAFRLPHGVVD
[Trichoderma_harzianum]_PKK42226.1                     VRYFSYG......................AA.IDEPSL..........LGAFRLPHGVVD
[Trichoderma_lentiforme]_KAF3077685.1_                 VRYFSYG......................AA.IDEPSL..........LGAFRLPHGVVD
[Trichoderma_harzianum]_PNP59423.1                     VRYFSYG......................AA.IDEPSL..........LGAFRLPHGVVD
[Trichoderma_virens_FT-333]_UKZ73321.1                 VRYFSYG......................AA.IDEPSL..........LGAFRLPHNVVD
[Trichoderma_virens_Gv29-8]_XP_013960763.1             VRYFSYG......................AA.IDEPSL..........LGAFRLPHNVVD
[Trichoderma_asperellum_CBS_433.97]_XP_024756506.      VRYFSYG......................AA.IDEPSL..........LGAFRLPYGVVG
[Trichoderma_atroviride_IMI_206040]_XP_013943551.      VRYFSYG......................AA.IDEPSL..........LGAFRLPHGVVG
[Trichoderma_gamsii]_PNP38305.1                        VRYFSYG......................AA.IDEPSL..........LGAFRLPYGVVG
[Trichoderma_gamsii]_XP_018663207.1                    VRYFSYG......................AA.IDEPSL..........LGAFRLPYGVVG
[Trichoderma_arundinaceum]_RFU75476.1                  VRYFSYG......................AA.IDEPSL..........LGAFRLPHGVVS
[Trichoderma_gracile]_KAH0498535.1                     VRYFSYG......................AM.IDEPSL..........LGAFRVPYGVIW
[Trichoderma_reesei_QM6a]_XP_006968245.1               VRYFSYG......................AM.IDEPSL..........LGAFRVPYGVIW
[Trichoderma_citrinoviride]_XP_024753310.1             VRYFSYG......................AM.IDEPSL..........LGAFRVPYGVVK
[Trichoderma_longibrachiatum_ATCC_18648]_PTB78011      VRYFSYG......................AM.IDEPSL..........LGAFRVPHGVIW
[Hirsutella_minnesotensis_3608]_KJZ78693.1             VRYFSYG......................SV.TDTPPV..........LSPFRLPHRVID
[Hirsutella_rhossiliensis]_XP_044725020.1              VRYYSYG......................AV.IDKPPL..........LSPFRLSYKVID
[Ophiocordyceps_sinensis_CO18]_EQK99704.1              VRYFSYG......................AV.SDAPSL..........LSPFRLSHNVID
[Tolypocladium_capitatum]_PNY29418.1                   VRYFSYG......................AA.MNEPPL..........LSPFRLSHRVIE
[Tolypocladium_ophioglossoides_CBS_100239]_KND888      VRYFSYG......................AV.MNEPPL..........LSPFRLSYRVIG
[Tolypocladium_paradoxum]_POR38551.1                   VRYFSYG......................AV.MREPPL..........LSPFRLSHRVIG
[Drechmeria_coniospora]_ODA83100.1                     VRYFSYG......................AV.VEDLPL..........FSPFRLPQKIIA
[Drechmeria_coniospora]_XP_040657415.1                 VRYFSYG......................AV.VEDLPL..........FSPFRLPQKIIA
[Ophiocordyceps_unilateralis]_PFH57814.1               VRYFSYG......................AV.APSPSL..........FSPFRLPHRIIE
[Fusarium_sp._AF-6]_RSL54391.1                         VHYFSYG......................AA.VERPAL..........LSPFRHPHAVVT
[Fusarium_kuroshium]_RMJ16314.1                        VHYFSYG......................AA.VERPAL..........LSPFRHPHAVMT
[Fusarium_sp._AF-8]_RSL70798.1                         VHYFSYG......................AA.VERPAL..........LSPFRHPHAVMT
[Fusarium_ambrosium]_RSM11997.1                        VHYFSYG......................AA.VERPAL..........LSPFRHPHAVMT
[Fusarium_floridanum]_RSL80024.1                       VHYFSYG......................AA.VERPAL..........LSPFRHPHAVMT
[Fusarium_vanettenii_77-13-4]_XP_003050716.1           VHYFSYG......................AA.VERPAL..........LSPFRHPHAVMT
[Fusarium_solani]_XP_046134414.1                       VHYFSYG......................AA.VERPAL..........LSPFRHPHAVMT
[Fusarium_albosuccineum]_KAF4468415.1                  VRYFSYG......................AA.VEQPAM..........LSPFRHPHAVMT
[Fusarium_decemcellulare]_KAF5011412.1                 VRYFSYG......................AA.VERPAL..........LSPFRHPHAVMT
[Fusarium_sarcochroum]_KAF4964785.1                    VRYFSYG......................AA.VEQPPL..........LSPFRHPYAVMT
[Fusarium_avenaceum]_KAH6968652.1                      VRYFSYG......................AA.VEQPAL..........LSPFRHPHAVMT
[Fusarium_avenaceum]_KIL96274.1                        VRYFSYG......................AA.VEQPAL..........LSPFRHPHAVMT
[Fusarium_avenaceum]_KAG5656265.1                      VRYFSYG......................AA.VEQPAL..........LSPFRHPHAVMT
[Fusarium_tricinctum]_KAH7262197.1                     VRYFSYG......................AA.VEHPAL..........LSPFRHPHAVMT
[Fusarium_heterosporum]_KAF5663380.1                   VRYFSYG......................AA.VEQPAL..........LSPFRHPHAVMM
[Fusarium_sp._MPI-SDFR-AT-0072]_KAH7164412.1           VRYFSYG......................AA.VERPAL..........LSPFRHPHAVMT
[Fusarium_oxysporum_f._sp._rapae]_KAG7414252.1         VRYFSYG......................AA.VERPAL..........LSPFRHPHAVMT
[Fusarium_mangiferae]_XP_041681833.1                   VRYFSYG......................AA.VERPAL..........LSPFRHPHAIMT
[Fusarium_proliferatum]_CVL11341.1                     VRYFSYG......................AA.VERPAL..........LSPFRHPHAIMT
[Fusarium_proliferatum_ET1]_XP_031083673.1             VRYFSYG......................AA.VERPAL..........LSPFRHPHAIMT
[Fusarium_fujikuroi]_SCN78580.1                        VRYFSYG......................AA.VERPAL..........LSPFRHPHAIMT
[Fusarium_fujikuroi_IMI_58289]_XP_023430709.1          VRYFSYG......................AA.VERPAL..........LSPFRHPHAIMT
[Fusarium_sp._NRRL_25303]_KAF5626228.1                 VRYFSYG......................AA.VERPAL..........LSPFRHPHAIMT
[Fusarium_xylarioides]_KAG5746670.1                    VRYFSYG......................AA.VERPAL..........LSPFRHPHAVMT
[Fusarium_xylarioides]_KAG5788219.1                    VRYFSYG......................AA.VERPAL..........LSPFRHPHAVMT
[Fusarium_pseudocircinatum]_KAF5575212.1               VRYFSYG......................AA.VERPAL..........LSPFRHPHAVMT
[Fusarium_pseudoanthophilum]_KAF5575021.1              VRYFSYG......................AA.VERPAL..........LSPFRHPHAVMT
[Fusarium_verticillioides]_RBQ71412.1                  VRYFSYG......................AA.VERPAL..........LSPFRHPHAVMT
[Fusarium_verticillioides_7600]_XP_018746479.1         VRYFSYG......................AA.VERPAL..........LSPFRHPHAVMT
[Fusarium_oxysporum_NRRL_32931]_XP_031039788.1         VRYFSYG......................AA.VERPAL..........LSPFRHPHAVMT
[Fusarium_oxysporum_f._sp._lycopersici_4287]_XP_0      VRYFSYG......................AA.VERPAL..........LSPFRHPHAVMT
[Fusarium_oxysporum_f._sp._cepae]_RKK20771.1           VRYFSYG......................AA.VERPAL..........LSPFRHPHAVMT
[Fusarium_redolens]_XP_046051341.1                     VRYFSYG......................AA.VERPAL..........LSPFRHPHAVMT
[Fusarium_austroafricanum]_KAF4458007.1                VRYFSYG......................AA.VERPAL..........LSPFRHPHAVMT
[Fusarium_flagelliforme]_XP_045986929.1                VRYFSYG......................AA.VERPAL..........LSPFRHPHAVMT
[Fusarium_sp._FIESC_RH6]_KAI1056250.1                  VRYFSYG......................AA.VERPAL..........LSPFRHPHAVMT
[Fusarium_sporotrichioides]_RGP72309.1                 VRYFSYG......................AA.VERPAL..........LSPFRHPHAVMT
[Fusarium_poae]_XP_044713592.1                         VRYFSYG......................AA.VERPAL..........LSPFRHPHAVMT
[Fusarium_venenatum]_XP_025590637.1                    VRYFSYG......................AA.VERPAL..........LSPFRHPHAVMT
[Fusarium_pseudograminearum]_KAF0641638.1              VRYFSYG......................AA.VERPAL..........LSPFRHPHAVMT
[Fusarium_graminearum]_CAG1993854.1                    VRYFSYG......................AA.VERPAL..........LSPFRHPHAVMT
[Fusarium_graminearum]_PCD31638.1                      VRYFSYG......................AA.VERPAL..........LSPFRHPHAVMT
[Fusarium_graminearum]_CZS78629.1                      VRYFSYG......................AA.VERPAL..........LSPFRHPHAVMT
[Fusarium_graminearum]_CAF3606094.1                    VRYFSYG......................AA.VERPAL..........LSPFRHPHAVMT
[Fusarium_graminearum_PH-1]_XP_011318944.1             VRYFSYG......................AA.VERPAL..........LSPFRHPHAVMT
[Fusarium_culmorum]_QPC68571.1                         VRYFSYG......................AA.VERPAL..........LSPFRHPHAVMT
[Fusarium_pseudograminearum]_QPC77441.1                VRYFSYG......................AA.VERPAL..........LSPFRHPHAVMT
[Fusarium_longipes]_RGP79158.1                         VRYFSYG......................AA.VERPAL..........LSPFRHPHAIMA
[Ilyonectria_destructans]_KAH7011941.1                 VRYFSYG......................AAFVKTPPL..........LNPFRYPHGVLA
[Ilyonectria_sp._MPI-CAGE-AT-0026]_KAH6985660.1        VRYFSYG......................AAFVKTPPL..........LNPFRYPHGVLA
[Ilyonectria_robusta]_XP_046104990.1                   VRYFSYG......................AAFVKTPPL..........LSPFRYPHGVLA
[Dactylonectria_estremocensis]_KAH7149692.1            VRYFSYG......................AAFVETPPL..........LSPFRYPHGVLT
[Dactylonectria_macrodidyma]_KAH7148923.1              VRYFSYG......................AAFVGTPPL..........LSPFRYPHSILT
[Thelonectria_olida]_KAH6899778.1                      VQYFSYG......................AALEDVPPL..........LSPFRHSHGVIA
[Stachybotrys_chartarum_IBT_7711]_KEY69997.1           VQYYSYG......................ASLTQPPPL..........LSPFRQSHRIIS
[Stachybotrys_chartarum_IBT_40293]_KFA50745.1          VQYYSYG......................ASLTQPPPL..........LSPFRQSHRIIS
[Stachybotrys_chlorohalonata_IBT_40285]_KFA62815.      VQYYSYG......................ASLTQPPPL..........LSPFRQSHRIIS
[Purpureocillium_lilacinum]_XP_018174914.1             VRYFSYG......................AA.LHTAQLP........MLSPFRLSHGVIR
[Clonostachys_solani]_CAH0059244.1                     VRYFSYG......................AM.MDEPAL..........LSPFRQPWRLIE
[Clonostachys_rosea]_VUC36940.1                        VRYFSYG......................AM.MDEPAL..........LSPFRQPWRLIE
[Clonostachys_rhizophaga]_CAH0026503.1                 VRYFSYG......................AM.MDEPAL..........LSPFRQPWRLID
[Emericellopsis_atlantica]_XP_046114137.1              TRYFSYG......................AS.TTEPPL..........LSPFRQSWHAIT
[Acremonium_chrysogenum_ATCC_11550]_KFH46845.1         VQYFSYG......................AR.CDEPPL..........LSPFRRSWQLIR
[Pochonia_chlamydosporia_170]_XP_018148143.1           VRYFSYG......................AM.MAPPPL..........LSPFRIPKRIIE
[Epichloe_festucae_Fl1]_QPG98063.1                     VRYFSYG......................AV.MDPPPL..........LSPFRLPQRIVE
[Colletotrichum_incanum]_KZL82863.1                    VRYFSYG......................AC.TDRPPL..........LSPFRQSHRILE
[Colletotrichum_simmondsii]_KXH39849.1                 VRYFSYG......................AC.TDRPPL..........LSPFRQSHWVIE
[Colletotrichum_sojae]_KAF6819546.1                    VRYFSYG......................AC.TGTPPL..........LSPFRQSHRILD
[Bacillus_licheniformis]_CAB95850.2_RN2                .............................SA.............................
[Trichosporon_asahii_var._asahii]_AGN98126.1_TaLi      DRLHEGG......................VT.VPGPTYTNIMSKLDLQVTPY..TSGRID
```

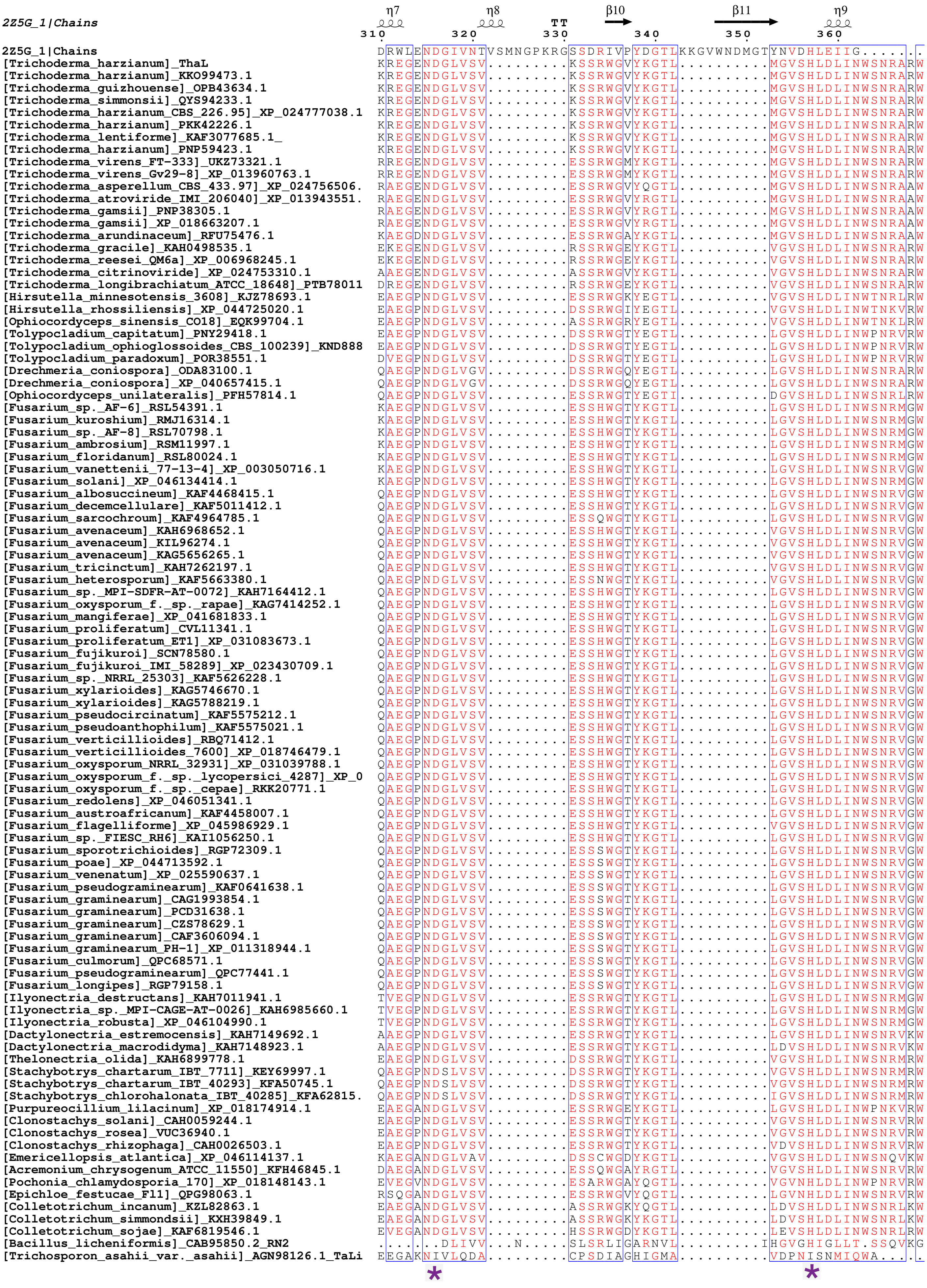
2Z5G_1|Chains
η7
η8
TT
β10
β11
η9
310
320
330
340
350
360

```
                                                                         α13
2Z5G_1|Chains                                              TT     QQQQQQQQQQQQQQQ
                                                                370      380

2Z5G_1|Chains                                        .....VDPNPSF.DIRAFYLRLAEQLASLQP.............................
[Trichoderma_harzianum]_ThaL                         TVRE.WMGMRRTF.NAVAFYLDVADMLAKEGL.............................
[Trichoderma_harzianum]_KKO99473.1                   TVRE.WMGMRRTF.NAVAFYLDVADMLAKEGL.............................
[Trichoderma_guizhouense]_OPB43634.1                 TVRE.WMGMRRTF.NAVAFYLDVADMLAKEGL.............................
[Trichoderma_simmonsii]_QYS94233.1                   TVRE.WMGMRRTF.NAVAFYLDVADMLAKEGL.............................
[Trichoderma_harzianum_CBS_226.95]_XP_024777038.1    TVRE.WMGMRRTF.NAVAFYLDVADMLAKEGL.............................
[Trichoderma_harzianum]_PKK42226.1                   TVRE.WMGMRRTF.NAVAFYLDVADMLAKEGL.............................
[Trichoderma_lentiforme]_KAF3077685.1_               TVRE.WMGMRRTF.NAVAFYLDVADMLAKEGL.............................
[Trichoderma_harzianum]_PNP59423.1                   TVRE.WMGMRRTF.NAVAFYLDVADMLAKEGL.............................
[Trichoderma_virens_FT-333]_UKZ73321.1               TVRE.WMGMRRTF.NAVAFYLDVVDMLAKEGL.............................
[Trichoderma_virens_Gv29-8]_XP_013960763.1           TVRE.WMGMRRTF.NAVAFYLDVVDMLAKEGL.............................
[Trichoderma_asperellum_CBS_433.97]_XP_024756506.    TMRE.WMGMKRTF.NAVAFYLDVADMLAKEGL.............................
[Trichoderma_atroviride_IMI_206040]_XP_013943551.    TVRE.WMGMKRTF.NAVAFYLDVADMLAKEGL.............................
[Trichoderma_gamsii]_PNP38305.1                      TVRE.WMGMKRTF.NAVAFYLDVADMLAKEGL.............................
[Trichoderma_gamsii]_XP_018663207.1                  TVRE.WMGMKRTF.NAVAFYLDVADMLAKEGL.............................
[Trichoderma_arundinaceum]_RFU75476.1                TMRE.WMGMKRTF.NAVAFYLDVADMLAKEGL.............................
[Trichoderma_gracile]_KAH0498535.1                   AVRE.WMGMKRTF.NAVAFYLDVADMLAKEGL.............................
[Trichoderma_reesei_QM6a]_XP_006968245.1             AVRE.WMGMKRTF.NAVAFYLDVADMLAKEGL.............................
[Trichoderma_citrinoviride]_XP_024753310.1           AVRE.WMGMRRNF.NAVAFYLDVADMLAKEGL.............................
[Trichoderma_longibrachiatum_ATCC_18648]_PTB78011    AVRE.WMGMRRTF.NAVAFYLDVADMLAKEGL.............................
[Hirsutella_minnesotensis_3608]_KJZ78693.1           TVRE.WVGIKRTF.NAIAFYLAITDMLAKQGL.............................
[Hirsutella_rhossiliensis]_XP_044725020.1            TVRE.WMGYKKTF.NAIAFYLAITDMLAREGL.............................
[Ophiocordyceps_sinensis_CO18]_EQK99704.1            MMRE.CIGIKRTF.NAIAFYLAITDMLAREGL.............................
[Tolypocladium_capitatum]_PNY29418.1                 TVRE.WMGMKRTF.NAIAFYLGITDMLAKEGL.............................
[Tolypocladium_ophioglossoides_CBS_100239]_KND888    TVRE.WMGRKRTF.NAIAFYLGITDMLAKEGL.............................
[Tolypocladium_paradoxum]_POR38551.1                 TVRE.WMGTKRAF.NAIAFYLGITDMLAKAGL.............................
[Drechmeria_coniospora]_ODA83100.1                   TVRE.WMGVKRNF.NAVAFYLGIADMLAREGL.............................
[Drechmeria_coniospora]_XP_040657415.1               TVRE.WMGVKRNF.NAVAFYLGIADMLAREGL.............................
[Ophiocordyceps_unilateralis]_PFH57814.1             TVRE.WMGVRRNF.NAIAFYLAITDRLAKEGF.............................
[Fusarium_sp._AF-6]_RSL54391.1                       TVRE.WMGIRRTF.NAIAFYLDIADMLAKEGY.............................
[Fusarium_kuroshium]_RMJ16314.1                      TVRE.WMGIRRTF.NAIAFYLDIADMLAKEGY.............................
[Fusarium_sp._AF-8]_RSL70798.1                       TVRE.WMGIRRTF.NAIAFYLDIADMLAKEGY.............................
[Fusarium_ambrosium]_RSM11997.1                      TVRE.WMGIRRTF.NAIAFYLDIADMLAKEGY.............................
[Fusarium_floridanum]_RSL80024.1                     TVRE.WMGIRRTF.NAIAFYLDIADMLAKEGY.............................
[Fusarium_vanettenii_77-13-4]_XP_003050716.1         TVRE.WMGIRRTF.NAIAFYLDIADMLAKEGY.............................
[Fusarium_solani]_XP_046134414.1                     TVRE.WMGIRRTF.NAIAFYLDIADMLAKEGY.............................
[Fusarium_albosuccineum]_KAF4468415.1                TVRE.WMGIKRTF.NAIAFYLDIADMLAKEGF.............................
[Fusarium_decemcellulare]_KAF5011412.1               TVRE.WMGIKRTF.NAIAFYLDIADMLAKEGF.............................
[Fusarium_sarcochroum]_KAF4964785.1                  TVRE.WMGIKKNF.NAIAYYLDIADMLAKEGY.............................
[Fusarium_avenaceum]_KAH6968652.1                    TVRE.WMGIKKNF.NAIAFYLDIADMLAKEGY.............................
[Fusarium_avenaceum]_KIL96274.1                      TVRE.WMGIKKNF.NAIAFYLDIADMLAKEGY.............................
[Fusarium_avenaceum]_KAG5656265.1                    TVRE.WMGIKKNF.NAIAFYLDIADMLAKEGY.............................
[Fusarium_tricinctum]_KAH7262197.1                   TVRE.WMGIKKNF.NAIAFYLDIADMLAKEGY.............................
[Fusarium_heterosporum]_KAF5663380.1                 TVRE.WMGIKRNF.NAIAFYLDIADMLAKEGY.............................
[Fusarium_sp._MPI-SDFR-AT-0072]_KAH7164412.1         TVRE.WMGIKKNF.NAIAFYLDIADMLAKEGY.............................
[Fusarium_oxysporum_f._sp._rapae]_KAG7414252.1       TVRE.WMGIKKNF.NAIAFYLDIADMLAKEGY.............................
[Fusarium_mangiferae]_XP_041681833.1                 TVRE.WMGIKKNF.NAIAFYLDIADMLAKEGY.............................
[Fusarium_proliferatum]_CVL11341.1                   TVRE.WMGIKKNF.NAIAFYLDIADMLAKEGY.............................
[Fusarium_proliferatum_ET1]_XP_031083673.1           TVRE.WMGIKKNF.NAIAFYLDIADMLAKEGY.............................
[Fusarium_fujikuroi]_SCN78580.1                      TVRE.WMGIKKNF.NAIAFYLDIADMLAKEGY.............................
[Fusarium_fujikuroi_IMI_58289]_XP_023430709.1        TVRE.WMGIKKNF.NAIAFYLDIADMLAKEGY.............................
[Fusarium_sp._NRRL_25303]_KAF5626228.1               TVRE.WMGIKKNF.NAIAFYLDIADMLAKEATLPRTLDENPPSSTFVPRDPVIALAVDKR
[Fusarium_xylarioides]_KAG5746670.1                  TVRE.WMGIKKNF.NAIAFYLDIADMLAKEGY.............................
[Fusarium_xylarioides]_KAG5788219.1                  TVRE.WMGIKKNF.NAIAFYLDIADMLAKEGY.............................
[Fusarium_pseudocircinatum]_KAF5575212.1             TVRE.WMGIKKNF.NAIAFYLDIADMLAKEATLPRTLDENPPSSTFVPRDPVIALAVDKR
[Fusarium_pseudoanthophilum]_KAF5575021.1            TVRE.WMGIKKNF.NAIAFYLDIADMLAKEATLPRTLDENPPSSTFAPRDPVIALAVDKR
[Fusarium_verticillioides]_RBQ71412.1                TVRE.WVGIKKNF.NAIAFYLDIADMLAKEGY.............................
[Fusarium_verticillioides_7600]_XP_018746479.1       TVRE.WVGIKKNF.NAIAFYLDIADMLAKEGY.............................
[Fusarium_oxysporum_NRRL_32931]_XP_031039788.1       TVRE.WMGIKRNF.NAIAFYLDIADMLAKEGY.............................
[Fusarium_oxysporum_f._sp._lycopersici_4287]_XP_0    TVRE.WMGIKRNF.NAIAFYLDIADMLAKEGY.............................
[Fusarium_oxysporum_f._sp._cepae]_RKK20771.1         TVRE.WMGIKRNF.NAIAFYLDIADMLAKEGY.............................
[Fusarium_redolens]_XP_046051341.1                   TVRE.WMGIKKKF.NAIAFYLDIADMLAKEGY.............................
[Fusarium_austroafricanum]_KAF4458007.1              TVRE.WMGIKKNF.NAIAFYLDIADMLAKEGY.............................
[Fusarium_flagelliforme]_XP_045986929.1              TVRE.WMGIKKNF.NAIAFYLDIADMLAKEGF.............................
[Fusarium_sp._FIESC_RH6]_KAI1056250.1                TVRE.WMGIKKNF.NAIAFYLDIADMLAKEGF.............................
[Fusarium_sporotrichioides]_RGP72309.1               TVRE.WMGIKKNF.NAIAFYLDIADMLAKEGF.............................
[Fusarium_poae]_XP_044713592.1                       TVRE.WMGIKKNF.NAIAFYLDIADMLAKEGF.............................
[Fusarium_venenatum]_XP_025590637.1                  TVRE.WMGIKKNF.NAIAFYLDIADMLAKEGF.............................
[Fusarium_pseudograminearum]_KAF0641638.1            TVRE.WMGIKKNF.NAIAFYLDIADMLAKEGF.............................
[Fusarium_graminearum]_CAG1993854.1                  TVRE.WMGIKKNF.NAIAFYLDIADMLAKEGF.............................
[Fusarium_graminearum]_PCD31638.1                    TVRE.WMGIKKNF.NAIAFYLDIADMLAKEGF.............................
[Fusarium_graminearum]_CZS78629.1                    TVRE.WMGIKKNF.NAIAFYLDIADMLAKEGF.............................
[Fusarium_graminearum]_CAF3606094.1                  TVRE.WMGIKKNF.NAIAFYLDIADMLAKEGF.............................
[Fusarium_graminearum_PH-1]_XP_011318944.1           TVRE.WMGIKKNF.NAIAFYLDIADMLAKEGF.............................
[Fusarium_culmorum]_QPC68571.1                       TVRE.WMGIKKNF.NAIAFYLDIADMLAKEGF.............................
[Fusarium_pseudograminearum]_QPC77441.1              TVRE.WMGIKKNF.NAIAFYLDIADMLAKEGF.............................
[Fusarium_longipes]_RGP79158.1                       TVRE.WMGIKKNF.NAIAFYLDIADMLAKEGF.............................
[Ilyonectria_destructans]_KAH7011941.1               TVRE.WMGMKRTF.NAIAFYLDMADMLAKEGL.............................
[Ilyonectria_sp._MPI-CAGE-AT-0026]_KAH6985660.1      TVRE.WMGMKRTF.NAIAFYLDMADMLAKEGL.............................
[Ilyonectria_robusta]_XP_046104990.1                 TVRE.WMGMKRTF.NAIAFYLDMADMLAKEGL.............................
[Dactylonectria_estremocensis]_KAH7149692.1          TMRE.WMGMKRNF.NAIAFYLDMADMLAKEDL.............................
[Dactylonectria_macrodidyma]_KAH7148923.1            TVRE.WIGMKRNF.NAIAFYLDMADMLAKEGL.............................
[Thelonectria_olida]_KAH6899778.1                    TVRE.WMGMKRNF.NAIAFYLDIADMLAKEGL.............................
[Stachybotrys_chartarum_IBT_7711]_KEY69997.1         TLRE.LMGMKKTF.NAVAFYLDIADMLAKEGL.............................
[Stachybotrys_chartarum_IBT_40293]_KFA50745.1        TLRE.LMGMKKTF.NAVAFYLDIADMLAKEGL.............................
[Stachybotrys_chlorohalonata_IBT_40285]_KFA62815.    TLRE.LMGMKKTF.NAVAFYLDIADMLAKEGL.............................
[Purpureocillium_lilacinum]_XP_018174914.1           TVRE.WMGIKRNF.NAVAFYLDIADMLAKEGY.............................
[Clonostachys_solani]_CAH0059244.1                   TVRE.WMGVKRTF.NAIAFYLDIADMLAKEGL.............................
[Clonostachys_rosea]_VUC36940.1                      TVRE.WMGVKRTF.NAIAFYLDIADMLAKEGL.............................
[Clonostachys_rhizophaga]_CAH0026503.1               TVRE.WMGVKRTF.NAIAFYLDIADMLAKEGL.............................
[Emericellopsis_atlantica]_XP_046114137.1            MVRE.WMGMKRTF.NAIAFYLDVADMLAKEGL.............................
[Acremonium_chrysogenum_ATCC_11550]_KFH46845.1       TLRE.WMGMKRNF.NAIAFYLDIADMLAKEGY.............................
[Pochonia_chlamydosporia_170]_XP_018148143.1         MVRG.WIGVRKRF.NAVAFYLGIADMLAREGL.............................
[Epichloe_festucae_Fl1]_QPG98063.1                   MVKR.WMGVRRGF.NAVAFYLGIADMLAKEGL.............................
[Colletotrichum_incanum]_KZL82863.1                  TLRKVWMGQARTF.NAVAFYLDIADMLAKEGL.............................
[Colletotrichum_simmondsii]_KXH39849.1               TLRKVWMGQTRTF.NAVAFYLDIADMLAKEGL.............................
[Colletotrichum_sojae]_KAF6819546.1                  TLRKVWMGQTRTF.NAVAFYLDIADMLAKEGL.............................
[Bacillus_licheniformis]_CAB95850.2_RN2              YIKEGLNGGGQNT.N...............................................
[Trichosporon_asahii_var._asahii]_AGN98126.1_TaLi    .....IDGRNGPLPKCKPFFLAI.......................................
```

2Z5G_1|Chains

```
2Z5G_1|Chains                                          ...................................
[Trichoderma_harzianum]_ThaL                           ...................................
[Trichoderma_harzianum]_KKO99473.1                     ...................................
[Trichoderma_guizhouense]_OPB43634.1                   ...................................
[Trichoderma_simmonsii]_QYS94233.1                     ...................................
[Trichoderma_harzianum_CBS_226.95]_XP_024777038.1      ...................................
[Trichoderma_harzianum]_PKK42226.1                     ...................................
[Trichoderma_lentiforme]_KAF3077685.1_                 ...................................
[Trichoderma_harzianum]_PNP59423.1                     ...................................
[Trichoderma_virens_FT-333]_UKZ73321.1                 ...................................
[Trichoderma_virens_Gv29-8]_XP_013960763.1             ...................................
[Trichoderma_asperellum_CBS_433.97]_XP_024756506.      ...................................
[Trichoderma_atroviride_IMI_206040]_XP_013943551.      ...................................
[Trichoderma_gamsii]_PNP38305.1                        ...................................
[Trichoderma_gamsii]_XP_018663207.1                    ...................................
[Trichoderma_arundinaceum]_RFU75476.1                  ...................................
[Trichoderma_gracile]_KAH0498535.1                     ...................................
[Trichoderma_reesei_QM6a]_XP_006968245.1               ...................................
[Trichoderma_citrinoviride]_XP_024753310.1             ...................................
[Trichoderma_longibrachiatum_ATCC_18648]_PTB78011      ...................................
[Hirsutella_minnesotensis_3608]_KJZ78693.1             ...................................
[Hirsutella_rhossiliensis]_XP_044725020.1              ...................................
[Ophiocordyceps_sinensis_CO18]_EQK99704.1              ...................................
[Tolypocladium_capitatum]_PNY29418.1                   ...................................
[Tolypocladium_ophioglossoides_CBS_100239]_KND888      ...................................
[Tolypocladium_paradoxum]_POR38551.1                   ...................................
[Drechmeria_coniospora]_ODA83100.1                     ...................................
[Drechmeria_coniospora]_XP_040657415.1                 ...................................
[Ophiocordyceps_unilateralis]_PFH57814.1               ...................................
[Fusarium_sp._AF-6]_RSL54391.1                         ...................................
[Fusarium_kuroshium]_RMJ16314.1                        ...................................
[Fusarium_sp._AF-8]_RSL70798.1                         ...................................
[Fusarium_ambrosium]_RSM11997.1                        ...................................
[Fusarium_floridanum]_RSL80024.1                       ...................................
[Fusarium_vanettenii_77-13-4]_XP_003050716.1           ...................................
[Fusarium_solani]_XP_046134414.1                       ...................................
[Fusarium_albosuccineum]_KAF4468415.1                  ...................................
[Fusarium_decemcellulare]_KAF5011412.1                 ...................................
[Fusarium_sarcochroum]_KAF4964785.1                    ...................................
[Fusarium_avenaceum]_KAH6968652.1                      ...................................
[Fusarium_avenaceum]_KIL96274.1                        ...................................
[Fusarium_avenaceum]_KAG5656265.1                      ...................................
[Fusarium_tricinctum]_KAH7262197.1                     ...................................
[Fusarium_heterosporum]_KAF5663380.1                   ...................................
[Fusarium_sp._MPI-SDFR-AT-0072]_KAH7164412.1           ...................................
[Fusarium_oxysporum_f._sp._rapae]_KAG7414252.1         ...................................
[Fusarium_mangiferae]_XP_041681833.1                   ...................................
[Fusarium_proliferatum]_CVL11341.1                     ...................................
[Fusarium_proliferatum_ET1]_XP_031083673.1             ...................................
[Fusarium_fujikuroi]_SCN78580.1                        ...................................
[Fusarium_fujikuroi_IMI_58289]_XP_023430709.1          ...................................
[Fusarium_sp._NRRL_25303]_KAF5626228.1                 GIAEGRNRQQLTGIRGHSRSPDVSQKAPRRNTTST
[Fusarium_xylarioides]_KAG5746670.1                    ...................................
[Fusarium_xylarioides]_KAG5788219.1                    ...................................
[Fusarium_pseudocircinatum]_KAF5575212.1               ...................................
[Fusarium_pseudoanthophilum]_KAF5575021.1              GIGEGRNRQHLTGIRGHSRSPDVSQKAPRRNTTST
[Fusarium_verticillioides]_RBQ71412.1                  ...................................
[Fusarium_verticillioides_7600]_XP_018746479.1         ...................................
[Fusarium_oxysporum_NRRL_32931]_XP_031039788.1         ...................................
[Fusarium_oxysporum_f._sp._lycopersici_4287]_XP_0      ...................................
[Fusarium_oxysporum_f._sp._cepae]_RKK20771.1           ...................................
[Fusarium_redolens]_XP_046051341.1                     ...................................
[Fusarium_austroafricanum]_KAF4458007.1                ...................................
[Fusarium_flagelliforme]_XP_045986929.1                ...................................
[Fusarium_sp._FIESC_RH6]_KAI1056250.1                  ...................................
[Fusarium_sporotrichioides]_RGP72309.1                 ...................................
[Fusarium_poae]_XP_044713592.1                         ...................................
[Fusarium_venenatum]_XP_025590637.1                    ...................................
[Fusarium_pseudograminearum]_KAF0641638.1              ...................................
[Fusarium_graminearum]_CAG1993854.1                    ...................................
[Fusarium_graminearum]_PCD31638.1                      ...................................
[Fusarium_graminearum]_CZS78629.1                      ...................................
[Fusarium_graminearum]_CAF3606094.1                    ...................................
[Fusarium_graminearum_PH-1]_XP_011318944.1             ...................................
[Fusarium_culmorum]_QPC68571.1                         ...................................
[Fusarium_pseudograminearum]_QPC77441.1                ...................................
[Fusarium_longipes]_RGP79158.1                         ...................................
[Ilyonectria_destructans]_KAH7011941.1                 ...................................
[Ilyonectria_sp._MPI-CAGE-AT-0026]_KAH6985660.1        ...................................
[Ilyonectria_robusta]_XP_046104990.1                   ...................................
[Dactylonectria_estremocensis]_KAH7149692.1            ...................................
[Dactylonectria_macrodidyma]_KAH7148923.1              ...................................
[Thelonectria_olida]_KAH6899778.1                      ...................................
[Stachybotrys_chartarum_IBT_7711]_KEY69997.1           ...................................
[Stachybotrys_chartarum_IBT_40293]_KFA50745.1          ...................................
[Stachybotrys_chlorohalonata_IBT_40285]_KFA62815.      ...................................
[Purpureocillium_lilacinum]_XP_018174914.1             ...................................
[Clonostachys_solani]_CAH0059244.1                     ...................................
[Clonostachys_rosea]_VUC36940.1                        ...................................
[Clonostachys_rhizophaga]_CAH0026503.1                 ...................................
[Emericellopsis_atlantica]_XP_046114137.1              ...................................
[Acremonium_chrysogenum_ATCC_11550]_KFH46845.1         ...................................
[Pochonia_chlamydosporia_170]_XP_018148143.1           ...................................
[Epichloe_festucae_Fl1]_QPG98063.1                     ...................................
[Colletotrichum_incanum]_KZL82863.1                    ...................................
[Colletotrichum_simmondsii]_KXH39849.1                 ...................................
[Colletotrichum_sojae]_KAF6819546.1                    ...................................
[Bacillus_licheniformis]_CAB95850.2_RN2                ...................................
[Trichosporon_asahii_var._asahii]_AGN98126.1_TaLi      ...................................
```
