# Supplementary material for: Identification of a Novel Lipase with AHSMG Pentapeptide in Hypocreales and Glomerellales Filamentous Fungi
Source: Int J Mol Sci. 2022 Aug 19;23(16):9367. doi: 10.3390/ijms23169367 (PMC9408867; doi:10.3390/ijms23169367)
Supplement: Supplementary file 1 [file ijms-23-09367-s001.zip › Figure S4B.Alignment of ThaL and its top ten homologs with TaLipA, RN2 and 2Z5G.pdf]

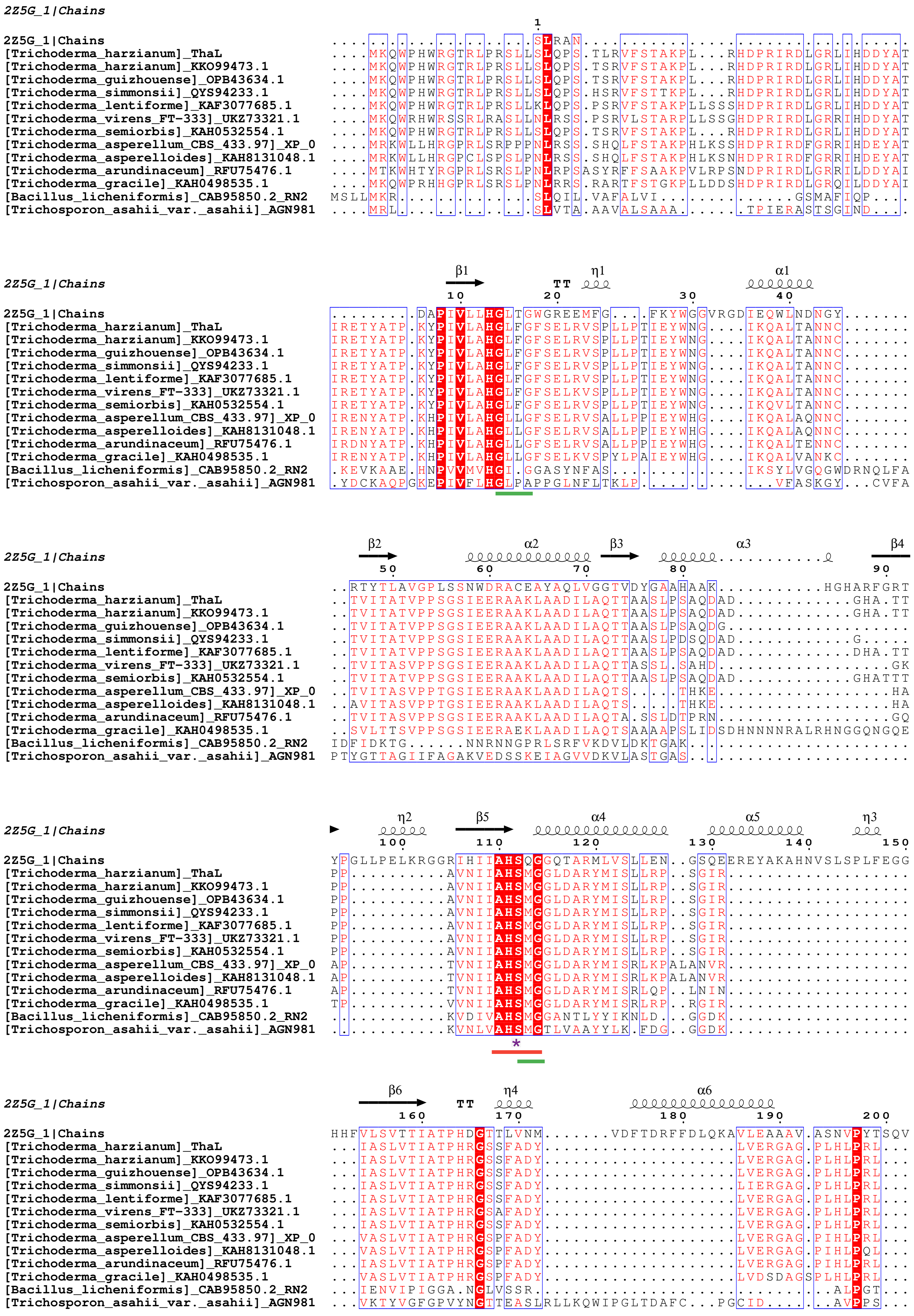
2Z5G_1|Chains
2Z5G_1|Chains
[Trichoderma_harzianum]_ThaL
[Trichoderma_harzianum]_KKO99473.1
[Trichoderma_guizhouense]_OPB43634.1
[Trichoderma_simmonsii]_QYS94233.1
[Trichoderma_lentiforme]_KAF3077685.1
[Trichoderma_virens_FT-333]_UKZ73321.1
[Trichoderma_semiorbis]_KAH0532554.1
[Trichoderma_asperellum_CBS_433.97]_XP_0
[Trichoderma_asperelloides]_KAH8131048.1
[Trichoderma_arundinaceum]_RFU75476.1
[Trichoderma_gracile]_KAH0498535.1
[Bacillus_licheniformis]_CAB95850.2_RN2
[Trichosporon_asahii_var._asahii]_AGN981
β1 η1 α1 β2 α2 β3 α3 β4 η2 β5 α4 α5 η3 β6 η4 α6
TT

```
                                              η5          TT          α7          α8          α9          α10          T
2Z5G_1|Chains                                      210          220          230          240          250
2Z5G_1|Chains                               YDFKLDQWGLRRQPG...ESFDHYFERLKRSPVWTSTDTARYDLSVSGAEKLNQWVQASP
[Trichoderma_harzianum]_ThaL                .......YGVIRRAGLGTSAFGQLTTRYMR...................EEFNPRVRDDE
[Trichoderma_harzianum]_KKO99473.1          .......YGVIRRAGLGTSAFGQLTTRYMR...................EEFNPRVRDDE
[Trichoderma_guizhouense]_OPB43634.1        .......YGVIRRAGLGTSAFGQLTTRYMR...................EEFNPRVRDDE
[Trichoderma_simmonsii]_QYS94233.1          .......YGVIRRAGLGTSAFGQLTTRYMR...................EEFNPRVRDDE
[Trichoderma_lentiforme]_KAF3077685.1       .......YGVIRRAGLGTSAFGQLTTRYMR...................EEFNPRVRDEE
[Trichoderma_virens_FT-333]_UKZ73321.1      .......YGVIRRAGLGTSAFGQLTTRYMR...................DEFNPRVRDDE
[Trichoderma_semiorbis]_KAH0532554.1        .......YGVIRRAGLGTSAFGQLTTRYMR...................EEFNPRVRDDE
[Trichoderma_asperellum_CBS_433.97]_XP_0    .......YGIIRRAGLGTSAFGQLTTRYMR...................EEFNPRVRDDE
[Trichoderma_asperelloides]_KAH8131048.1    .......YGIIRRAGLGTSAFGQLTTRYMR...................EEFNPRVRDDE
[Trichoderma_arundinaceum]_RFU75476.1       .......YGIIRRAGLGTSAFGQLTTRYMR...................EEFNPRVQDDE
[Trichoderma_gracile]_KAH0498535.1          .......YSVIRRAGLGTSAFGQLTTRYMR...................DEFNPRVPDDE
[Bacillus_licheniformis]_CAB95850.2_RN2     .....DPNQKI...........LYTSVYYSS..........ADLIV...........
[Trichosporon_asahii_var._asahii]_AGN981    .....DVIDRLHEGGV..TVPGPTYTNIMSK..........LDLQV...........

                                                   β7        β8        β9    TT     α11     α12      η6      η7
2Z5G_1|Chains                               T  
                                            260          270          280          290          300          310
2Z5G_1|Chains                               NTYYLSFSTERTYRGALTGNHYPELGMNAFSAVVCAPFLGSYRNPTLGIDDRWLENDGIV
[Trichoderma_harzianum]_ThaL                AVRYFSY......................GAAIDEPSLLGAFRLPHGVVDKREGENDGLV
[Trichoderma_harzianum]_KKO99473.1          AVRYFSY......................GAAIDEPSLLGAFRLPHGVVDKREGENDGLV
[Trichoderma_guizhouense]_OPB43634.1        GVRYFSY......................GAAIDEPSLLGAFRLPHGVVDKREGENDGLV
[Trichoderma_simmonsii]_QYS94233.1          GVRYFSY......................GAAIDEPSLLGAFRLPHGVVDKREGENDGLV
[Trichoderma_lentiforme]_KAF3077685.1       GVRYFSY......................GAAIDEPSLLGAFRLPHGVVDKREGENDGLV
[Trichoderma_virens_FT-333]_UKZ73321.1      AVRYFSY......................GAAIDEPSLLGAFRLPHNVVDRREGENDGLV
[Trichoderma_semiorbis]_KAH0532554.1        AVRYFSY......................GAAIDEPPLLGAFRLPHGVVDKREGENDGLP
[Trichoderma_asperellum_CBS_433.97]_XP_0    SVRYFSY......................GAAIDEPSLLGAFRLPYGVVGRAEGENDGLV
[Trichoderma_asperelloides]_KAH8131048.1    SVRYFSY......................GAAIGEPSLLGAFRLPYGVVGRAEGENDGLV
[Trichoderma_arundinaceum]_RFU75476.1       SVRYFSY......................GAAIDEPSLLGAFRLPHGVVSKAEGDNDGLV
[Trichoderma_gracile]_KAH0498535.1          RVRYFSY......................GAMIDEPSLLGAFRVPYGVIWEKEGENDGLV
[Bacillus_licheniformis]_CAB95850.2_RN2     .VNSLS.......................RLI........................GAR
[Trichosporon_asahii_var._asahii]_AGN981    .TPYTS.......................GRIDE...................EGAK
                                                                                                          *

                                               η8        TT      β10          β11          η9         TT
2Z5G_1|Chains                                                                                              .......
                                            320          330          340          350          360          370
2Z5G_1|Chains                               NTVSMNGPKRGSSDRIVPYDGTLKKGVWNDMGTYNVDHLEIIGVDPNPSFDIR.......
[Trichoderma_harzianum]_ThaL                SV.........KSSRWGVYKGTL..........MGVSHLDLINWSNRARWTVREWM.GMR
[Trichoderma_harzianum]_KKO99473.1          SV.........KSSRWGVYKGTL..........MGVSHLDLINWSNRARWTVREWM.GMR
[Trichoderma_guizhouense]_OPB43634.1        SV.........KSSRWGVYKGTL..........MGVSHLDLINWSNRARWTVREWM.GMR
[Trichoderma_simmonsii]_QYS94233.1          SV.........KSSRWGVYKGTL..........MGVSHLDLINWSNRARWTVREWM.GMR
[Trichoderma_lentiforme]_KAF3077685.1       SV.........KSSRWGVYKGTL..........MGVSHLDLINWSNRARWTVREWM.GMR
[Trichoderma_virens_FT-333]_UKZ73321.1      SV.........ESSRWGMYKGTL..........MGVSHLDLINWSNRARWTVREWM.GMR
[Trichoderma_semiorbis]_KAH0532554.1        .................................FGFDQL..........VESREVD.G..
[Trichoderma_asperellum_CBS_433.97]_XP_0    SV.........ESSRWGVYQGTL..........MGVSHLDLINWSNRAAWTMREWM.GMK
[Trichoderma_asperelloides]_KAH8131048.1    SV.........ESSRWGVYQGTL..........MGVSHLDLINWSNRAAWTMREWM.GMK
[Trichoderma_arundinaceum]_RFU75476.1       SV.........ESSRWGAYKGTL..........MGVSHLDLINWSNRAAWTMREWM.GMK
[Trichoderma_gracile]_KAH0498535.1          SV.........RSSRWGEYKGTL..........VGVSHLDLIN.................
[Bacillus_licheniformis]_CAB95850.2_RN2     NVLIHG.............................VGHIGLLT.SSQVKGYIKEGLNGGG
[Trichosporon_asahii_var._asahii]_AGN981    NIVLQDA...CPSDI....................AGHIGMAV.DPNISNMIQWAIDGRN
                                                                                   *

                                                 α13
2Z5G_1|Chains                               .......
                                                  380
2Z5G_1|Chains                               .......AFYLRLAEQLASLQP.......
[Trichoderma_harzianum]_ThaL                RTFNAV.AFYLDVADMLAKEGL.......
[Trichoderma_harzianum]_KKO99473.1          RTFNAV.AFYLDVADMLAKEGL.......
[Trichoderma_guizhouense]_OPB43634.1        RTFNAV.AFYLDVADMLAKEGL.......
[Trichoderma_simmonsii]_QYS94233.1          RTFNAV.AFYLDVADMLAKEGL.......
[Trichoderma_lentiforme]_KAF3077685.1       RTFNAV.AFYLDVADMLAKEGL.......
[Trichoderma_virens_FT-333]_UKZ73321.1      RTFNAV.AFYLDVVDMLAKEGL.......
[Trichoderma_semiorbis]_KAH0532554.1        ...................EGVDGHEEDV
[Trichoderma_asperellum_CBS_433.97]_XP_0    RTFNAV.AFYLDVADMLAKEGL.......
[Trichoderma_asperelloides]_KAH8131048.1    RTFNAV.AFYLDVADMLAKEGL.......
[Trichoderma_arundinaceum]_RFU75476.1       RTFNAV.AFYLDVADMLAKEGL.......
[Trichoderma_gracile]_KAH0498535.1          .............................
[Bacillus_licheniformis]_CAB95850.2_RN2     QNTN.........................
[Trichosporon_asahii_var._asahii]_AGN981    GPLPKCKPFFLAI................
```
