# Supplementary material for: Identification of a Novel Lipase with AHSMG Pentapeptide in Hypocreales and Glomerellales Filamentous Fungi
Source: Int J Mol Sci. 2022 Aug 19;23(16):9367. doi: 10.3390/ijms23169367 (PMC9408867; doi:10.3390/ijms23169367)
Supplement: Supplementary file 1 [file ijms-23-09367-s001.zip › Figure S5.Multialignment of ThaL and other AHSMG-lipases (cluster III) with lipases from other clusters.pdf]

```
[Candida_albicans]_ADP21191.1                   ............................................................
[Parastagonospora_nodorum_SN15]_XP_001800960.1  ............................................................
[Talaromyces_cellulolyticus]_GAM42581.1         ............................................................
[Fusarium_oxysporum]_ABR12479.1                 ............................................................
[Verticillium_dahliae_VdLs.17]_EGY21687.1       ............................................................
[Melampsora_                                    ............................................................
[Cryptococcus_gattii                            ............................................................
[Fusarium_vanettenii_77-13-4]_XP_003048915.1    MRIYLAERTPRVIGKKLAETAVQFSQQAEKLSHQHPTGQDPSVAVEAQFVTPQDPVIVTS
[Geobacillus_sp._WSUCF1]_EPR29489.1_2Z5G        ............................................................
[Trichoderma_gracile]_KAH0498535.1              ............................................................
[Trichoderma_semiorbis]_KAH0532554.1            ............................................................
[Trichoderma_virens_FT-333]_UKZ73321.1          ............................................................
[Trichoderma_lentiforme]_KAF3077685.1           ............................................................
[Trichoderma_simmonsii]_QYS94233.1              ............................................................
[Trichoderma_guizhouense]_OPB43634.1            ............................................................
[Trichoderma_harzianum]_ThaL                    ............................................................
[Trichoderma_harzianum]_KKO99473.1              ............................................................
[Trichoderma_arundinaceum]_RFU75476.1           ............................................................
[Trichoderma_asperellum_CBS_433.97]_XP_0247565  ............................................................
[Trichoderma_asperelloides]_KAH8131048.1        ............................................................

[Candida_albicans]_ADP21191.1                   ............................................................
[Parastagonospora_nodorum_SN15]_XP_001800960.1  ............................................................
[Talaromyces_cellulolyticus]_GAM42581.1         ............................................................
[Fusarium_oxysporum]_ABR12479.1                 ............................................................
[Verticillium_dahliae_VdLs.17]_EGY21687.1       ............................................................
[Melampsora_                                    ............................................................
[Cryptococcus_gattii                            ............................................................
[Fusarium_vanettenii_77-13-4]_XP_003048915.1    DGQRLPGVPLQEAHKLNVLREELQGEPKTVEIKEGDEVKEKISNVESADTRHRKVVQLPE
[Geobacillus_sp._WSUCF1]_EPR29489.1_2Z5G        ............................................................
[Trichoderma_gracile]_KAH0498535.1              ............................................................
[Trichoderma_semiorbis]_KAH0532554.1            ............................................................
[Trichoderma_virens_FT-333]_UKZ73321.1          ............................................................
[Trichoderma_lentiforme]_KAF3077685.1           ............................................................
[Trichoderma_simmonsii]_QYS94233.1              ............................................................
[Trichoderma_guizhouense]_OPB43634.1            ............................................................
[Trichoderma_harzianum]_ThaL                    ............................................................
[Trichoderma_harzianum]_KKO99473.1              ............................................................
[Trichoderma_arundinaceum]_RFU75476.1           ............................................................
[Trichoderma_asperellum_CBS_433.97]_XP_0247565  ............................................................
[Trichoderma_asperelloides]_KAH8131048.1        ............................................................

                                                                                        1        10
[Candida_albicans]_ADP21191.1                   ........................................MLYL..ILFLIAPIYAGLIF
[Parastagonospora_nodorum_SN15]_XP_001800960.1  ........................................MK........LAAVLALLPL
[Talaromyces_cellulolyticus]_GAM42581.1         ......................................MHLNILPIYFFVISRL.....I
[Fusarium_oxysporum]_ABR12479.1                 ..................................................MLLLPLLSAI
[Verticillium_dahliae_VdLs.17]_EGY21687.1       ........................................MRFIACGFSGLVRFASLLAL
[Melampsora_                                    ......MSRMKT......K.........SPQ.............VCL..........LTL
[Cryptococcus_gattii                            ............................................MYI.............
[Fusarium_vanettenii_77-13-4]_XP_003048915.1    QQNGASMQKSTTNGTLRKAMPPSHTNPLFPPLPLYGPPSMLRNIQCLFFRISAFFLSLAF
[Geobacillus_sp._WSUCF1]_EPR29489.1_2Z5G        ............................................................
[Trichoderma_gracile]_KAH0498535.1              ............................................................
[Trichoderma_semiorbis]_KAH0532554.1            ............................................................
[Trichoderma_virens_FT-333]_UKZ73321.1          ............................................................
[Trichoderma_lentiforme]_KAF3077685.1           ............................................................
[Trichoderma_simmonsii]_QYS94233.1              ............................................................
[Trichoderma_guizhouense]_OPB43634.1            ............................................................
[Trichoderma_harzianum]_ThaL                    ............................................................
[Trichoderma_harzianum]_KKO99473.1              ............................................................
[Trichoderma_arundinaceum]_RFU75476.1           ............................................................
[Trichoderma_asperellum_CBS_433.97]_XP_0247565  ............................................................
[Trichoderma_asperelloides]_KAH8131048.1        ............................................................

                                                20              30         40        50               60
[Candida_albicans]_ADP21191.1                   PTK......PSSDPFYNPPKGFE.NAAVGDILQSRATPKSI.......TGGFTPLKIQNS
[Parastagonospora_nodorum_SN15]_XP_001800960.1  A........................LAAPAQIQKRAAPTIIAPAAQATVGGKDLLGVE..
[Talaromyces_cellulolyticus]_GAM42581.1         S........................SSAGSQTAGRPNDQNF...................
[Fusarium_oxysporum]_ABR12479.1                 T........................LAVASPVALDDYVNSL.......EERAVGVTTT..
[Verticillium_dahliae_VdLs.17]_EGY21687.1       A........................LNVGATISQRPLGGGSD......DDGRQPVSVA..
[Melampsora_                                    LSFLSPQVYGFQVPFLWTNP.SRSTTNSQGSLNFQIQHA..........IH...IGTHRY
[Cryptococcus_gattii                            PG..ALRLSSYVLPFLSSPYPPTQSSPETSTISFKPVHA..........HGHAFVHNAST
[Fusarium_vanettenii_77-13-4]_XP_003048915.1    LGVIVLGALFTSIPSLWRNIFYR........LTFR.........................
[Geobacillus_sp._WSUCF1]_EPR29489.1_2Z5G        ............................................................
[Trichoderma_gracile]_KAH0498535.1              ............................................................
[Trichoderma_semiorbis]_KAH0532554.1            ............................................................
[Trichoderma_virens_FT-333]_UKZ73321.1          ............................................................
[Trichoderma_lentiforme]_KAF3077685.1           ............................................................
[Trichoderma_simmonsii]_QYS94233.1              ............................................................
[Trichoderma_guizhouense]_OPB43634.1            ............................................................
[Trichoderma_harzianum]_ThaL                    ............................................................
[Trichoderma_harzianum]_KKO99473.1              ............................................................
[Trichoderma_arundinaceum]_RFU75476.1           ............................................................
[Trichoderma_asperellum_CBS_433.97]_XP_0247565  ............................................................
[Trichoderma_asperelloides]_KAH8131048.1        ............................................................
```

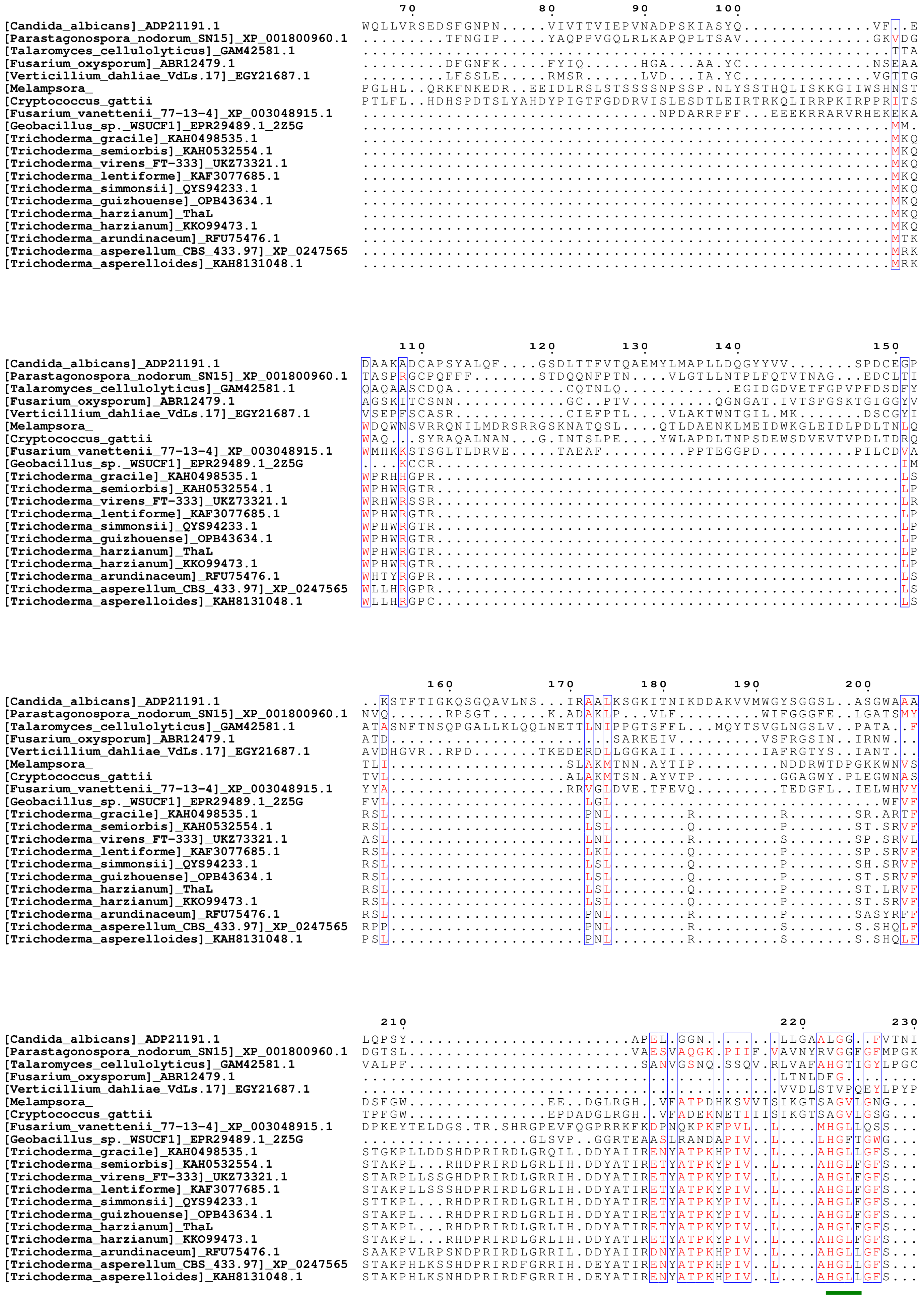

70 80 90 100
[Candida_albicans]_ADP21191.1 WQLLVRSEDSFGNPN.....VIVTTVIEPVNADPSKIASYQ..............VF..E
[Parastagonospora_nodorum_SN15]_XP_001800960.1 .........TFNGIP.....YAQPPVGQLRLKAPQPLTSAV..............GKVDG
[Talaromyces_cellulolyticus]_GAM42581.1 ....TTA
[Fusarium_oxysporum]_ABR12479.1 .........DFGNFK.....FYIQ......HGA...AA.YC..............NSEAA
[Verticillium_dahliae_VdLs.17]_EGY21687.1 .........LFSSLE.....RMSR......LVD...IA.YC..............VGTTG
[Melampsora_ PGLHL..QRKFNKEDR..EEIDLRSLSTSSSSNPSSP.NLYSSTHQLISKKGIIWSHNST
[Cryptococcus_gattii PTLFL..HDHSPDTSLYAHDYPIGTFGDDRVISLESDTLEIRTRKQLIRRPKIRPPRITS
[Fusarium_vanettenii_77-13-4]_XP_003048915.1 ....NPDARRPFF...EEEKRRARVRHEKEKA
[Geobacillus_sp._WSUCF1]_EPR29489.1_2Z5G ....MM.
[Trichoderma_gracile]_KAH0498535.1 ....MKQ
[Trichoderma_semiorbis]_KAH0532554.1 ....MKQ
[Trichoderma_virens_FT-333]_UKZ73321.1 ....MKQ
[Trichoderma_lentiforme]_KAF3077685.1 ....MKQ
[Trichoderma_simmonsii]_QYS94233.1 ....MKQ
[Trichoderma_guizhouense]_OPB43634.1 ....MKQ
[Trichoderma_harzianum]_ThaL ....MKQ
[Trichoderma_harzianum]_KKO99473.1 ....MKQ
[Trichoderma_arundinaceum]_RFU75476.1 ....MTK
[Trichoderma_asperellum_CBS_433.97]_XP_0247565 ....MRK
[Trichoderma_asperelloides]_KAH8131048.1 ....MRK
110 120 130 140 150
[Candida_albicans]_ADP21191.1 DAAKADCAPSYALQF....GSDLTTFVTQAEMYLMAPLLDQGYYVV.......SPDCEGP
[Parastagonospora_nodorum_SN15]_XP_001800960.1 TASPRGCPQFFF.......STDQQNFPTN....VLGTLLNTPLFQTVTNAG...EDCLTI
[Talaromyces_cellulolyticus]_GAM42581.1 QAQAASCDQA............CQTNLQ...........EGIDGDVETFGPVPFDSDFY
[Fusarium_oxysporum]_ABR12479.1 AGSKITCSNN............GC..PTV.........QGNGAT.IVTSFGSKTGIGGYV
[Verticillium_dahliae_VdLs.17]_EGY21687.1 VSEPFSCASR............CIEFPTL....VLAKTWNTGIL.MK.......DSCGYI
[Melampsora_ WDQWNSVRRQNILMDRSRRGSKNATQSL....QTLDAENKLMEIDWKGLEIDLPDLTNLQ
[Cryptococcus_gattii WAQ...SYRAQALNAN...G.INTSLPE....YWLAPDLTNPSDEWSDVEVTVPDLTDRQ
[Fusarium_vanettenii_77-13-4]_XP_003048915.1 WMHKKSTSGLTLDRVE.....TAEAF.........PPTEGGPD..........PILCDVA
[Geobacillus_sp._WSUCF1]_EPR29489.1_2Z5G ....KCCR...IM
[Trichoderma_gracile]_KAH0498535.1 WPRHHGPR...LS
[Trichoderma_semiorbis]_KAH0532554.1 WPHWRGTR...LP
[Trichoderma_virens_FT-333]_UKZ73321.1 WRHWRSSR...LR
[Trichoderma_lentiforme]_KAF3077685.1 WPHWRGTR...LP
[Trichoderma_simmonsii]_QYS94233.1 WPHWRGTR...LP
[Trichoderma_guizhouense]_OPB43634.1 WPHWRGTR...LP
[Trichoderma_harzianum]_ThaL WPHWRGTR...LP
[Trichoderma_harzianum]_KKO99473.1 WPHWRGTR...LP
[Trichoderma_arundinaceum]_RFU75476.1 WHTYRGPR...LS
[Trichoderma_asperellum_CBS_433.97]_XP_0247565 WLLHRGPR...LS
[Trichoderma_asperelloides]_KAH8131048.1 WLLHRGPC...LS
160 170 180 190 200
[Candida_albicans]_ADP21191.1 ..KSTFTIGKQSGQAVLNS...IRAALKSGKITNIKDDAKVVMWGYSGGSL..ASGWAAA
[Parastagonospora_nodorum_SN15]_XP_001800960.1 NVQ......RPSGT......K.ADAKLP...VLF.........WIFGGGFE..LGATSMY
[Talaromyces_cellulolyticus]_GAM42581.1 ATASNFTNSQPGALLKLQQLNETTLNIPPGTSFFL....MQYTSVGLNGSLV..PATA..F
[Fusarium_oxysporum]_ABR12479.1 ATD......................SARKEIV.........VSFRGSIN..IRNW...
[Verticillium_dahliae_VdLs.17]_EGY21687.1 AVDHGVR..RPD.......TKEDERDLLGGKAII.........IAFRGTYS..IANT...
[Melampsora_ TLI.................SLAKMTNN.AYTIP.........NDDRWTDPGKKWNVS
[Cryptococcus_gattii TVL.................ALAKMTSN.AYVTP.........GGAGWY.PLEGWNAS
[Fusarium_vanettenii_77-13-4]_XP_003048915.1 YYA.................RRVGLDVE.TFEVQ.........TEDGFL..IELWHVY
[Geobacillus_sp._WSUCF1]_EPR29489.1_2Z5G FVL.................LGL.............................WFVF
[Trichoderma_gracile]_KAH0498535.1 RSL.................PNL........R.........R.......SR.ARTF
[Trichoderma_semiorbis]_KAH0532554.1 RSL.................LSL........Q.........P.......ST.SRVF
[Trichoderma_virens_FT-333]_UKZ73321.1 ASL.................LNL........R.........S.......SP.SRVL
[Trichoderma_lentiforme]_KAF3077685.1 RSL.................LKL........Q.........P.......SP.SRVF
[Trichoderma_simmonsii]_QYS94233.1 RSL.................LSL........Q.........P.......SH.SRVF
[Trichoderma_guizhouense]_OPB43634.1 RSL.................LSL........Q.........P.......ST.SRVF
[Trichoderma_harzianum]_ThaL RSL.................LSL........Q.........P.......ST.LRVF
[Trichoderma_harzianum]_KKO99473.1 RSL.................LSL........Q.........P.......ST.SRVF
[Trichoderma_arundinaceum]_RFU75476.1 RSL.................PNL........R.........P.......SASYRFF
[Trichoderma_asperellum_CBS_433.97]_XP_0247565 RPP.................PNL........R.........S.......S.SHQLF
[Trichoderma_asperelloides]_KAH8131048.1 PSL.................PNL........R.........S.......S.SHQLF
210 220 230
[Candida_albicans]_ADP21191.1 LQPSY......................APEL.GGN.......LLGAALGG..FVTNI
[Parastagonospora_nodorum_SN15]_XP_001800960.1 DGTSL......................VAESVAQGK.PIIF.VAVNYRVGGFGFMPGK
[Talaromyces_cellulolyticus]_GAM42581.1 VALPF......................SANVGSNQ.SSQV.RLVAFAHGTIGYLPGC
[Fusarium_oxysporum]_ABR12479.1 ....LTNLDFG.......
[Verticillium_dahliae_VdLs.17]_EGY21687.1 ....VVDLSTVPQEYLPYP
[Melampsora_ DSFGW.............EE..DGLRGH..VFATPDHKSVVISIKGTSAGVLGNG...
[Cryptococcus_gattii TPFGW.............EPDADGLRGH..VFADEKNETIIISIKGTSAGVLGSG...
[Fusarium_vanettenii_77-13-4]_XP_003048915.1 DPKEYTELDGS.TR.SHRGPEVFQGPRRKFKDPNQKPKFPVL..L....MHGLLQSS...
[Geobacillus_sp._WSUCF1]_EPR29489.1_2Z5G ...............GLSVP..GGRTEAASLRANDAPIV..L....LHGFTGWG...
[Trichoderma_gracile]_KAH0498535.1 STGKPLLDDSHDPRIRDLGRQIL.DDYAIIRENYATPKHPIV..L....AHGLLGFS...
[Trichoderma_semiorbis]_KAH0532554.1 STAKPL...RHDPRIRDLGRLIH.DDYATIRETYATPKYPIV..L....AHGLFGFS...
[Trichoderma_virens_FT-333]_UKZ73321.1 STARPLLSSGHDPRIRDLGRRIH.DDYATIRETYATPKYPIV..L....AHGLFGFS...
[Trichoderma_lentiforme]_KAF3077685.1 STAKPLLSSSHDPRIRDLGRLIH.DDYATIRETYATPKYPIV..L....AHGLFGFS...
[Trichoderma_simmonsii]_QYS94233.1 STTKPL...RHDPRIRDLGRLIH.DDYATIRETYATPKYPIV..L....AHGLFGFS...
[Trichoderma_guizhouense]_OPB43634.1 STAKPL...RHDPRIRDLGRLIH.DDYATIRETYATPKYPIV..L....AHGLFGFS...
[Trichoderma_harzianum]_ThaL STAKPL...RHDPRIRDLGRLIH.DDYATIRETYATPKYPIV..L....AHGLFGFS...
[Trichoderma_harzianum]_KKO99473.1 STAKPL...RHDPRIRDLGRLIH.DDYATIRETYATPKYPIV..L....AHGLFGFS...
[Trichoderma_arundinaceum]_RFU75476.1 SAAKPVLRPSNDPRIRDLGRRIL.DDYAIIRDNYATPKHPIV..L....AHGLLGFS...
[Trichoderma_asperellum_CBS_433.97]_XP_0247565 STAKPHLKSSHDPRIRDFGRRIH.DEYATIRENYATPKHPIV..L....AHGLLGFS...
[Trichoderma_asperelloides]_KAH8131048.1 STAKPHLKSNHDPRIRDFGRRIH.DEYATIRENYATPKHPIV..L....AHGLLGFS...

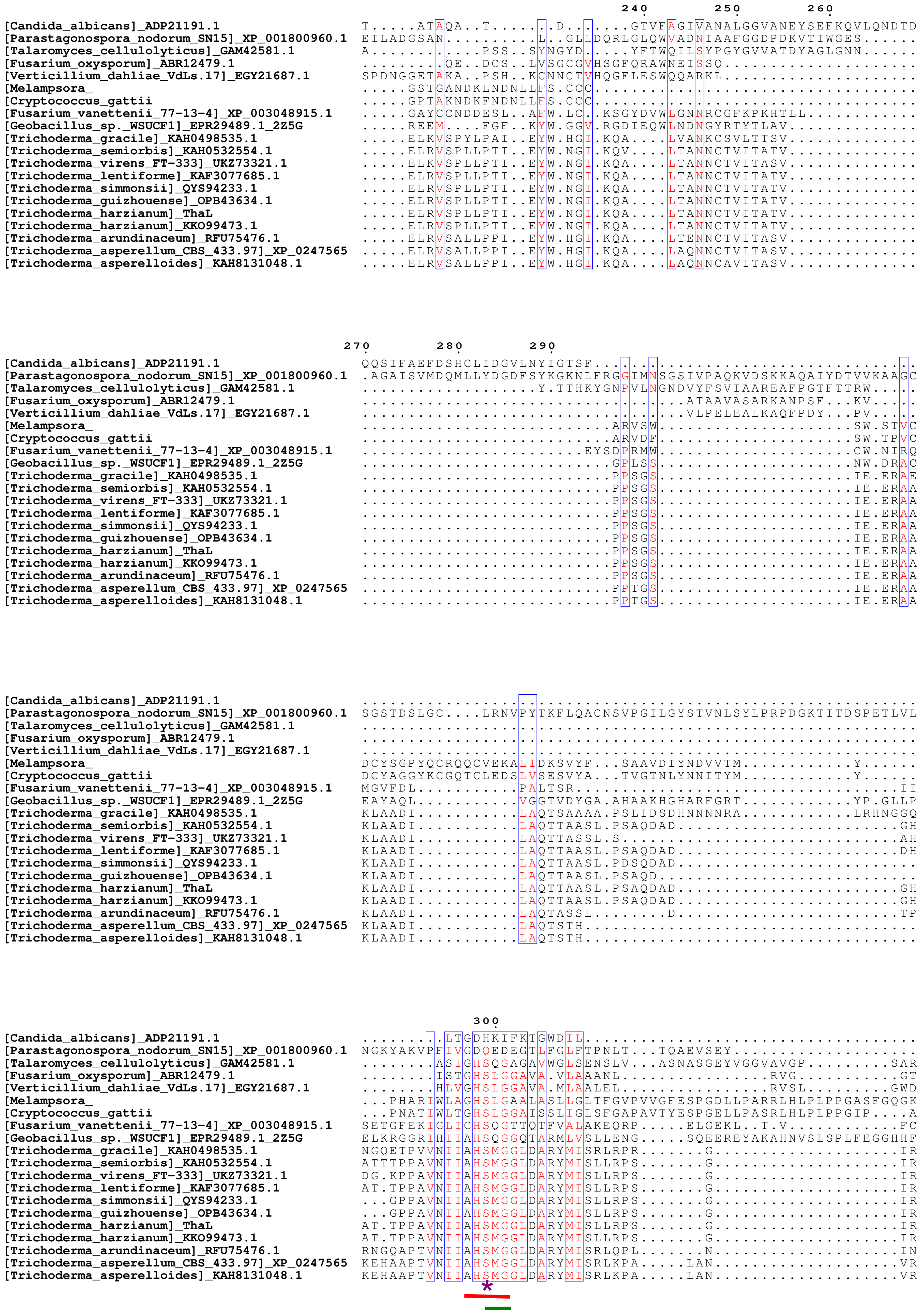
240
250
260
270
280
290
300
[Candida_albicans]_ADP21191.1
[Parastagonospora_nodorum_SN15]_XP_001800960.1
[Talaromyces_cellulolyticus]_GAM42581.1
[Fusarium_oxysporum]_ABR12479.1
[Verticillium_dahliae_VdLs.17]_EGY21687.1
[Melampsora_
[Cryptococcus_gattii
[Fusarium_vanettenii_77-13-4]_XP_003048915.1
[Geobacillus_sp._WSUCF1]_EPR29489.1_2Z5G
[Trichoderma_gracile]_KAH0498535.1
[Trichoderma_semiorbis]_KAH0532554.1
[Trichoderma_virens_FT-333]_UKZ73321.1
[Trichoderma_lentiforme]_KAF3077685.1
[Trichoderma_simmonsii]_QYS94233.1
[Trichoderma_guizhouense]_OPB43634.1
[Trichoderma_harzianum]_ThaL
[Trichoderma_harzianum]_KKO99473.1
[Trichoderma_arundinaceum]_RFU75476.1
[Trichoderma_asperellum_CBS_433.97]_XP_0247565
[Trichoderma_asperelloides]_KAH8131048.1

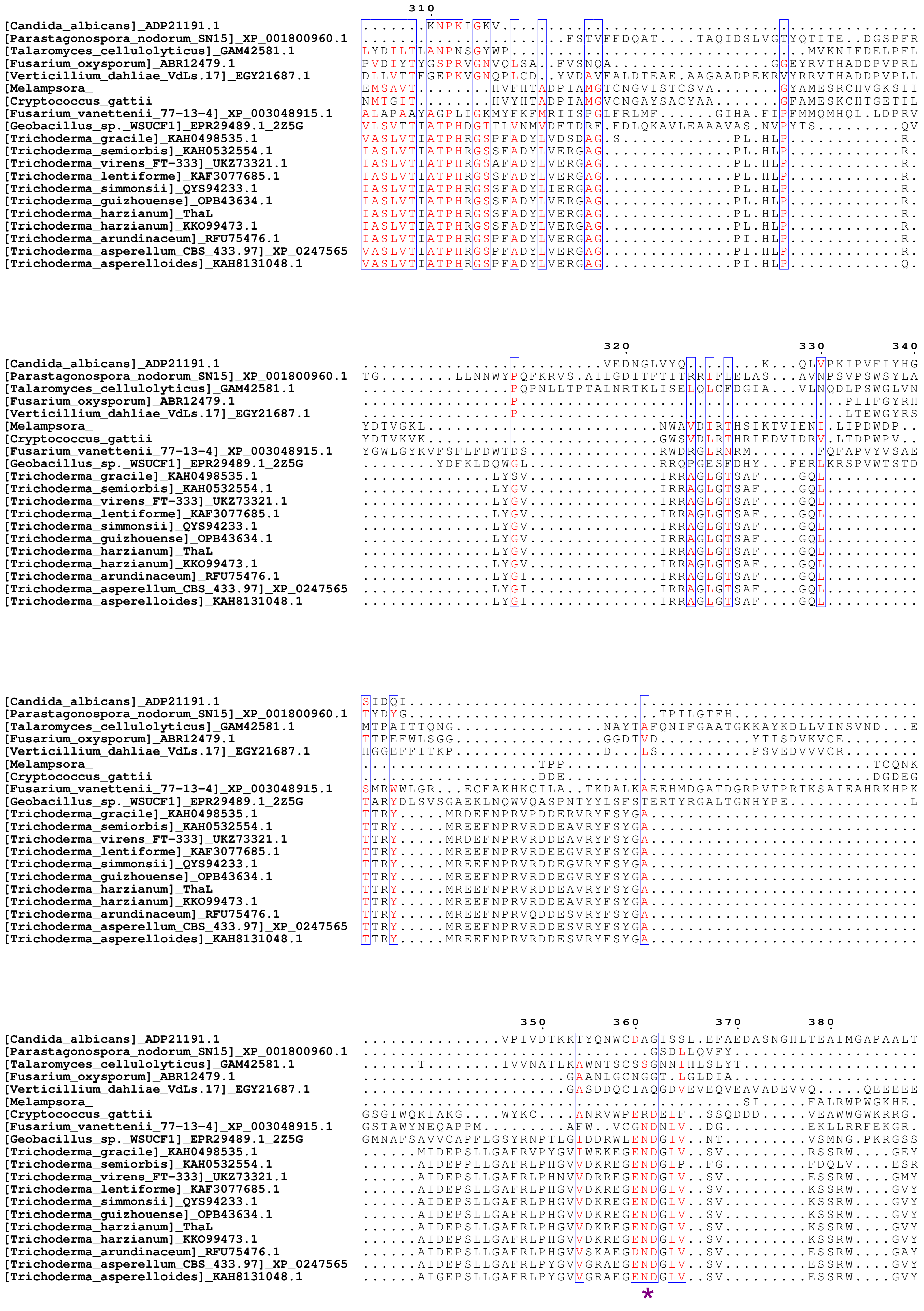
310
320
330
340
350
360
370
380
[Candida_albicans]_ADP21191.1
[Parastagonospora_nodorum_SN15]_XP_001800960.1
[Talaromyces_cellulolyticus]_GAM42581.1
[Fusarium_oxysporum]_ABR12479.1
[Verticillium_dahliae_VdLs.17]_EGY21687.1
[Melampsora_
[Cryptococcus_gattii
[Fusarium_vanettenii_77-13-4]_XP_003048915.1
[Geobacillus_sp._WSUCF1]_EPR29489.1_2Z5G
[Trichoderma_gracile]_KAH0498535.1
[Trichoderma_semiorbis]_KAH0532554.1
[Trichoderma_virens_FT-333]_UKZ73321.1
[Trichoderma_lentiforme]_KAF3077685.1
[Trichoderma_simmonsii]_QYS94233.1
[Trichoderma_guizhouense]_OPB43634.1
[Trichoderma_harzianum]_ThaL
[Trichoderma_harzianum]_KKO99473.1
[Trichoderma_arundinaceum]_RFU75476.1
[Trichoderma_asperellum_CBS_433.97]_XP_0247565
[Trichoderma_asperelloides]_KAH8131048.1
*

```
                                                   390       400                 410       420       430
[Candida_albicans]_ADP21191.1                      WIIDRFDGKQT..............VSGCQHIQRFSNLEYPNIPSSIANYFKAAMD....
[Parastagonospora_nodorum_SN15]_XP_001800960.1     .......GVLPNN..........AAKSIRGYYYSFIYELDPNKGNNLDDWPQWSQSKKLL
[Talaromyces_cellulolyticus]_GAM42581.1            .......GGL...................SHT.....QSFPAS...SPEWLQWLDN..QF
[Fusarium_oxysporum]_ABR12479.1                    ......................................................AHLHYF
[Verticillium_dahliae_VdLs.17]_EGY21687.1          EQEERLAGAVEGT..........AP.QERRWV.....KRFPSVPARFKLWQLFFAHRDYF
[Melampsora_                                       NGTEPCEG..NKP....KNSIPEASVQECEEG.........................DCF
[Cryptococcus_gattii                               RKKEREGK..EDPEWEKHGGVP.KAISEEDCV.........................DCY
[Fusarium_vanettenii_77-13-4]_XP_003048915.1       ...EPFVDVVH...........SKTIPEYEHL.........................DVI
[Geobacillus_sp._WSUCF1]_EPR29489.1_2Z5G           DRIVPYDGTLKKGVWN......DMGTYNVDHL.........................EII
[Trichoderma_gracile]_KAH0498535.1                 ......KGTL................VGVSHL.........................DLI
[Trichoderma_semiorbis]_KAH0532554.1               ....EVDGEG................VDGHEE.........................DV.
[Trichoderma_virens_FT-333]_UKZ73321.1             ......KGTL................MGVSHL.........................DLI
[Trichoderma_lentiforme]_KAF3077685.1              ......KGTL................MGVSHL.........................DLI
[Trichoderma_simmonsii]_QYS94233.1                 ......KGTL................MGVSHL.........................DLI
[Trichoderma_guizhouense]_OPB43634.1               ......KGTL................MGVSHL.........................DLI
[Trichoderma_harzianum]_ThaL                       ......KGTL................MGVSHL.........................DLI
[Trichoderma_harzianum]_KKO99473.1                 ......KGTL................MGVSHL.........................DLI
[Trichoderma_arundinaceum]_RFU75476.1              ......KGTL................MGVSHL.........................DLI
[Trichoderma_asperellum_CBS_433.97]_XP_0247565     ......QGTL................MGVSHL.........................DLI
[Trichoderma_asperelloides]_KAH8131048.1           ......QGTL................MGVSHL.........................DLI
                                                                                 *
```

```
                                                               440       450                 460
[Candida_albicans]_ADP21191.1                      ....VVLLLGLGPDVQKDQVNPEGIKKLGS...............IEMRWL.........
[Parastagonospora_nodorum_SN15]_XP_001800960.1     NF..........................GANRNSLLDDN...FRKDTEDWLKVNMFS...
[Talaromyces_cellulolyticus]_GAM42581.1            KSGGTVNATGCSISTATPLISPIGIVSNG...............................
[Fusarium_oxysporum]_ABR12479.1                    QATDACNAGGF..S.WRRYRSAESVDKRATMTDAELEKKLNSYVQMDKEYVKNNQARS..
[Verticillium_dahliae_VdLs.17]_EGY21687.1          WRIGLCVPGGDPAN.WGREKYPDGQ...GGDDDARLEEL.....................
[Melampsora_                                       K..........................................WEFKDDWDDEASL..DC
[Cryptococcus_gattii                               K..........................................WEFG.DWDK........
[Fusarium_vanettenii_77-13-4]_XP_003048915.1       WAMDA..........................VDQVFKEVREVLWKTC.DVRDICRVPEGC
[Geobacillus_sp._WSUCF1]_EPR29489.1_2Z5G           GVDP.................................................NPS....
[Trichoderma_gracile]_KAH0498535.1                 N...........................................................
[Trichoderma_semiorbis]_KAH0532554.1               ............................................................
[Trichoderma_virens_FT-333]_UKZ73321.1             NWSNR....................................ARWTVR.EWMGMRRT....
[Trichoderma_lentiforme]_KAF3077685.1              NWSNR....................................ARWTVR.EWMGMRRT....
[Trichoderma_simmonsii]_QYS94233.1                 NWSNR....................................ARWTVR.EWMGMRRT....
[Trichoderma_guizhouense]_OPB43634.1               NWSNR....................................ARWTVR.EWMGMRRT....
[Trichoderma_harzianum]_ThaL                       NWSNR....................................ARWTVR.EWMGMRRT....
[Trichoderma_harzianum]_KKO99473.1                 NWSNR....................................ARWTVR.EWMGMRRT....
[Trichoderma_arundinaceum]_RFU75476.1              NWSNR....................................AAWTMR.EWMGMKRT....
[Trichoderma_asperellum_CBS_433.97]_XP_0247565     NWSNR....................................AAWTMR.EWMGMKRT....
[Trichoderma_asperelloides]_KAH8131048.1           NWSNR....................................AAWTMR.EWMGMKRT....
```

```
[Candida_albicans]_ADP21191.1                      ............................
[Parastagonospora_nodorum_SN15]_XP_001800960.1     ............................
[Talaromyces_cellulolyticus]_GAM42581.1            ............................
[Fusarium_oxysporum]_ABR12479.1                    ............................
[Verticillium_dahliae_VdLs.17]_EGY21687.1          ............................
[Melampsora_                                       ............................
[Cryptococcus_gattii                               ............................
[Fusarium_vanettenii_77-13-4]_XP_003048915.1       EHVEPRKPKVSVAEDVIDETQSSSSGEN
[Geobacillus_sp._WSUCF1]_EPR29489.1_2Z5G           ..FDIRAFYLRLAEQLASLQP.......
[Trichoderma_gracile]_KAH0498535.1                 ............................
[Trichoderma_semiorbis]_KAH0532554.1               ............................
[Trichoderma_virens_FT-333]_UKZ73321.1             ..FNAVAFYLDVVDMLAKEGL.......
[Trichoderma_lentiforme]_KAF3077685.1              ..FNAVAFYLDVADMLAKEGL.......
[Trichoderma_simmonsii]_QYS94233.1                 ..FNAVAFYLDVADMLAKEGL.......
[Trichoderma_guizhouense]_OPB43634.1               ..FNAVAFYLDVADMLAKEGL.......
[Trichoderma_harzianum]_ThaL                       ..FNAVAFYLDVADMLAKEGL.......
[Trichoderma_harzianum]_KKO99473.1                 ..FNAVAFYLDVADMLAKEGL.......
[Trichoderma_arundinaceum]_RFU75476.1              ..FNAVAFYLDVADMLAKEGL.......
[Trichoderma_asperellum_CBS_433.97]_XP_0247565     ..FNAVAFYLDVADMLAKEGL.......
[Trichoderma_asperelloides]_KAH8131048.1           ..FNAVAFYLDVADMLAKEGL.......
```
