# Supplementary material for: Identification of a Novel Lipase with AHSMG Pentapeptide in Hypocreales and Glomerellales Filamentous Fungi
Source: Int J Mol Sci. 2022 Aug 19;23(16):9367. doi: 10.3390/ijms23169367 (PMC9408867; doi:10.3390/ijms23169367)
Supplement: Supplementary file 1 [file ijms-23-09367-s001.zip › Supplementary legends.pdf]

Table S1. Description Table of ThaL homologs.

Summary of Scientific name, Accession, Description, Max Score, Total Score, Accession length, Query cover, E value, Percent identity for the first 100 hits in Blastp at GenBank. Description Table was downloaded in CSV format.

Figure S1. ThaL lipase and closest hits in *Trichoderma harzianum.*

Comparison of ThaL amino acid sequence with the proteins 135964 (*T. harzianum* strain CBS 226.95) and GenBank accession KKO99473.1 (*T. harzianum* strain T6776). White letters on red background show identical amino acids; a gap at position 144 shows a threonine (T) in KKO99473.1 and ThaL, that is absent in 135964. Horizontal green lines show the amino acids of the oxyanion; red line, the pentapeptide (AHSMG), and purple asterisks the catalytic triad (S, D, H). On the alignment, spirals show α-helices and black arrows the β-sheets. Multi-alignment was performed in ClustalW and edited in EsPript 3.0.

Figure S2. Multi-alignment of ThaL with known AHSMG-lipases.

ThaL amino acid sequence was aligned with TaLipA (*Trichosporon* asahii, GenBank AGN98126.1), and RN2 (*Bacillus* sp., GenBank ABQ81810.1). The protein 2Z5G (*Geobacillus zalihae*, GenBank EPR29489.1), which is the PDB model with the closest pentapeptide to AHSMG (in this case, AHSQG), was also included. The multi-alignment was performed and edited as mentioned in Figure S1. Standard red letters correspond to conserved regions, but containing similar amino acids; white letters on red background show identical amino acids; dotted lines correspond to gaps.

Figure S3. Multi-alignment of ThaL and its homologs.

S3A) The complete amino acid sequences of the first 100 hits were downloaded. Short sequences were eliminated (KAH6604852.1, KAH8131048.1, UKZ62414.1, and KAH0532554.1) because they prevented the observation of conserved regions; multi-alignment was performed with 96 sequences, which are shown here. One motif, observed in a group of lipases in some species of *Trichoderma* is highlighted in yellow. Figure S3B is similar to S3A, but the lipases TaLipA, RN2 and 2Z5G were included in the alignment in figure S3B.

Figure S4. Individual alignment of ThaL and its top ten homologs with either TaLipA, RN2 or 2Z5G.

S4A) Multi-alignment of amino acid sequences of ThaL and its top ten homologs proteins at GenBank: protein KKO99473.1, KAF3077685.1, QYS94233.1, OPB43634.1, UKZ73321.1, XP_024756506.1, RFU75476.1, KAH8131048.1, KAH0532554.1, and KAH0498535.1, with (A), TaLipA; (B), RN2; and (C) 2Z5G. White letters on red background show identical amino acids; standard red letters correspond to conserved regions containing similar amino acids; dotted lines correspond to gaps. Different patterns of conserved regions are observed in each multi-alignment. Figure S4B is similar to S4A, but the lipases TaLipA, RN2 and 2Z5G were included in the alignment in figure S4B.

Figure S5. Multi-alignment of ThaL and other AHSMG-lipases (cluster III) with lipases from other clusters.

ThaL, its top ten homologs, and 2Z5G (Cluster III) were aligned with the proteins (GenBank codes) ABR12479.1 and EGY21687.1 (cluster I), EGG03572.1 and XP_003192616.1 (cluster II), GAM42581.1 (cluster V), XP_001800960.1 (cluster VI), ADP21191.1 (cluster VIIA), XP_003048915.1 (cluster VIIB) to see similarities and differences of cluster III with lipases from different clusters. The tripeptide GLD, next to the pentapeptide AHSMG, is unique to AHSMG-lipases from the filamentous fungi. Cluster IV was not included because it comprises very large lipases, making alignment difficult and preventing the observation of conserved motifs among clusters. The motifs ASLVTIATPH and ENDGLV are also characteristic of filamentous fungi AHSMG-lipases.
