# Supplementary figures and images for: Identification of a Novel Lipase with AHSMG Pentapeptide in Hypocreales and Glomerellales Filamentous Fungi
Source: Int J Mol Sci. 2022 Aug 19;23(16):9367. doi: 10.3390/ijms23169367 (PMC9408867; doi:10.3390/ijms23169367)

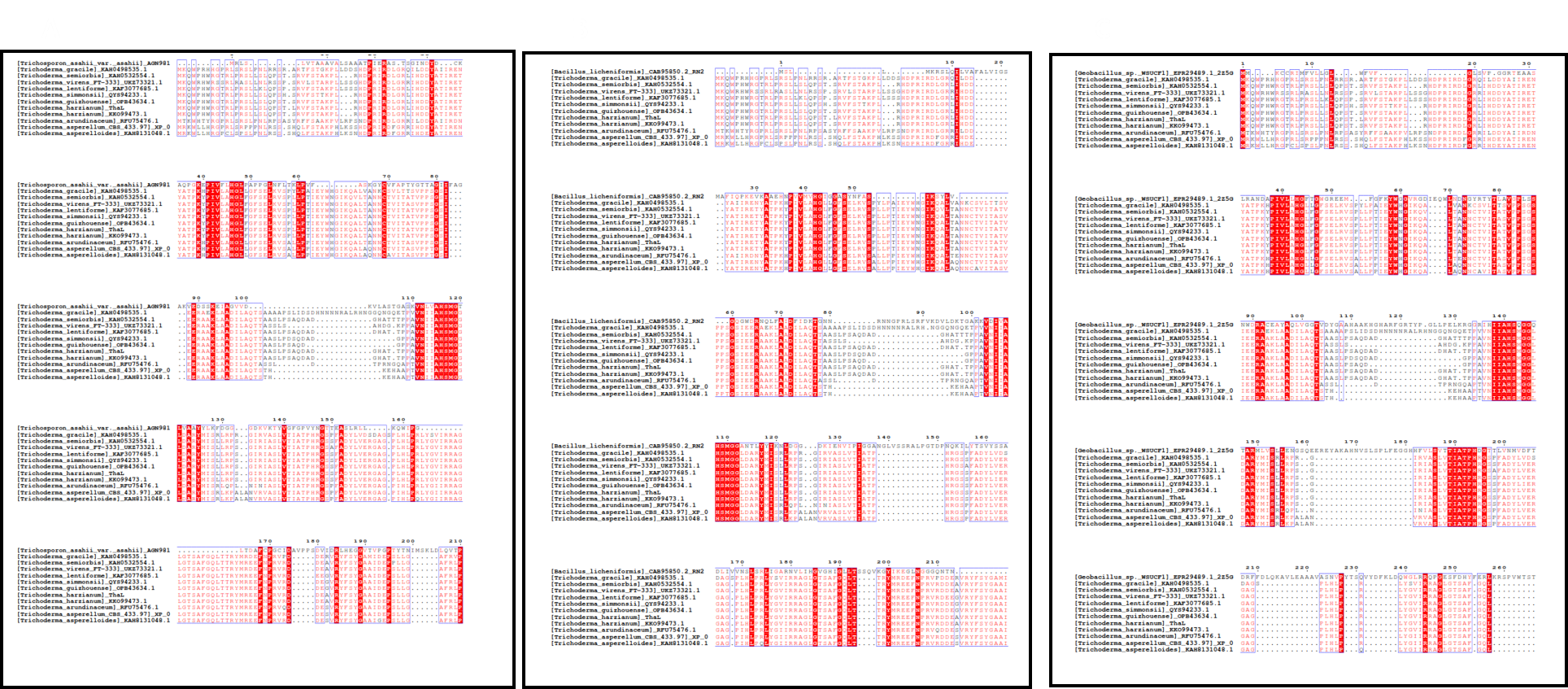

Supplement: Supplementary file 1 [file ijms-23-09367-s001.zip › Figure S4A.Individual alignment of ThaL and its top ten homologs with either TaLipA, RN2 or 2Z5G.tif]
